# Synthesis of Cationic Azatriphenylene Derivatives by Electrochemical Intramolecular Pyridination and Characterization of Their Optoelectronic Properties

**DOI:** 10.1021/acs.orglett.3c01341

**Published:** 2023-05-24

**Authors:** Yushi Ohno, Shogo Ando, Daisuke Furusho, Ryoyu Hifumi, Yuuya Nagata, Ikuyoshi Tomita, Shinsuke Inagi

**Affiliations:** † Department of Chemical Science and Engineering, Tokyo Institute of Technology, 4259 Nagatsuta-cho, Midori-ku, Yokohama, Kanagawa 226-8502, Japan; ‡ Institute for Chemical Reaction Design and Discovery, Hokkaido University, Kita 21 Nishi 10, Kita-Ku, Sapporo, Hokkaido 001-0021, Japan

## Abstract

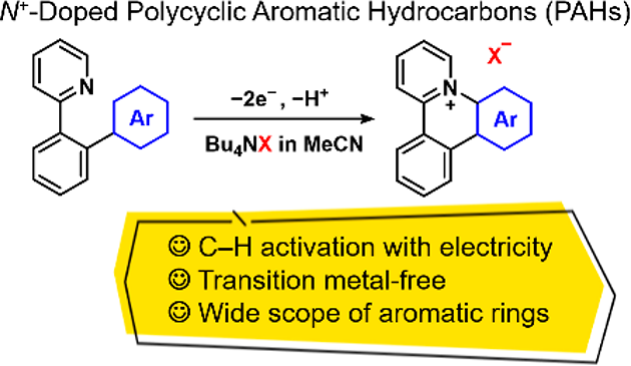

Here, a facile and selective synthesis method for cationic azatriphenylene derivatives was established by electrochemical intramolecular cyclization, where atom-economical C–H pyridination without a transition-metal catalyst or an oxidant is a key step. The proposed protocol is a practical strategy for the late-stage introduction of cationic nitrogen (*N*^+^) into π-electron systems and broadens the scope of molecular design of *N*^+^-doped polycyclic aromatic hydrocarbons.

Polycyclic aromatic hydrocarbons (PAHs) are a class of compounds consisting of two or more fused benzene rings; they are actively studied as a model of two-dimensional (2D) graphene in a wide range of fields, including the pharmaceutical, environmental science, electronics, and materials chemistry fields.^1^ Among PAHs, some disk-shaped molecules, including triphenylenes and hexabenzocoronenes, are known to exhibit liquid-crystalline properties arising from their highly stacked nature.^2^ The bottom-up synthesis of such graphene-like molecules provides access to a group of functional materials such as buckybowls (e.g., corannulenes and sumanenes)^3^ and helical PAHs (e.g., helicenes).^4^ In addition, doping of PAHs with heteroatoms (e.g., chalcogen, nitrogen, phosphine, and boron) has been attracting attention because it can drastically alter their electronic structures.^5^ In heteroatom doping of PAHs, introducing heteroatoms in a desired form at a desired position in the parent skeleton is important; therefore, precise organic synthesis is required to realize an efficient bottom-up approach for preparing heteroatom-doped PAHs.

Among the heteroatom-doped PAHs, cationic nitrogen (*N*^+^)-doped PAHs are known to exhibit functionalities (e.g., stable electrochemical properties, peculiar aggregation behavior,^6^ and fluorescent properties) that have led to their application in organic photocatalysts,^7^ ion-conductive materials,^8^ and bioimaging.^9^ Three main synthesis methods for *N*^+^-doped PAHs have been reported, as summarized in Figure 1a. Among them, photocyclization of polyaryl-substituted pyridinium salts is the most simple but powerful way to prepare *N*^+^-doped PAHs (Figure 1a(i)).^6^ Such Scholl-type reactions proceed by photoirradiation in the presence of an oxidant; however, *N*-arylpyridinium salts with limited synthesis methods are required as precursors. A number of methods based on ring-closing metathesis reactions of *N*-vinylpyridinium salts (Figure 1a(ii))^10^ and [4 + 2] annulation of 2-phenylpyridines or *N*-arylpyridinium salts with alkynes (Figure 1a(iii))^11^ have been proposed. The latter approach can be used to prepare *N*^+^-doped PAHs with imidazole, pyrazole, and triazole units as well as pyridinium units. However, all of these synthesis methods have shortcomings, such as difficulty controlling selectivity and the need to use transition-metal catalysts and chemical oxidants that have adverse environmental impacts. Therefore, a simpler and more environmentally benign synthesis method for *N*^+^-doped PAHs is desired.

**Figure 1:**
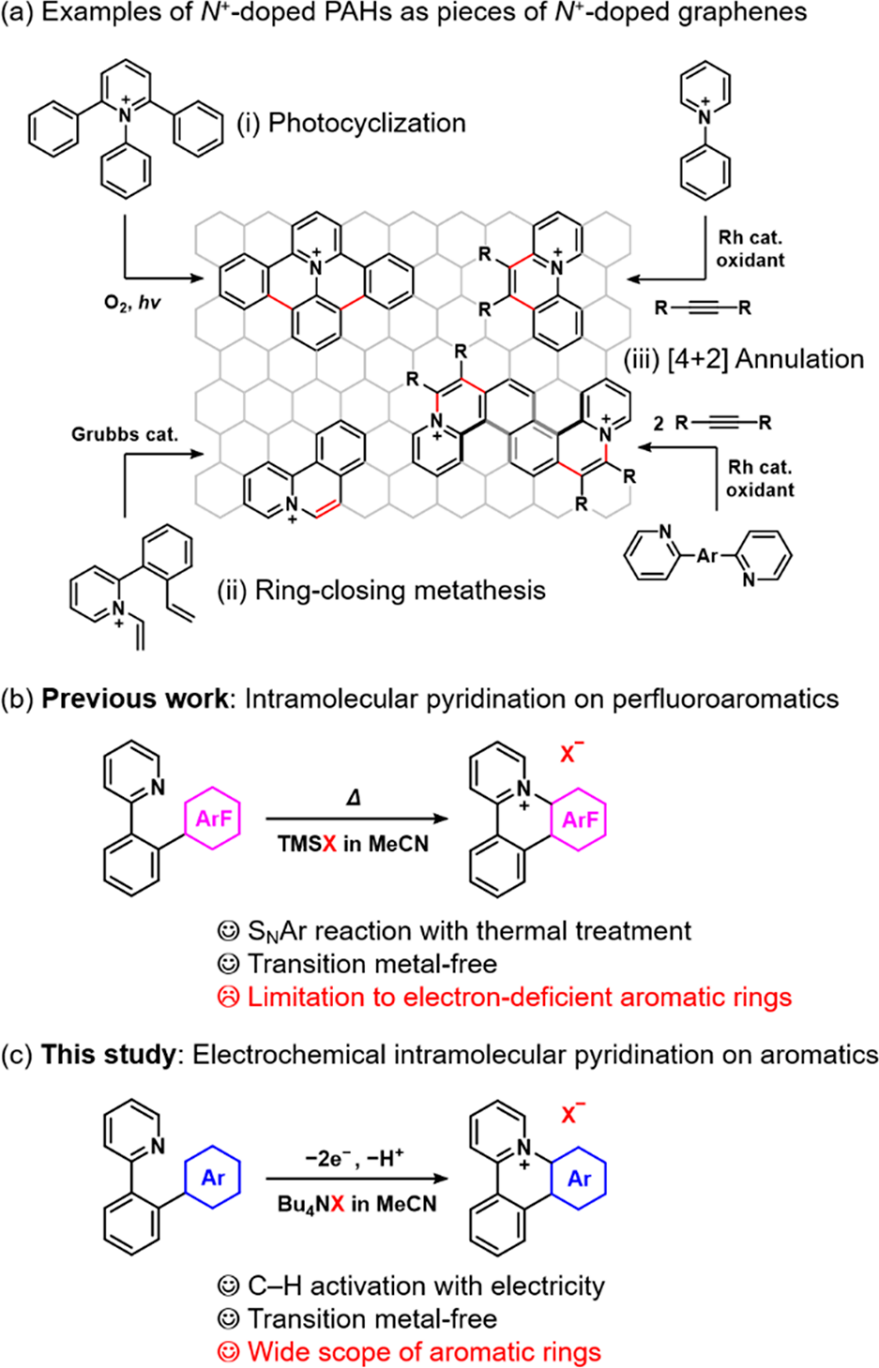
Concept of the present study.

In this context, we focused on the nucleophilic aromatic substitution (S_N_Ar) reaction as a key step in the synthesis of cationic azatriphenylene derivatives. In our previous study, the S_N_Ar reaction was used for the intramolecular cyclization of phenylpyridine derivatives possessing an electron-deficient perfluoroaryl moiety to give the corresponding cationic azatriphenylene derivatives in high yields under moderate conditions without the use of a transition-metal catalyst (Figure 1b).^12^ However, one of the challenges in the synthesis of *N*^+^-doped PAHs via the S_N_Ar reaction is poor functional-group applicability. The development of a new synthesis method is required to overcome this problem and further expand the range of molecular design and accompanying properties.

In the present study, an electro-oxidative C–H activation was used for intramolecular cyclization to afford the corresponding cationic azatriphenylene derivatives (Figure 1c). The key reaction is the anodic pyridination of aromatic compounds reported by Yoshida et al.^13^ The anodic oxidation of an arene generates its radical cation, followed by nucleophilic reaction of pyridines. Total two-electron oxidation and deprotonation gives arylpyridinium derivatives via C–H activation. Therefore, our proposed strategy is not only an efficient and atom-economical method to access *N*^+^-doped PAHs without any transition-metal catalysts or oxidants but also a promising protocol with a wide scope of aromatic rings in both the precursors and products.

2-Phenylpyridine derivative **1a** bearing a methoxyphenyl group was prepared by the Suzuki–Miyaura coupling reaction of 2-(2-bromophenyl)pyridine with 3-methoxyphenylboronic acid. In the cyclic voltammogram of **1a** (Figure S1), an irreversible oxidation wave was observed at a peak potential of 1.5 V (vs saturated calomel electrode (SCE)), suggesting that the electron-rich methoxyphenyl moiety of **1a** preferentially underwent one-electron oxidation, followed by chemical reaction. The highest occupied molecular orbital (HOMO) of **1a**, as calculated by density functional theory (DFT), was localized at the methoxyphenyl moiety (Figure S1), supporting the above estimation of the discharging part for oxidation. Accordingly, the preparative-scale anodic oxidation of **1a** was carried out under constant-current conditions in a divided cell equipped with a carbon felt anode and a Pt plate cathode (1 × 1 cm) under the reported standard conditions for anodic pyridination of arenes (Figure 2a).^13^ The amount of charge (2.6 F/mol) was determined by monitoring the consumption of **1a** by thin-layer chromatography (TLC). The intramolecular pyridination proceeded to yield cationic azatriphenylene derivative **2a** in 84% NMR yield. The counterion of **2a** (BF_4_^–^) was derived from the supporting electrolyte. It was possible to purify the product from the supporting electrolyte (Bu_4_NBF_4_) by washing with cold MeOH, but the isolated yield was considerably reduced (60% isolated yield). The structure of **2a** was determined by NMR, high-resolution mass spectrometry, elemental analysis, and X-ray analysis.

**Figure 2:**
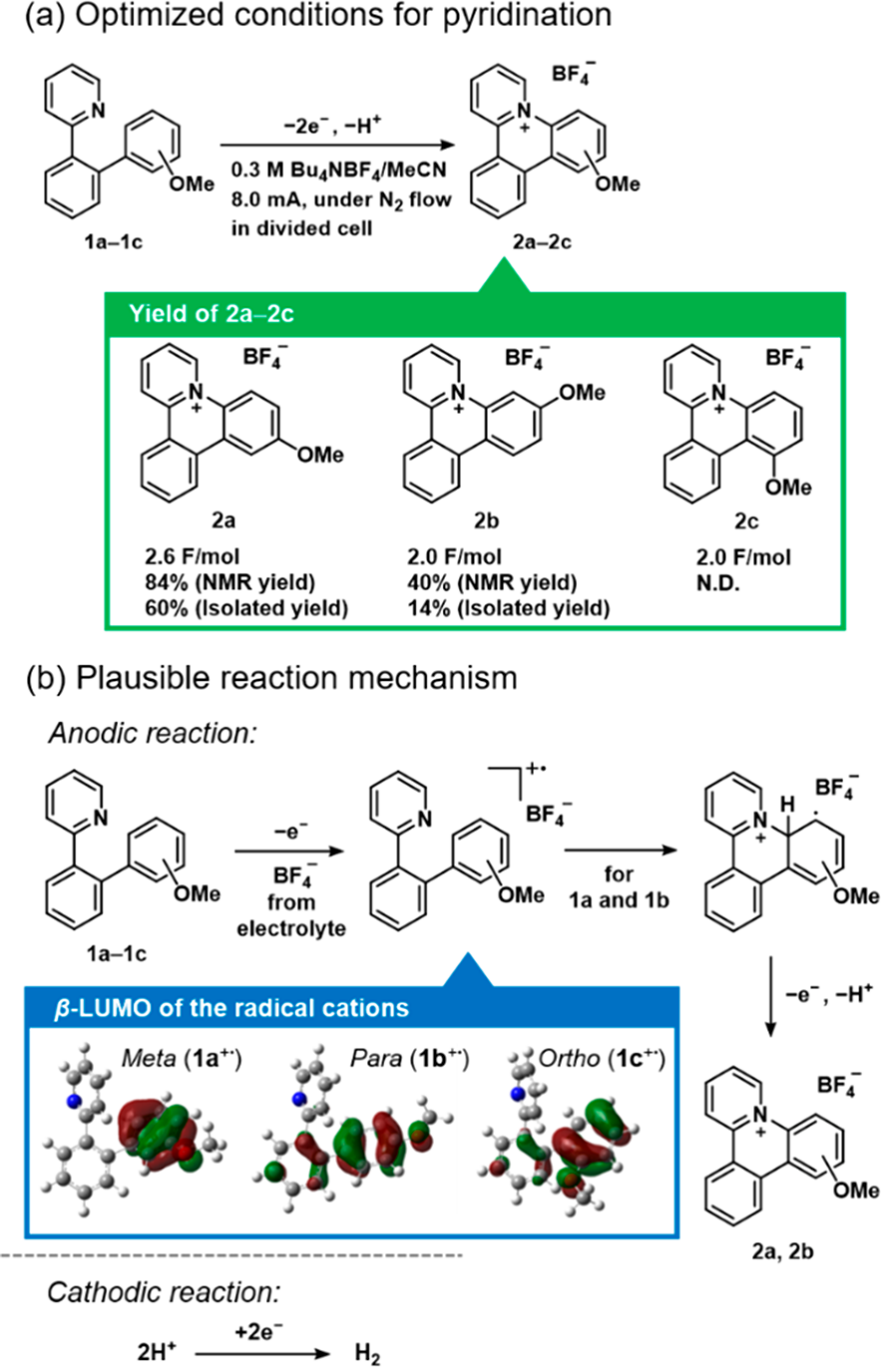
(a) Anodic pyridination of **1a**–**1c**, including yields of cyclized products **2a**–**2c**. (b) The plausible reaction mechanism for the intramolecular pyridination of **1a**–**1c**, with diagrams of the β-LUMO of **1a**–**1c** in their one-electron-oxidized state.

Other regioisomers of **1a** were subjected to the anodic pyridination under the optimized conditions shown in Figure 2a. The intramolecular anodic pyridination of **1b** resulted in poor NMR yield (40%) and isolated yield (14%) of **2b** because of a side reaction that afforded a complex mixture. In the case of **1c**, the corresponding cationic azatriphenylene derivatives (**2c**) was not detected at all; instead, only a complex mixture was obtained.

A plausible reaction mechanism of the intramolecular cyclization of **1** is shown in Figure 2b. As an anodic reaction, one-electron oxidation of **1** generates the radical cation form with its charge localized at the methoxyphenyl moiety, where nucleophilic attack by a pyridine moiety occurs. Further one-electron oxidation and deprotonation (aromatization) results in the formation of **2**. In the cathodic chamber, protons are reduced to generate H_2_ bubbles. Such an intramolecular substitution reaction is easily achieved by the electrochemical umpolung of the methoxyphenyl moiety. The difference in reactivity of the intramolecular anodic pyridination of **1a**–**1c** can be explained on the basis of DFT calculations. Yoshida and co-workers reported on the selectivity in the intermolecular anodic pyridination of arenes, where the position of the large β-LUMO coefficient in the one-electron-oxidized state of arenes corresponds to the position of the nucleophilic attack by pyridine.^13^ On the basis of this knowledge, we conducted DFT calculations of the β-LUMO of **1a**–**1c** in their one-electron-oxidized state. As shown in Figures 2b and S2, large coefficients are observed for the carbons of **1a** and **1b**, where subsequent pyridination occurs to form the corresponding products **2a** and **2b**, respectively. However, no coefficient is observed for the carbon of the **1c** radical cation, where expected intramolecular pyridination should occur. This result strongly supports the experimental fact that **2c** was not obtained.

We next investigated the scope of the intramolecular pyridination (Scheme 1). For **1d**–**1g** bearing two methoxy groups or an ethylenedioxy group, **1d**, **1e**, and **1g** provided the corresponding cationic azatriphenylene derivatives in good NMR yields (80% for **2d**, 78% for **2e**, and 83% for **2g**); by contrast, **2f** was not obtained at all despite complete consumption of starting material **1f**. In this case, spiro compound **2f′** was obtained via pyridination at the *ipso* position of the dimethoxyphenyl moiety (63% NMR yield). A large β-LUMO coefficient is observed for the carbon at the *ipso* position of **1f**; therefore, spirocyclic formation and demethylation occurred to give **2f′** (Figures S2 and S3). Other phenylpyridine derivatives bearing a tolyl (**1h**), phenyl (**2i**), or naphthyl (**2j**) group were also successfully prepared but afforded the products in low to moderate NMR yields (36% for **2h**, 51% for **2i**, and 73% for **2j**). Less-activated arenes are known to not be suitable for this type of anodic coupling reaction, although Waldvogel et al. overcame this problem by using a boron-doped diamond (BDD) electrode.^14^ We could obtain the desired *N*^+^-doped PAHs from precursors lacking methoxy groups because of the advantage of intramolecular cyclization. The regioselectivity for the reaction was also consistent with the DFT simulation results for the β-LUMO coefficients (Figure S2). In particular, the structure of **2j** was also determined by single-crystal X-ray diffraction analysis (vide infra).

**Scheme 1:**
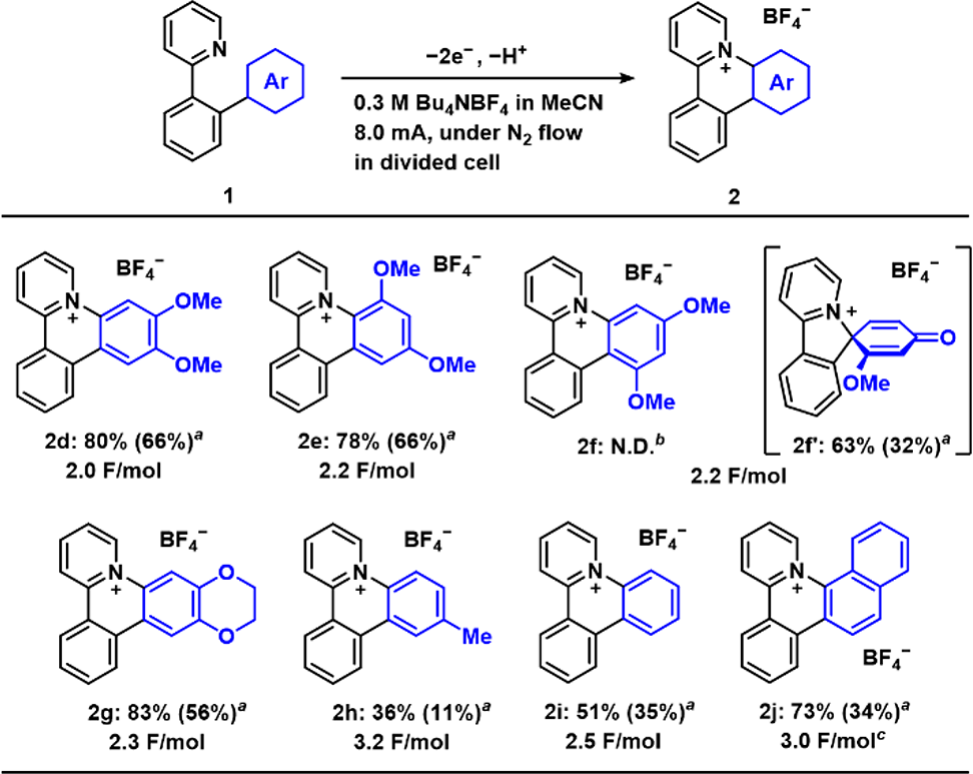
Scope of the Intramolecular Anodic Pyridination to Provide Cationic Azatriphenylene Derivatives NMR yield. Isolated yields are shown in parentheses. Detected. Not detected. 0.3 M Et_4_NBF_4_ was used.

We next investigated the optical and electrochemical properties of the cationic azatriphenylene products. As representative examples, the data for **2a**, **2d**, **2i**, and **2j** are summarized in Figure 3. All other data are summarized in the Supporting Information (Figures S4 and Table S1).

**Figure 3:**
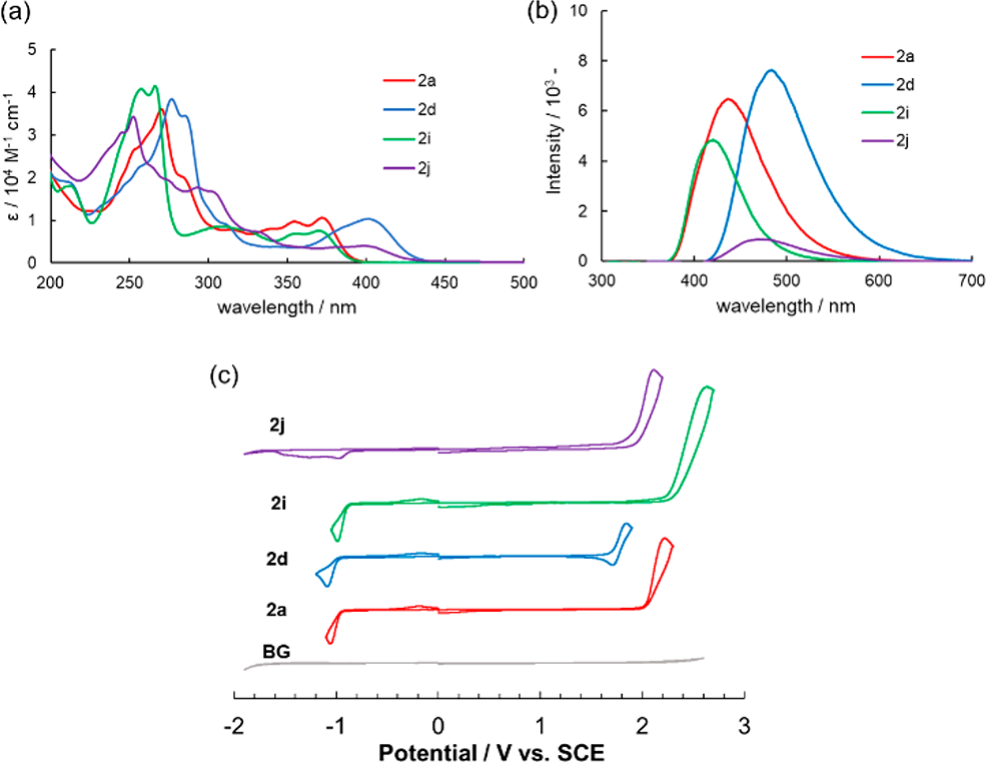
(a) UV–vis absorption spectra and (b) fluorescence spectra for **2a**, **2d**, **2i**, and **2j** in MeCN solutions. (c) Cyclic voltammograms of **2a**, **2d**, **2i**, and **2j** (3 mM) in 0.1 M Bu_4_NPF_6_/MeCN using a Pt working electrode (φ = 3 mm) at a scan rate of 100 mV/s.

UV–vis absorption spectra of the cationic azatriphenylene derivatives in acetonitrile (MeCN) solutions show an absorption maximum at 250–300 nm and a weaker absorption band at 300–450 nm (Figure 3a), which are attributed to the π–π* transition and the intramolecular charge transfer (ICT) transition, respectively, as supported by time-dependent DFT (TD-DFT) calculations (Figures S6–S13, Table S2). The pyridinium moiety, where the LUMO is mainly located, behaves as an electron-accepting unit, and the aryl groups attached to the nitrogen atom, where the HOMO is mainly located, behave as an electron-donating unit. The simulated transition for the weak absorption bands was assignable to the HOMO–LUMO transition. Fluorescence spectra of the cationic azatriphenylene products in MeCN solutions show a unimodal emission peak (Figure 3b). The fluorescence quantum efficiencies Φ_FL_ of **2a** (0.57) and **2d** (0.62), which bear methoxy groups, are substantially higher than those of **2i** (0.27) and **2j** (0.11). The emission wavelengths compared with that of **2i** were red-shifted by the introduction of methoxy groups (**2a** and **2d**) or the expansion of the π-system (**2j**).

Cyclic voltammetry (CV) measurements were performed for the cationic azatriphenylene derivatives in MeCN solutions (Figure 3c). The cyclic voltammogram of **2a** shows an irreversible oxidation current (*E*_1/2_^ox^ = 2.11 V vs SCE) and an irreversible reduction current (*E*_1/2_^red^ = −1.00 V vs SCE). The oxidation potential of **2a** was markedly shifted to a positive region from that of precursor **1a** (Figure S1) because the methoxyphenyl moiety was incorporated into the cationic azatriphenylene skeleton. Because of the electron-deficient nature of the cationic azatriphenylene derivative, the reduction potential was relatively positive, whereas the reduction wave did not appear in the CV of the precursor (Figure S1). The irreversible electron-transfer behavior indicates that neither the oxidized nor the reduced state of **2a** was sufficiently stable for it to undergo subsequent chemical reactions. However, the cyclic voltammogram of **2d**, which possesses two electron-donating methoxy groups, shows a reversible oxidation response at a slightly negative potential compared with that of **2a**, indicating that the oxidized state was sufficiently stable to prevent possible side reactions such as dimerization. Among the cationic azatriphenylene products, **2i**, which lacks methoxy groups, showed a higher oxidation potential and a large HOMO–LUMO energy gap. The cyclic voltammogram of π-extended **2j** shows interesting irreversible multiple-reduction behavior, presumably due to the competitive reduction of a reducible pyridinium moiety and naphthalene moiety. As summarized in Figure S5, the HOMO–LUMO gaps of **2a**–**2j** are mainly influenced by the HOMO level (i.e., the structure of the substituted benzene moiety). Because compound **2i** is an *N*^+^-doped analogue of triphenylene, we comprehensively compared its physical properties with those of triphenylene (see the Supporting Information, Figure S14 and Table S3).

Finally, single-crystal X-ray diffraction analysis of the cationic azatriphenylene derivatives was performed (Figure 4). The crystal structure of **2a** is highly planar; its torsion angle around the N atom is 4.2° (Figure 4a). In our previous study, the torsion angle of the fluorinated cationic azatriphenylene derivative was 20°. The high planarity of **2a** resulted in well-stacked pairs with a large contact area. However, **2j**, which has a naphthalene moiety, has a [4]-helicene structure with a torsion angle of 27.8° (Figure 4b) because of steric repulsion between the hydrogen atoms of the pyridinium and naphthalene moieties. The columnar stacking structure of **2j** was composed of a racemic mixture (i.e., alternating *P*-helicene and *M*-helicene). Because of the highly twisted structure of **2j**, the contact area in the stack was smaller than that of **2a**. The proposed simple cyclization method can be a straightforward way to provide helical *N*^+^-doped PAHs with chiroptoelectronic properties.^15^

**Figure 4:**
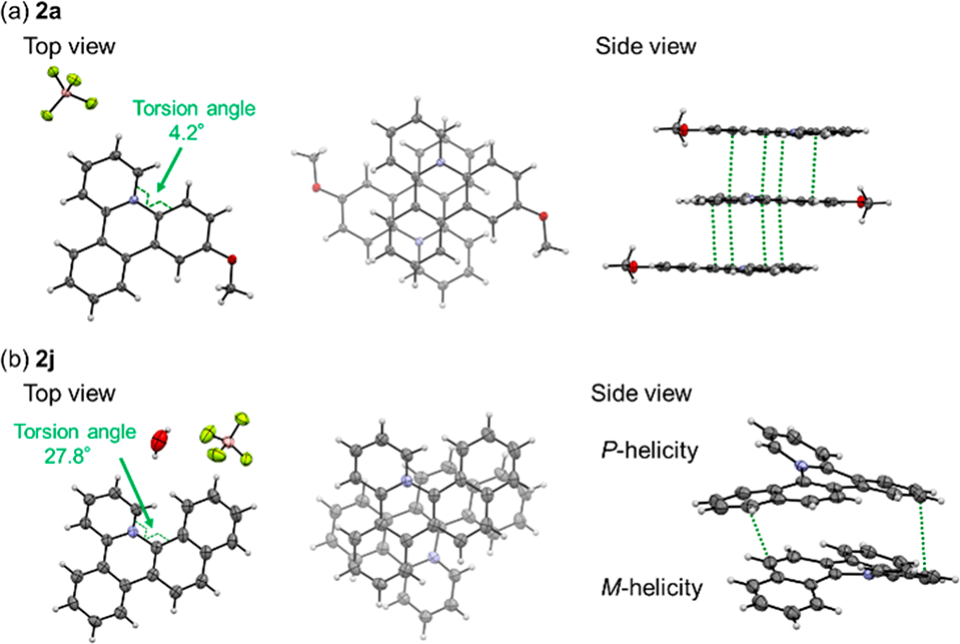
Crystal structures of (a) **2a** and (b) **2j**. Short atomic contacts of C···C are indicated by green dotted lines.

In conclusion, we have demonstrated a facile intramolecular cyclization reaction based on anodic pyridination to afford various cationic azatriphenylene derivatives. The reaction mechanism, particularly the reaction selectivity, was elucidated with the aid of DFT calculations. The cationic azatriphenylene products were found to have ICT interactions because of the donor–acceptor structure composed of an arene moiety and a pyridinium moiety. The intramolecular anodic pyridination method is useful for introducing a nitrogen cation into PAHs to perturb the π-electron system. The development of additional π-expanded *N*^+^-doped PAHs that include helicene architectures is currently underway in our lab.

## Data Availability

The data underlying this study are available in the published article and its Supporting Information.

## Supporting Information Available

Detailed experimental procedures, characterization data, DFT studies, single-crystal X-ray data, and copies of NMR spectra (PDF)
