# Supplementary material for: Synthesis of Cationic Azatriphenylene Derivatives by Electrochemical Intramolecular Pyridination and Characterization of Their Optoelectronic Properties
Source: Org Lett. 2023 May 24;25(21):3951–5. doi: 10.1021/acs.orglett.3c01341 (PMC10243103; doi:10.1021/acs.orglett.3c01341)
Supplement: Supplementary file 1 — ol3c01341_si_001.pdf [file ol3c01341_si_001.pdf]

Supporting Information

Synthesis of Cationic Azatriphenylene Derivatives by Electrochemical Intramolecular Pyridination and Characterization of Their Optoelectronic Properties

Yushi Ohno,[†] *Shogo Ando,*[†] *Daisuke Furusho,*[†] *Ryoyu Hifumi,*[†] *Yuuya Nagata,*[‡] *Ikuyoshi Tomita,*[†] *and Shinsuke Inagi*[†,*]

[†]Department of Chemical Science and Engineering, School of Materials and Chemical Technology, Tokyo Institute of Technology, 4259 Nagatsuta-cho, Midori-ku, Yokohama, Kanagawa 226-8502, Japan
[‡]Institute for Chemical Reaction Design and Discovery, Hokkaido University, Kita 21 Nishi 10, Kita-Ku, Sapporo, Hokkaido 001-0021, Japan

* Corresponding author: inagi@cap.mac.titech.ac.jp (S. I.)

Experimental

1. General considerations

All reagents and dehydrated solvents were obtained from commercial source and used without further purification unless otherwise noted. 2-(2-Bromophenyl)pyridine (**S1**) was synthesized according to the reported procedure.[1] ^{1}H, ^{13}C and ^{19}F NMR spectra were recorded on a Bruker Advance III HD500 (^{1}H: 500.13 MHz, ^{13}C: 125.76 MHz, ^{19}F: 470.59 MHz) spectrometer using $CDCl_3$, MeCN-d_3 or DMSO-d_6 as a solvent. ^{1}H NMR spectra were recorded with TMS (0.00 ppm) or a residual solvent peak (MeCN-d_3: 1.94 ppm) as internal reference; ^{13}C NMR spectra were recorded with a residual solvent peak ($CDCl_3$: 77.16 ppm, MeCN-d_3: 118.26 ppm, DMSO-d_6: 39.52 ppm) as internal reference; ^{19}F NMR spectra were recorded with C_6H_5F (−113.15 ppm). High-performance liquid chromatography (HPLC) was performed on an LC-5060 LaboACE recycling preparative HPLC system. Fourier transform infrared (FT-IR) spectra were measured on a SHIMAZU IRTracer-100 spectrometer. High-resolution mass spectra (HRMS) were obtained on a JEOL JMS-700 spectrometer. Thermogravimetric analysis (TGA) was carried out on a SHIMADZU TGA-50 analyzer under nitrogen atmosphere with a heating rate of 10 K min^{-1}. Differential scanning calorimetry (DSC) analysis was carried out on a SHIMADZU DSC-60 analyzer under nitrogen atmosphere with a heating rate of 20 K min^{-1}. Melting temperatures (T_m) are described as the range from melting onset to peak temperature observed by DSC. The cyclic voltammetry (CV) measurements were performed using an ALS/DY2325 BI-POTENTIOSTAT. All CV measurements were carried out in the three-electrode system equipped with a Pt disk working electrode (ϕ = 3 mm), a Pt plate counter electrode (10 mm × 10 mm) and a SCE reference electrode in MeCN or CH_2Cl_2 solution of Bu_4NPF_6 (0.1 M or 0.2 M) at a scan rate of 100 mVs^{-1}. UV-vis absorption spectra were recorded in MeCN or CH_2Cl_2 a SHIMADZU UV-1900i spectrophotometer. Photoluminescence (PL) spectra were obtained on a SHIMADZU RF-6000 spectrophotometer. The single crystals suitable for X-ray diffraction were obtained from a MeCN/CH_2Cl_2, MeCN/MeOH or MeCN/hexane solution at room temperature. Single crystal X-ray analysis was carried out on a Rigaku XtaLAB Synergy-DW Imaging Plate diffractometer (Cu *K*α radiation, λ = 1.54184 Å). An empirical absorption correction was carried out by the MULTI-SCAN method. The structures were solved by the SHELXT (SHELX2014) using OLEX2 software. The non-hydrogen atoms were refined anisotropically. Hydrogen atoms were refined using the riding model. All theoretical calculations were performed using Gaussian 16 software with the solvent effect of MeCN incorporated by the polarized continuum model (PCM). Geometry optimizations and frequency calculations were performed for all compounds at the B3LYP or ωB97XD level

of theory using 6-31G+(d,p) basis set for all atoms. Single point calculations were performed B3LYP level theory using 6-311G++(2df, 2p) basis set for all other atoms. Elemental analysis was performed on a J-SCIENCE MICRO CORDER JM10 for C, H and N.

2. Synthesis

2-1. Synthesis of 1a, 1b, 1d, 1e, 1g, 1h, 1i

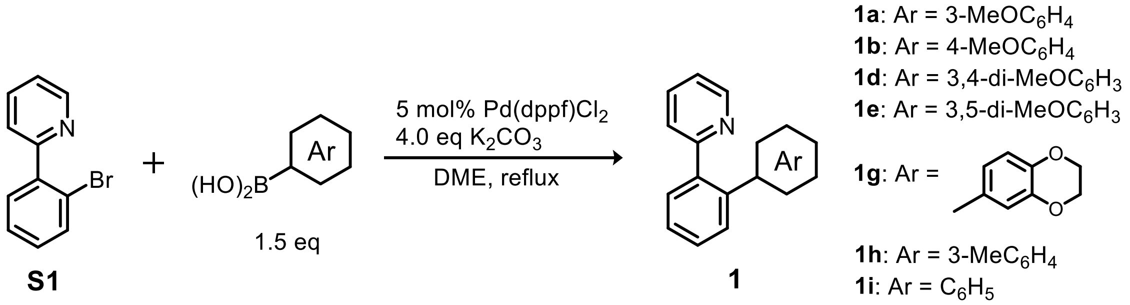

*Synthesis of 2-(3'-methoxy-[1,1'-biphenyl]-2-yl)pyridine (**1a**)*[2]

To a DME solution of 3-methoxyboronic acid (1.82 g, 12.0 mmol) and K_2CO_3 (4.43 g, 32.1 mmol) was added successively **S1** (1.92 g, 8.20 mmol) and [1,1'-bis(diphenylphosphine)ferrocene]dichloropalladium(II) (298 mg, 0.41 mmol) under an argon atmosphere, then the mixture was stirred at reflux for 23 h in an oil bath. After cooling to room temperature, the resulting mixture was extracted with ethyl acetate. The organic layer was washed with brine and dried over anhydrous Na_2SO_4. After filtration over celite and removal of the solvent, the residue was purified by silica gel column chromatography using hexane/ethyl acetate/Et_3N (10/1/0.11) as an eluent and recrystallized from hexane to give a colorless solid (1.73 g, 81%).

1a: colorless solid; ^{1}H NMR (500.13 MHz, $CDCl_3$, ppm): δ = 8.64 (d, J = 4.8 Hz, 1H), 7.71–7.69 (m, 1H), 7.49–7.44 (m, 3H), 7.42–7.38 (m, 1H), 7.16–7.10 (m, 2H), 6.92 (d, J = 7.8 Hz, 1H), 6.78–6.76 (m, 2H), 6.68 (s, 1H), 3.63 (s, 3H); ^{13}C NMR (125.76 MHz, $CDCl_3$, ppm): δ = 159.5, 159.3, 149.5, 142.8, 140.6, 139.7, 135.4, 130.6, 130.5, 129.2, 128.7, 127.9, 125.5, 122.3, 121.5, 115.1, 113.0, 55.2.

*Synthesis of 2-(4'-methoxy-[1,1'-biphenyl]-2-yl)pyridine (**1b**)*[2]

To a DME solution (40 mL) of 4-methoxyphenylboronic acid (459 mg, 3.02 mmol) and K_2CO_3 (1.10 g, 7.96 mmol) were added successively **S1** (463 mg, 1.98 mmol) and [1,1'-bis(diphenylphosphine)ferrocene]dichloropalladium(II) (77.2 mg, 0.11 mmol) under an argon atmosphere, then the mixture was stirred at reflux for 24 h in an oil bath. After cooling to room temperature, the resulting mixture was extracted with ethyl acetate. The organic layer was washed with brine and dried over anhydrous Na_2SO_4. After filtration over celite and removal of the solvent, the residue was purified by silica gel column chromatography using hexane/ethyl acetate/Et_3N (7/1/0.08) as an eluent and recrystallized from hexane to give a colorless solid (320 mg, 62%).

1b: colorless solid; ^{1}H NMR (500.13 MHz, $CDCl_3$, ppm): δ = 8.64 (d, J = 4.5 Hz, 1H), 7.68–7.66 (m, 1H), 7.46–7.38 (m, 4H), 7.12–7.06 (m, 3H), 6.90 (d, J = 8.0 Hz, 1H), 6.79–6.76 (m, 2H), 3.79 (s, 3H); ^{13}C NMR (125.76 MHz, $CDCl_3$, ppm): δ = 159.6, 158.7, 149.6, 140.3, 139.5, 135.4, 133.9, 130.9, 130.63, 130.57, 128.6, 127.4, 125.6, 121.4, 113.7, 55.3.

*Synthesis of 2-(3',4'-dimethoxy-[1,1'-biphenyl]-2-yl)pyridine (**1d**)*

To a DME solution (40 mL) of 3,4-dimethoxyphenylboronic acid (581 mg, 3.19 mmol) and K_2CO_3 (1.19 g, 8.61 mmol) was added successively **S1** (494 mg, 2.11 mmol) and [1,1'-bis(diphenylphosphine)-ferrocene]dichloropalladium(II) (80.3 mg, 0.11 mmol) under an argon atmosphere, then the mixture was stirred at reflux for 24 h in an oil bath. After cooling to room temperature, the resulting mixture was extracted with ethyl acetate. The organic layer was washed with brine and dried over anhydrous Na_2SO_4. After filtration over celite and removal of the solvent, the residue was purified by silica gel column chromatography (eluent, 1st; hexane/ethyl acetate (2/1 → 1/1), 2nd; hexane/ethyl acetate (1/1)) to give a yellow oil (320 mg, 62%).

1d: yellow oil; ^{1}H NMR (500.13 MHz, $CDCl_3$, ppm): δ = 8.65–8.64 (m, 1H), 7.68–7.66 (m, 1H), 7.46–7.44 (m. 3H), 7.43–7.39 (m, 1H), 7.11 (ddd, J = 7.5, 5.0, 1.1 Hz, 1H), 6.92 (d, J = 7.9 Hz, 1H), 6.83 (dd, J = 8.3, 1.9 Hz, 1H), 6.80 (d, J = 8.3 Hz, 1H), 6.55 (d, J = 1.7 Hz, 1H), 3.87 (s, 3H), 3.58 (s, 3H); ^{13}C NMR (125.76 MHz, MeCN-d_3, ppm): δ = 160.6, 150.2, 149.4, 149.2, 141.3, 140.7, 136.4, 134.7, 131.3, 131.1, 129.4, 128.1, 126.0, 122.6, 122.5, 114.6, 112.3, 56.2, 56.0; HRMS (ESI) calcd for $C_{19}H_{18}NO_2$ ($[M+H]^+$) m/z 292.1332, found 292.1329; IR (KBr, cm^{-1}): 1586, 1568, 1521, 1488, 1464, 1441, 1424, 1407, 1325, 1273, 1249, 1214, 1173, 1141, 1026, 772, 756.

*Synthesis of 2-(3',5'-dimethoxy-[1,1'-biphenyl]-2-yl)pyridine (**1e**)*[3]

To DME solution (40 mL) of 3,5-dimethoxyphenylboronic acid (587 mg, 3.23 mmol) and K_2CO_3 (1.18 g, 8.54 mmol) were added successively **S1** (502 mg, 2.14 mmol) and [1,1'-bis(diphenylphosphine)-ferrocene]dichloropalladium(II) (79.1 mg, 0.11 mmol) under an argon atmosphere, then the mixture was stirred at reflux for 24 h in an oil bath. After cooling to room temperature, the resulting mixture was extracted with ethyl acetate. The organic layer was washed with brine and dried over anhydrous Na_2SO_4. After filtration over celite and removal of the solvent, the residue was purified by silica gel column chromatography (eluent, 1st; hexane/ethyl acetate (3/1 → 2/1), 2nd; hexane/ethyl acetate (2/1)) to give a colorless solid (378 mg, 61%).

1e: colorless solid; ^{1}H NMR (500.13 MHz, $CDCl_3$, ppm): δ = 8.65–8.64 (m, 1H), 7.70–7.68 (m, 1H), 7.49–7.45 (m, 3H), 7.45–7.40 (m, 1H), 7.12 (ddd, J = 7.5, 4.9, 1.1 Hz, 1H),

6.97 (d, J = 8.0 Hz, 1H), 6.34–6.31 (m, 3H), 3.62 (s, 6H); ^{13}C NMR (125.76 MHz, MeCN-d_3, ppm): δ = 161.4, 160.4, 150.1, 144.3, 141.4, 140.8, 136.4, 131.3, 131.1, 129.4, 128.5, 125.8, 122.6, 108.7, 99.7, 55.8.

*Synthesis of 2-(2-(2,3-dihydrobenzo[b][1,4]dioxin-6-yl)phenyl)pyridine (**1g**)*

To a DME solution (30 mL) of 1,4-benzodioxane-6-boronic acid (406 mg, 2.26 mmol) and K_2CO_3 (830 mg, 6.01 mmol) was added successively **S1** (355 mg, 1.52 mmol) and [1,1'-bis(diphenylphosphine)ferrocene]dichloropalladium(II) (60.2 mg, 0.08 mmol) under an argon atmosphere, then the mixture was stirred at reflux for 24 h in an oil bath. After cooling to room temperature, the resulting mixture was extracted with ethyl acetate. The organic layer was washed with brine and dried over anhydrous Na_2SO_4. After filtration over celite and removal of the solvent, the residue was purified by silica gel column chromatography (eluent, 1st; hexane/ethyl acetate (12/1 → 5/1), 2nd; hexane/toluene/ethyl acetate (12/0.3/0.7)) to give a colorless solid (228 mg, 52%).

1g: colorless solid; T_m = 87.5–91.7 °C; ^{1}H NMR (500.13 MHz, $CDCl_3$, ppm): δ = 8.64 (d, J = 4.8 Hz, 1H), 7.68–7.65 (m, 1H), 7.45–7.38 (m, 4H), 7.11 (dd, J = 7.5, 5.0 Hz, 1H), 6.96 (d, J = 8.0 Hz, 1H), 6.74 (d, J = 2.2 Hz, 1H), 6.69 (d, J = 8.4 Hz, 1H), 6.54 (dd, J = 8.4, 2.2 Hz, 1H), 4.26–4.22 (m, 4H); ^{13}C NMR (125.76 MHz, $CDCl_3$, ppm): δ = 159.5, 149.6, 143.3, 142.7, 140.1, 139.5, 135.4, 134.9, 130.63, 130.56, 128.6, 127.5, 125.5, 123.3, 121.5, 118.5, 116.9, 64.52, 64.46; HRMS (ESI) calcd for $C_{19}H_{16}NO_2$ ($[M+H]^+$) m/z 290.1176, found 290.1176; IR (KBr, cm^{-1}): 1586, 1511, 1488, 1464, 1316, 1291, 1285, 1249, 1225, 1071, 1065, 896, 888, 824, 752.

*Synthesis of 2-(3'-methyl-[1,1'-biphenyl]-2-yl)pyridine (**1h**)*[4]

To a DME solution (30 mL) of 3-methylphenylboronic acid (306 mg, 2.25 mmol) and K_2CO_3 (831 mg, 6.01 mmol) was added successively **S1** (353 mg, 1.51 mmol) and [1,1'-bis(diphenylphosphine)ferrocene]dichloropalladium(II) (62.8 mg, 0.09 mmol) under an argon atmosphere, then the mixture was stirred at reflux for 24 h in an oil bath. After cooling to room temperature, the resulting mixture was extracted with ethyl acetate. The organic layer was washed with brine and dried over anhydrous Na_2SO_4. After filtration over celite and removal of the solvent, the residue was purified by silica gel column chromatography using hexane/ethyl acetate/Et_3N (25/1/1.3) as an eluent to give a colorless solid (325 mg, 88%).

1h: colorless solid; ^{1}H NMR (500.13 MHz, $CDCl_3$, ppm): δ = 8.64–8.62 (m, 1H), 7.70–7.67 (m, 1H), 7.47–7.42 (m, 3H), 7.41–7.37 (m, 1H), 7.11–7.08 (m, 2H), 7.04–7.01 (m, 2H), 6.92–6.89 (m, 2H), 2.26 (s, 3H); ^{13}C NMR (125.76 MHz, $CDCl_3$, ppm): δ = 159.5,

149.5, 141.4, 140.9, 139.6, 137.8, 135.3, 130.6, 130.6, 128.6, 128.0, 127.7, 127.6, 127.0, 125.5, 121.4, 21.5.

*Synthesis of 2-([1,1'-biphenyl]-2-yl)pyridine (**1i**)*[2]

To a DME solution (30 mL) of phenylboronic acid (274 mg, 2.25 mmol) and K_2CO_3 (830 mg, 6.01 mmol) was added successively **S1** (349 mg, 1.49 mmol) and [1,1'-bis(diphenylphosphine)ferrocene]dichloropalladium(II) (60.2 mg, 0.082 mmol) under an argon atmosphere, then the mixture was stirred at reflux for 21 h in an oil bath. After cooling to room temperature, the resulting mixture was extracted with ethyl acetate. The organic layer was washed with brine and dried over anhydrous Na_2SO_4. After filtration over celite and removal of the solvent, the residue was purified by silica gel column chromatography using hexane/ethyl acetate/Et_3N (25/1/1.3) as an eluent to give a colorless solid (322 mg, 88%).

1i: colorless solid; ^{1}H NMR (500.13 MHz, $CDCl_3$, ppm): δ = 8.64–8.63 (m, 1H), 7.72–7.68 (m, 1H), 7.49–7.42 (m, 3H), 7.40–7.36 (m, 1H), 7.25–7.22 (m, 3H), 7.17–7.14 (m, 2H), 7.11–7.08 (m, 1H), 6.88 (d, J = 8.0 Hz, 1H); ^{13}C NMR (125.76 MHz, $CDCl_3$, ppm): δ = 159.4, 149.6, 141.5, 140.8, 139.6, 135.3, 130.63, 130.61, 129.9, 128.6, 128.2, 127.8, 126.8, 125.5, 121.5.

2-2. Synthesis of 1c, 1f, 1j

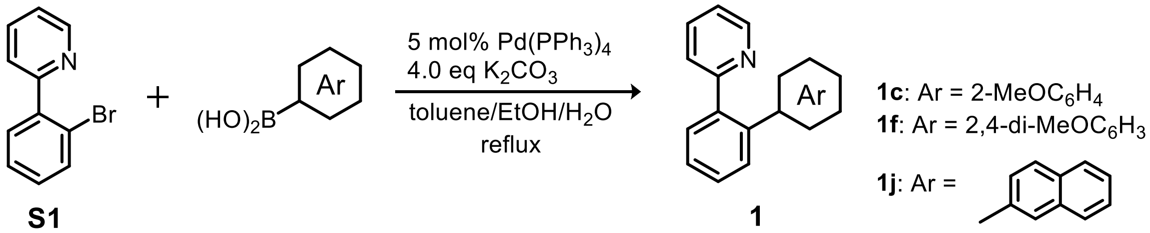

*Synthesis of 2-(2'-methoxy-[1,1'-biphenyl]-2-yl)pyridine (**1c**)*[4]

To a mixture of toluene (20 mL), ethanol bubbled with argon (5 mL), and distilled water bubbled with an argon (5 mL) of 2-methoxyphenylboronic acid (608 mg, 4.00 mmol) and K_2CO_3 (1.10 g, 7.96 mmol) was added successively **S1** (470 mg, 2.01 mmol) and tetrakis-(triphenylphosphine)palladium(0) (118 mg, 0.10 mmol) under an argon atmosphere, then the mixture was stirred at reflux for 50 h in an oil bath. After cooling to room temperature, the resulting mixture was extracted with ethyl acetate. The organic layer was washed with brine and dried over anhydrous Na_2SO_4. After filtration over celite and removal of the solvent, the residue was purified by silica gel column chromatography using hexane/ethyl

acetate/Et_3N (5/1/0.06) as an eluent and recrystallized from hexane to give a colorless solid (111 mg, 21%).

1c: colorless solid; ^1H NMR (500.13 MHz, $CDCl_3$, ppm): δ = 8.62–8.60 (m, 1H), 7.75–7.71 (m, 1H), 7.49–7.43 (m, 2H), 7.40–7.38 (m, 1H), 7.37–7.32 (m, 1H), 7.24–7.20 (m, 2H), 7.05 (ddd, *J* = 7.5, 4.9, 1.1 Hz, 1H), 6.96–6.90 (m, 2H), 6.72 (d, *J* = 8.1 Hz, 1H), 3.35 (s, 3H); ^{13}C NMR (125.76 MHz, $CDCl_3$, ppm): δ = 159.8, 156.2, 149.3, 140.5, 137.1, 135.1, 131.6, 131.1, 130.6, 129.8, 128.8, 128.4, 127.8, 123.7, 121.2, 120.8, 110.8, 55.0.

*Synthesis of 2-(2',4'-dimethoxy-[1,1'-biphenyl]-2-yl)pyridine (**1f**)*[3]

To a mixture of toluene (30 mL), ethanol bubbled with argon (7.5 mL), and distilled water bubbled with argon (7.5 mL) of 2,4-dimethoxyphenylboronic acid (819 mg, 4.50 mmol) and K_2CO_3 (1.66 g, 12.0 mmol) was added successively **S1** (705 mg, 3.01 mmol) and tetrakis-(triphenylphosphine)palladium(0) (189 mg, 0.16 mmol) under an argon atmosphere, then the mixture was stirred at reflux for 49 h in an oil bath. After cooling to room temperature, the resulting mixture was extracted with ethyl acetate. The organic layer was washed with brine and dried over anhydrous Na_2SO_4. After filtration over celite and removal of the solvent, the residue was purified by silica gel column chromatography (eluent, 1st; hexane/ethyl acetate/Et_3N (5/1/0.06), 2nd; hexane/ethyl acetate/Et_3N (5/1/0.06)) to give a yellow oil (278 mg, 32%).

1f: yellow oil; ^1H NMR (500.13 MHz, $CDCl_3$, ppm): δ = 8.62 (d, *J* = 4.3 Hz, 1H), 7.74–7.71 (m, 1H), 7.46–7.41 (m, 2H), 7.38–7.35 (m, 2H), 7.12 (d, *J* = 8.3 Hz, 1H), 7.07–7.04 (m, 1H), 6.92 (d, *J* = 7.9 Hz, 1H), 6.48 (dd, *J* = 8.3, 2.5 Hz, 1H), 6.29 (d, *J* = 2.3 Hz, 1H), 3.81 (s, 3H), 3.32 (s, 3H); ^{13}C NMR (125.76 MHz, $CDCl_3$, ppm): δ = 160.5, 160.0, 157.2, 149.3, 140.6, 136.9, 135.1, 132.0, 131.3, 129.8, 128.4, 127.6, 123.7, 123.4, 121.1, 104.5, 98.7, 55.5, 55.0.

*Synthesis of 2-(2-(naphthalen-2-yl)phenyl)pyridine (**1j**)*[5]

To a mixture of toluene (15 mL) and ethanol bubbled with argon (5 mL) of 2-naphthaleneboronic acid (645 mg, 3.75 mmol) was added successively **S1** (585 mg, 2.50 mmol), 2 M K_2CO_3 aq (5 mL) and tetrakis-(triphenylphosphine)palladium(0) (150 mg, 0.13 mmol) under an argon atmosphere, then the mixture was stirred at reflux for 24 h in an oil bath. After cooling to room temperature, the resulting mixture was extracted with ethyl acetate. The organic layer was washed with brine and dried over anhydrous Na_2SO_4. After filtration over celite and removal of the solvent, the residue was purified by silica gel column chromatography (eluent, 1st; toluene/ethyl acetate (20/1), 2nd; hexane/ethyl

acetate (20/1 → 10/1 → 5/1 →3/1)) and HPLC using $CHCl_3$ as an eluent to give a yellow oil (525 mg, 75%).

1j: yellow oil; ^1H NMR (500.13 MHz, $CDCl_3$, ppm): δ = 8.64–8.62 (m, 1H), 7.80–7.73 (m, 4H), 7.64 (d, J = 8.4 Hz, 1H), 7.57–7.53 (m, 1H), 7.53–7.48 (m, 2H), 7.47–7.43 (m, 2H), 7.33–7.28 (m, 1H), 7.16 (dd, J = 8.4, 1.7, 1H), 7.07 (ddd, J = 7.4, 4.9, 1.0 Hz, 1H), 6.91 (d, J = 7.9 Hz, 1H); ^{13}C NMR (125.76 MHz, $CDCl_3$, ppm): δ = 159.3, 149.6, 140.6, 139.8, 139.2, 135.5, 133.5, 132.3, 131.0, 130.7, 128.8, 128.42, 128.36, 128.2, 127.9, 127.7, 127.5, 126.2, 126.0, 125.6, 121.5.

2-2. General procedure for anodic oxidation of 1

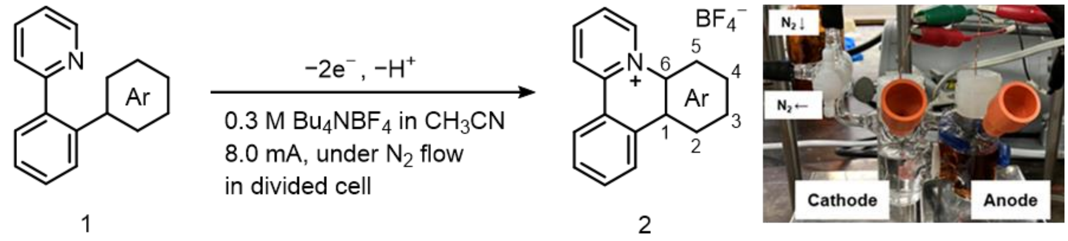

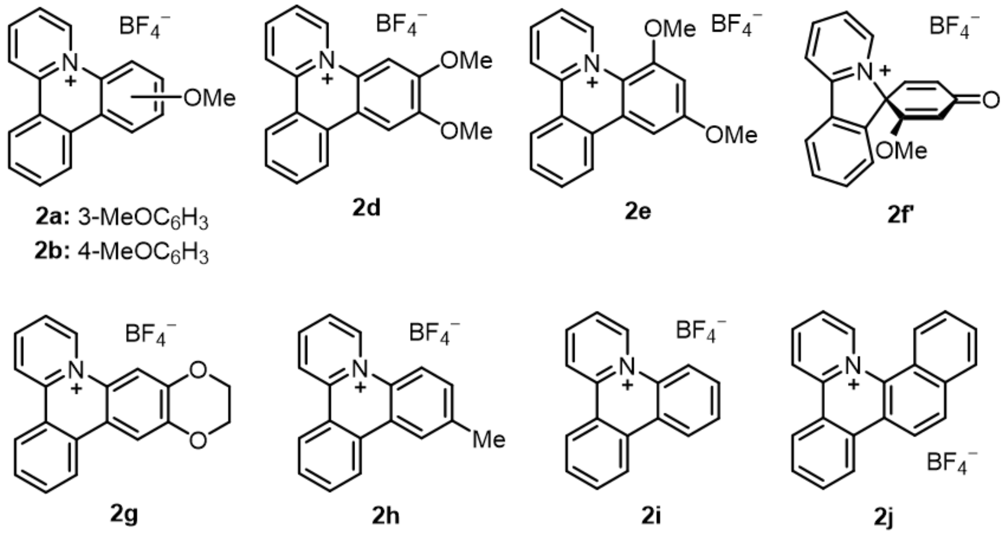

The anodic oxidation was performed in a divided cell (4G glass filter) equipped with a carbon felt (Nilaco C-074611, ca. 160 mg, dried at 150 °C for 5 h before use) and a Pt plate cathode (1 cm × 1 cm) under nitrogen flow. **1** (0.40 mmol) and 0.3 M Bu_4NBF_4/MeCN bubbled with argon (20 mL) were added to the anodic compartment. In the cathodic compartment was added trifluoromethanesulfonic acid (400 µL) in 0.3 M Bu_4NBF_4/MeCN bubbled with argon (20 mL). After X F mol^{-1} of electricity was

consumed under constant current (8.0 mA), the resulting mixture in the anodic compartment was collected by rinsing with MeCN and was evaporated under reduced pressure. The residue was washed with refrigerated MeOH or distilled water several times to give desired product **2**.

*2-Methoxypyrido[1,2-f]phenanthridin-5-ium tetrafluoroborate (**2a**)*

After the electrochemical reaction (2.6 F/mol) of **1a**, the residue was washed with refrigerated MeOH to give a tawny solid (84 mg, 60%).

2a: tawny solid; T_m = 294.0–298.7 °C; ^{1}H NMR (500.13 MHz, MeCN-d_3, ppm): δ = 9.84 (d, J = 6.9 Hz, 1H), 9.23 (d, J = 8.6 Hz, 1H), 8.87 (d, J = 8.3 Hz, 1H), 8.84 (d, J = 8.4 Hz, 1H), 8.63 (d, J = 9.7 Hz, 1H), 8.59 (m, 1H), 8.21 (d, J = 2.9 Hz, 1H), 8.17–8.11 (m, 2H), 8.02–7.98 (m, 1H), 7.61 (dd, J = 9.6, 2.9 Hz, 1H), 4.11 (s, 3H); ^{13}C NMR (125.76 MHz, MeCN-d_3, ppm): δ = 162.3, 144.3, 142.2, 136.4, 135.3, 131.6, 130.3, 127.9, 127.5, 127.3, 125.9, 125.0, 124.8, 124.1, 121.6, 120.7, 107.7, 57.1; ^{19}F NMR (470.59 MHz, MeCN-d_3, ppm): δ = −150.0 (d, J = 26.0 Hz, 4F); HRMS (ESI) calcd for $C_{18}H_{14}NO$ ($[M]^+$) m/z 260.1070, found 260.1074, calcd for BF_4 ($[M]^-$) m/z 87.0035, found 87.0177; IR (KBr, cm^{-1}): 1631, 1611, 1512, 1477, 1436, 1240, 1161, 1117, 1097, 1059, 1052, 1038, 1018, 999, 760; elemental analysis (%): Anal. Calcd for $C_{18}H_{14}BF_4NO$: C, 62.28; H, 4.07; N, 4.04. Found: C, 62.01; H, 4.08; N, 4.13.

*3-Methoxypyrido[1,2-f]phenanthridin-5-ium tetrafluoroborate (**2b**)*

After the electrochemical reaction (2.0 F/mol) of **1b**, the residue was washed with refrigerated MeOH and recrystallized from MeOH to give a light yellow solid (19 mg, 14%).

2b: light yellow solid; T_m = 258.6–262.2 °C; ^{1}H NMR (500.13 MHz, MeCN-d_3, ppm): δ = 9.91 (d, J = 7.0 Hz, 1H), 9.27 (d, J = 8.7 Hz, 1H), 8.85 (d, J = 8.4 Hz, 1H), 8.81 (d, J = 9.0 Hz, 1H), 8.72 (d, J = 8.3 Hz, 1H), 8.67–8.63 (m, 1H), 8.20–8.16 (m, 1H), 8.12–8.06 (m, 2H), 7.94–7.90 (m, 1H), 7.66 (dd, J = 9.2, 1.9 Hz, 1H), 4.11 (s, 3H); ^{13}C NMR (125.76 MHz, MeCN-d_3, ppm): δ = 163.0, 145.8, 143.1, 137.0, 135.5, 134.8, 130.9, 130.3, 127.6, 127.2, 125.5, 125.1, 123.8, 122.8, 120.5, 119.5, 103.1, 57.3; ^{19}F NMR (470.59 MHz, MeCN-d_3, ppm): δ = −150.0 (d, J = 26.0 Hz, 4F); HRMS (ESI) calcd for $C_{18}H_{14}NO$ ($[M]^+$) m/z 260.1070, found 260.1071, calcd for BF_4 ($[M]^-$) m/z 87.0035, found 87.0107; IR (KBr, cm^{-1}): 1623, 1519, 1477, 1433, 1362, 1240, 1117, 1084, 1061, 1056, 1017, 758, 718; elemental analysis (%): Anal. Calcd for $C_{18}H_{14}BF_4NO$: C, 62.28; H, 4.07; N, 4.04. Found: C, 61.98; H, 4.05; N, 4.01.

*2,3-Dimethoxypyrido[1,2-f]phenanthridin-5-ium tetrafluoroborate (**2d**)*

After the electrochemical reaction (2.0 F/mol) of **1d**, the residue was washed with refrigerated MeOH to give a yellow solid (96.7 mg, 66%).

2d: yellow solid; T_m = 299.4–304.0 °C, ^{1}H NMR (500.13 MHz, MeCN-d_3, ppm): δ = 9.88 (d, *J* = 7.0 Hz, 1H), 9.26 (dd, *J* = 8.7, 1.2 Hz, 1H), 8.87 (dd, *J* = 8.4, 0.5 Hz, 1H), 8.80 (dd, *J* = 8.3, 0.5 Hz, 1H), 8.58–8.54 (m, 1H), 8.17–8.10 (m, 3H), 7.98 (s, 1H), 7.97–7.93 (m, 1H), 4.16 (s, 3H), 4.16 (s, 3H); ^{13}C NMR (125.76 MHz, MeCN-d_3, ppm): δ = 153.6, 153.1, 144.2, 141.1, 136.2, 135.1, 130.43, 130.36, 128.0, 127.1, 125.4, 124.9, 124.1, 123.0, 120.5, 105.6, 101.4, 57.6, 57.3; ^{19}F NMR (470.59 MHz, MeCN-d_3, ppm): δ = −150.0 (d, *J* = 24.3 Hz, 4F); HRMS (ESI) calcd for $C_{19}H_{16}NO_2$ ($[M]^+$) m/z 290.1176, found 290.1180, calcd for BF_4 ($[M]^-$) m/z 87.0035, found 87.0130; IR (KBr, cm^{-1}): 1628, 1617, 1522, 1485, 1433, 1381, 1288, 1252, 1223, 1175, 1123, 1084, 1063, 1058, 1045, 1017.

*2,4-Dimethoxypyrido[1,2-f]phenanthridin-5-ium tetrafluoroborate (**2e**)*

After the electrochemical reaction (2.2 F/mol) of **1e**, the residue was washed with refrigerated MeOH to give a tawny solid (99 mg, 66%).

2e: tawny solid; T_m = 241.7–245.6 °C, ^{1}H NMR (500.13 MHz, MeCN-d_3, ppm): δ = 10.36 (d, *J* = 6.9 Hz, 1H), 9.12 (d, *J* = 8.6 Hz, 1H), 8.77 (d, *J* = 8.4 Hz, 1H), 8.74 (d, *J* = 8.2 Hz, 1H), 8.52–8.48 (m, 1H), 8.09–8.05 (m, 1H), 8.01–7.93 (m, 2H), 7.78–7.76 (m, 1H), 7.14 (d, *J* = 2.5 Hz, 1H), 4.14 (s, 3H), 4.10 (s, 3H); ^{13}C NMR (125.76 MHz, DMSO-d_6, ppm): δ = 161.1, 153.2, 143.3, 141.7, 141.0, 134.0, 130.5, 129.2, 128.6, 126.2, 124.4, 123.6, 123.4, 123.1, 117.6, 102.5, 98.8, 57.6, 56.4; ^{19}F NMR (470.59 MHz, MeCN-d_3, ppm): δ = −150.1 (d, *J* = 24.3 Hz, 4F); HRMS (ESI) calcd for $C_{19}H_{16}NO_2$ ($[M]^+$) m/z 290.1176, found 290.1182, calcd for BF_4 ($[M]^-$) m/z 87.0035, found 87.0127; IR (KBr, cm^{-1}): 1628, 1605, 1503, 1467, 1371, 1220, 1125, 1111, 1084, 1069, 1038, 1015 768; elemental analysis (%): Anal. Calcd for $C_{19}H_{16}BF_4NO_2$: C, 60.51; H, 4.28; N, 3.71. Found: C, 60.42; H, 4.32; N, 3.68.

*2-Methoxy-4-oxospiro[cyclohexane-1,6'-pyrido[2,1-a]isoindole]-2,5-dien-5'-ium tetrafluoroborate (**2f'**)*

After the electrochemical reaction (2.2 F/mol) of **1f**, the residue was washed with refrigerated MeOH to give an ochre solid (48 mg, 32%).

2f': ochre solid; T_d (at 5% weight loss) = 258.2 °C, ^{1}H NMR (500.13 MHz, MeCN-d_3, ppm): δ = 8.72–8.67 (m, 2H), 8.60–8.58 (m, 1H), 8.34–8.30 (m, 1H), 8.98–7.94 (m, 1H), 7.85–7.81 (m, 2H), 7.62–7.57 (m, 1H), 6.65 (dd, *J* = 9.8, 1.4 Hz, 1H), 6.55 (d, *J* = 9.8 Hz,

1H), 6.06 (d, J = 1.3 Hz, 1H), 3.52 (s, 3H); ^{13}C NMR (125.76 MHz, MeCN-d$_3$, ppm): δ = 186.2, 166.2 , 155.1, 149.2, 141.3, 140.9, 136.7, 135.7, 133.3, 132.7, 132.2, 127.4, 125.4, 124.3, 122.7, 106.7, 77.7, 58.0; ^{19}F NMR (470.59 MHz, MeCN-d$_3$, ppm): δ = −150.0 (d, J = 24.3 Hz, 4F); HRMS (ESI) calcd for $C_{18}H_{14}NO_2$ ([M]$^+$) m/z 276.1019, found 276.1025, calcd for BF_4 ([M]$^-$) m/z 87.0035, found 87.0126; IR (KBr, cm^{-1}): 1669, 1627, 1614, 1505, 1230, 1110, 1084, 1059, 1042.

*12,13-Dihydro-[1,4]dioxino[2,3-b]pyrido[1,2-f]phenanthridin-9-ium tetra-fluoroborate **(2g)***

After the electrochemical reaction (2.3 F/mol) of **1g**, the residue was washed with refrigerated MeOH to give a tawny solid (84 mg, 56%).

2g: tawny solid; T_m = 312.7–317.5 °C, ^{1}H NMR (500.13 MHz, MeCN-d$_3$, ppm): δ = 9.73 (d, J = 6.9 Hz, 1H), 9.22 (dd, J = 8.7, 1.2 Hz, 1H), 8.83 (d, J = 8.4 Hz, 1H), 8.66 (d, J = 8.4 Hz, 1H), 8.59–8.54 (m, 1H), 8.28 (s, 1H), 8.19 (s, 1H), 8.14–8.10 (m, 1H), 8.10–8.05 (m, 1H), 7.94–7.90 (m, 1H), 4.51 (s, 4H); ^{13}C NMR (125.76 MHz, DMSO-d$_6$, ppm): δ = 146.6, 146.4, 143.2, 141.1, 136.4, 134.1, 129.3, 129.2, 127.4, 126.6, 124.7, 124.1, 123.4, 122.1, 119.6, 111.4, 107.2, 64.7, 64.6; ^{19}F NMR (470.59 MHz, MeCN-d$_3$, ppm): δ = −150.1 (d, J = 24.3 Hz, 4F); HRMS (ESI) calcd for $C_{19}H_{14}NO_2$ ([M]$^+$) m/z 288.1019, found 288.1022, calcd for BF_4 ([M]$^-$) m/z 87.0035, found 87.0158; IR (KBr, cm^{-1}): 1629, 1607, 1592, 1555, 1521, 1475, 1443, 1334, 1315, 1250, 1173, 1125, 1105, 1084, 1075, 1064, 1038.

*2-Methylpyrido[1,2-f]phenanthridin-5-ium tetrafluoroborate **(2h)***

After the electrochemical reaction (3.2 F/mol) of **1h**, the residue was washed with refrigerated MeOH to give a light grey solid (15 mg, 11%).

2h: light grey solid; T_d (at 5% weight loss) = 328.1 °C, ^{1}H NMR (500.13 MHz, MeCN-d$_3$, ppm): δ = 9.91 (d, J = 7.0 Hz, 1H), 9.26 (d, J = 7.9 Hz, 1H), 8.88 (d, J = 8.4 Hz, 1H), 8.83 (d, J = 8.3 Hz, 1H), 8.71 (s, 1H), 8.66–8.62 (m, 1H), 8.59 (d, J = 9.0 Hz, 1H), 8.20–8.16 (m, 1H), 8.16–8.11 (m, 1H), 8.01–7.97 (m, 1H), 7.87 (dd, J = 9.0, 1.9 Hz, 1H), 2.69 (s, 3H); ^{13}C NMR (125.76 MHz, DMSO-d$_6$, ppm): δ = 143.6, 142.1, 141.3, 136.5, 134.2, 132.3, 130.7, 130.2, 129.2, 126.7, 124.9, 124.6, 124.5, 124.1, 123.6, 123.0, 119.0, 20.9; ^{19}F NMR (470.59 MHz, MeCN-d$_3$, ppm): δ = −150.0 (d, J = 26.0 Hz, 4F); HRMS (ESI) calcd for $C_{18}H_{14}N$ ([M]$^+$) m/z 244.1121, found 244.1125, calcd for BF_4 ([M]$^-$) m/z 87.0035, found 87.0064; IR (KBr, cm^{-1}): 1629, 1608, 1474, 1439, 1124, 1105, 1084, 1071, 1064, 1055, 1038, 761, 715.

*Pyrido[1,2-f]phenanthridin-5-ium tetrafluoroborate (**2i**)*[6]

After the electrochemical reaction (2.5 F/mol) of **1i**, the residue was washed with refrigerated MeOH to give a tawny solid (45 mg, 35%).

2i: tawny solid; T_d (at 5% weight loss) = 358.7 °C, ^{1}H NMR (500.13 MHz, MeCN-d_3, ppm): δ = 9.96 (d, *J* = 7.0 Hz, 1H), 9.30 (dd, *J* = 8.7, 1.1 Hz, 1H), 8.94–8.89 (m, 2H), 8.85 (d, *J* = 8.3 Hz, 1H), 8.74–8.66 (m, 2H), 8.23–8.19 (m, 1H), 8.18–8.14 (m, 1H), 8.08–8.04 (m, 2H), 8.03–7.99 (m, 1H); ^{13}C NMR (125.76 MHz, DMSO-d_6, ppm): δ = 144.1, 142.7, 136.8, 134.3, 132.7, 131.2, 130.9, 130.3, 129.3, 126.7, 125.0, 124.9, 124.6, 124.1, 123.6, 123.0, 119.3; ^{19}F NMR (470.59 MHz, MeCN-d_3, ppm): δ = −150.0 (d, *J* = 26.0 Hz, 4F); HRMS (ESI) calcd for $C_{17}H_{12}N$ ($[M]^+$) m/z 230.0964, found 230.0965, calcd for BF_4 ($[M]^-$) m/z 87.0035, found 87.0157; IR (KBr, cm^{-1}): 1632, 1510, 1474, 1441, 1436, 1361, 1084, 1066, 1054, 1039, 1017, 997, 755, 522; elemental analysis (%): Anal. Calcd for $C_{17}H_{12}BF_4N$: C, 64.39; H, 3.81; N, 4.42. Found: C, 64.00; H, 4.00; N, 4.61.

*Benzo[c]pyrido[1,2-f]phenanthridin-11-ium tetrafluoroborate (**2j**)*

After the electrochemical reaction (3.0 F/mol) of **1j** in 0.3 M Et_4NBF_4/MeCN, the residue was washed with refrigerated distilled water to give a brown solid (50 mg, 34%).

2j: brown solid; T_d (at 5% weight loss) = 268.1 °C, ^{1}H NMR (500.13 MHz, MeCN-d_3, ppm): δ = 10.01 (d, *J* = 6.9 Hz, 1H), 9.27 (dd, *J* = 8.7, 1.1 Hz, 1H), 8.92 (d, *J* = 8.4 Hz, 1H), 8.86 (d, *J* = 8.3 Hz, 1H), 8.77–8.73 (m, 2H), 8.67–8.62 (m, 1H), 8.44 (d, *J* = 8.9 Hz, 1H), 8.29–8.24 (m, 1H), 8.22–8.17 (m, 1H), 8.11–8.03 (m, 2H), 7.87–7.81 (m, 2H); ^{13}C NMR (125.76 MHz, DMSO-d_6, ppm): δ = 144.4, 142.3, 142.1, 134.5, 134.1, 131.9, 130.7, 129.9, 129.4, 128.9, 128.8, 128.6, 126.2, 124.4, 124.2, 124.1, 123.83, 123.81, 123.3, 122.9, 119.9; ^{19}F NMR (470.59 MHz, MeCN-d_3, ppm): δ = −150.1 (d, *J* = 24.3 Hz, 4F); HRMS (ESI) calcd for $C_{21}H_{14}N$ ($[M]^+$) m/z 280.1121, found 280.1127, calcd for BF_4 ($[M]^-$) m/z 87.0035, found 87.0068; IR (KBr, cm^{-1}): 1626, 1608, 1499, 1463, 1421, 1221, 1218, 1163, 1137, 1125, 1084, 1063, 1033, 767.

3. Electrochemical properties of 1a

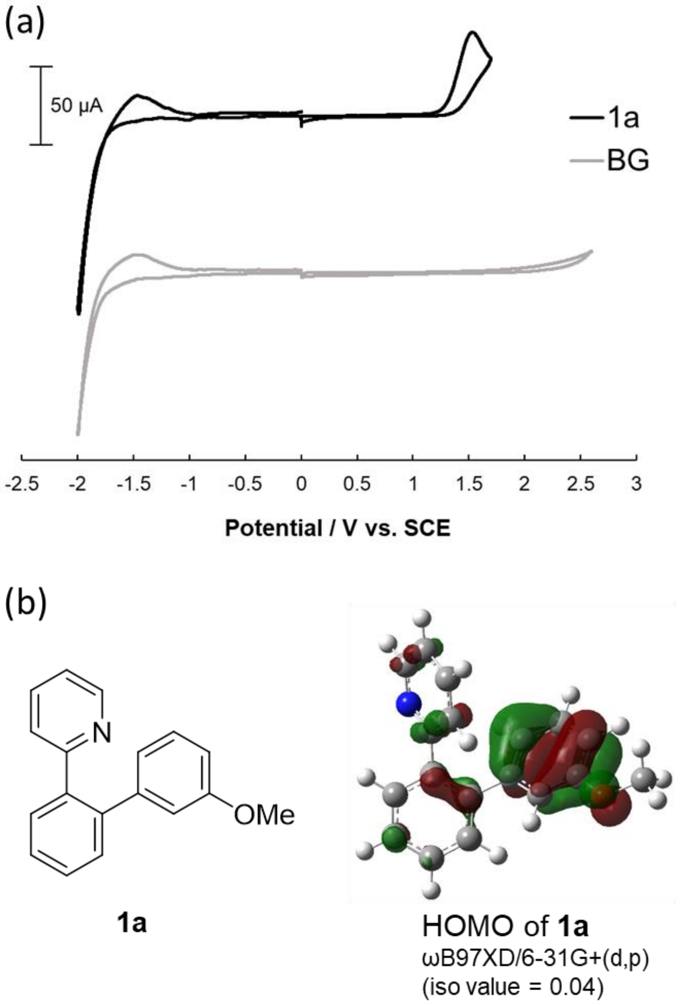

Figure S1. (a) Cyclic voltammograms of 3 mM **1a** in 0.1 M Bu_4NPF_6/MeCN at a scan rate of 100 mV/s. (b) The highest occupied molecular orbital (HOMO) diagram of **1a**.

4. The reactivity and regioselectivity for cyclization

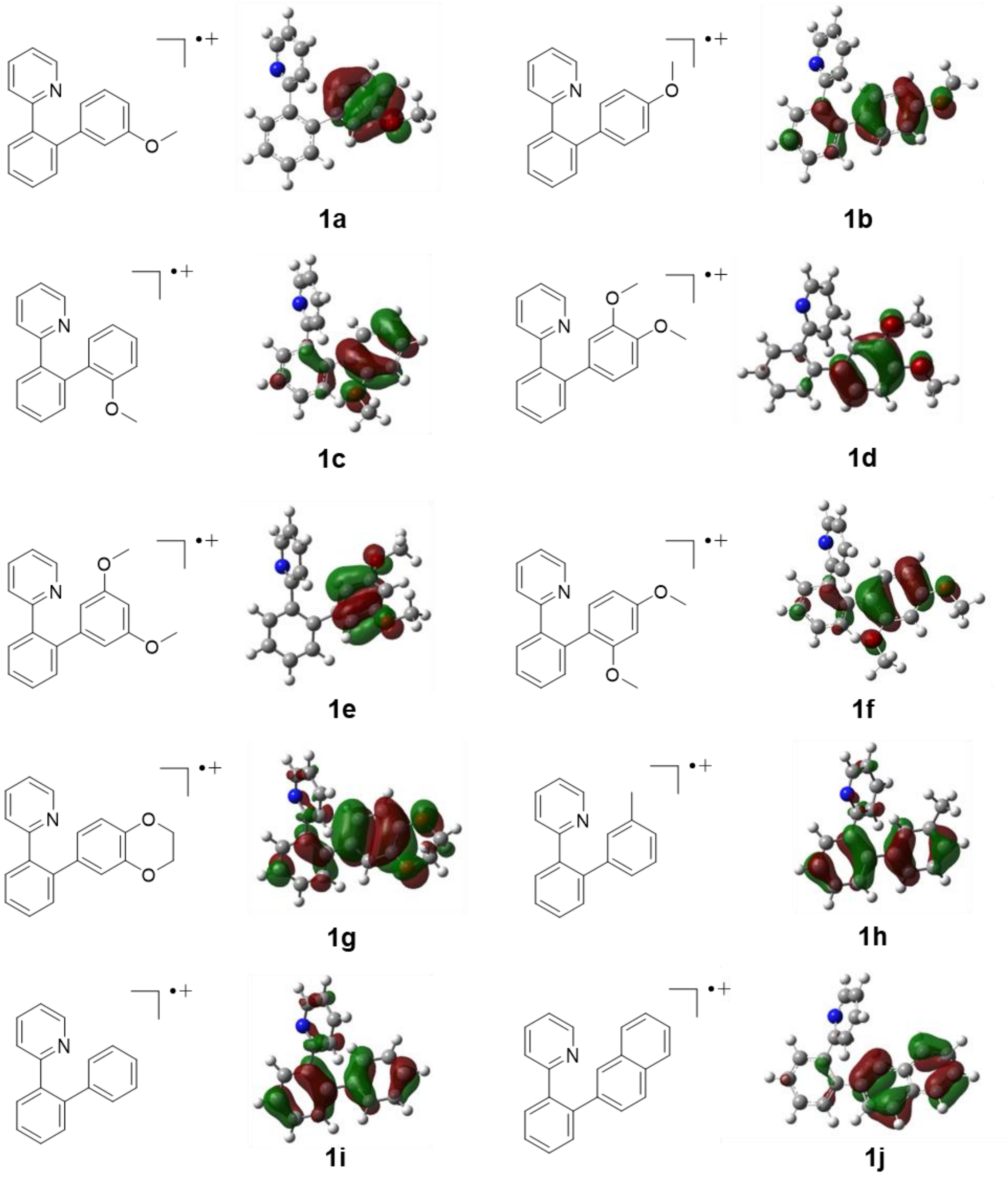

Figure S2. The lowest unoccupied molecular orbitals (β-LUMO) of **1a**-**1j** in one-electron oxidation state calculated at the UωB97XD/6-31G+(d, p) level basis set (iso value = 0.04).

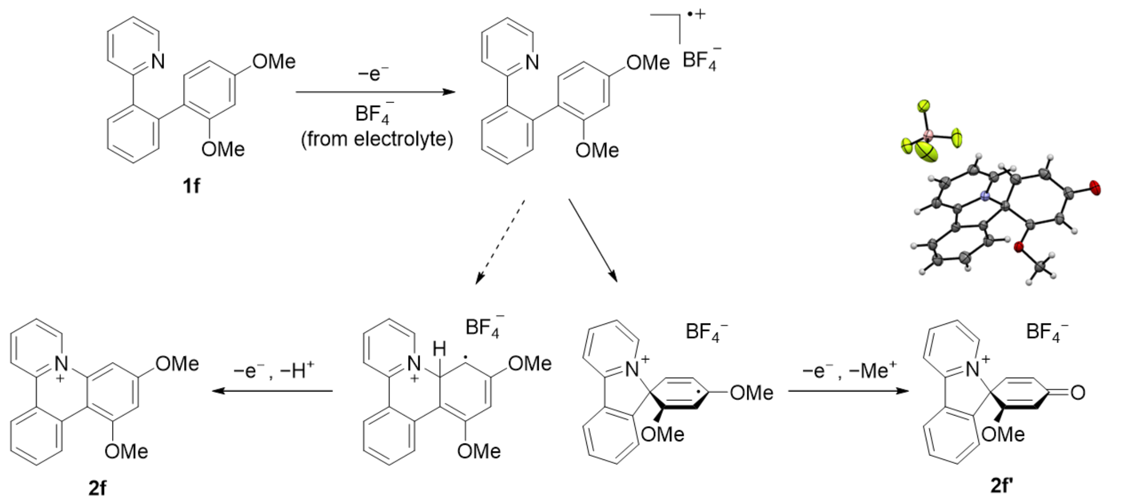

Figure S3. The plausible mechanism for spiro cyclization of **1f** to give **2f'** and its crystal structure.

5. Optical and electrochemical properties

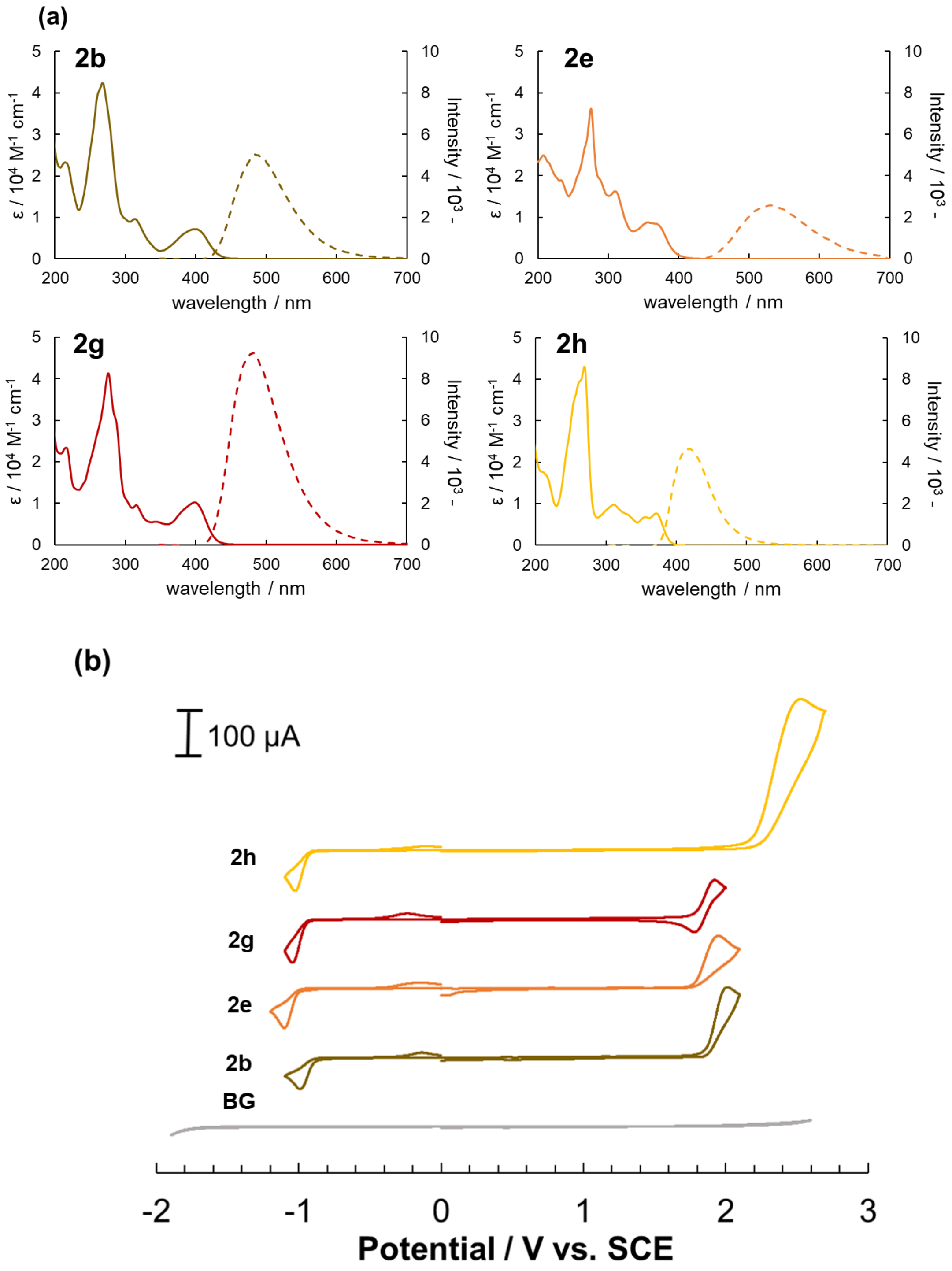

Figure S4. (a) UV-vis absorption and fluorescence spectra for **2b**, **2e**, **2g**, **2h** in MeCN solutions. (b) Cyclic voltammograms of 3 mM **2b**, **2e**, **2g**, **2h** in 0.1 M Bu_4NPF_6/MeCN at a scan rate of 100 mV/s.

Table S1. Summary of the optical and electrochemical properties of the N^+-doped PAH products.

Compound	λ_{max}^{abs} (nm)^a		λ_{max}^{em} (nm)^b	Φ_{FL}^c	$E_{1/2}^{red}$ (V vs. SCE)^d	$E_{1/2}^{ox}$ (V vs. SCE)^d	LUMO (eV)^e	HOMO (eV)^e	$\Delta E_{HOMO-LUMO}$ (eV)
	π–π*	ICT							
**2a**	270	372	436	0.57	−1.00	2.11	−3.39	−6.50	3.11
**2b**	268	399	484	0.53	−0.93	1.92	−3.46	−6.31	2.85
**2d**	277	401	484	0.62	−1.03	1.76	−3.36	−6.15	2.79
**2e**	276	357	530	0.26	−1.05	1.85	−3.34	−6.24	2.90
**2g**	276	398	482	0.62	−0.99	1.85	−3.40	−6.24	2.84
**2h**	269	371	420	0.39	−0.97	2.33	−3.42	−6.72	3.30
**2i**	266	370	420	0.27	−0.93	2.42	−3.46	−6.81	3.35
**2j**	253	391	468	0.11	−0.90	2.00	−3.49	−6.39	2,90

$^a\lambda_{max}^{abs}$ indicates the absorption maximum wavelength for π–π* and intramolecular charge transfer (ICT) absorption measured in solution. $^b\lambda_{max}^{em}$ indicates the wavelength of an emission peak in a fluorescence spectrum. $^c\Phi_{FL}$ indicates the internal quantum efficiency in solution using the integrating sphere method. ^{d}Reduction and oxidation half-wave potentials determined by CV measurement. ^{e}LUMO and HOMO were estimated by the equations: LUMO = $-(E_{1/2}^{red} + 4.39)$, HOMO = $-(E_{1/2}^{ox} + 4.39)$, respectively.

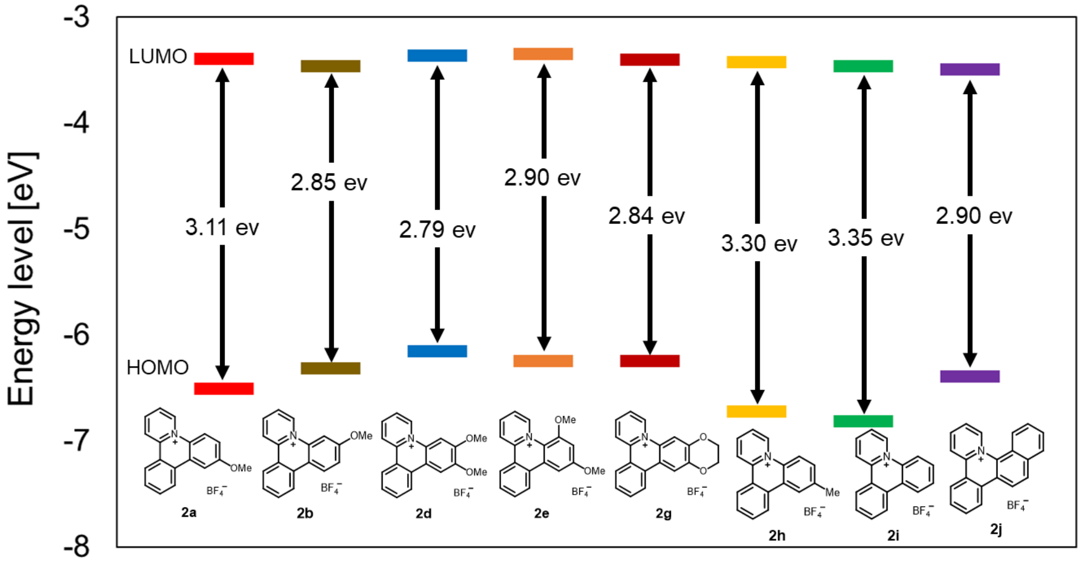

Figure S5. HOMO-LUMO gaps of **2a–2j**.

6. TD DFT simulations for 2a–2j

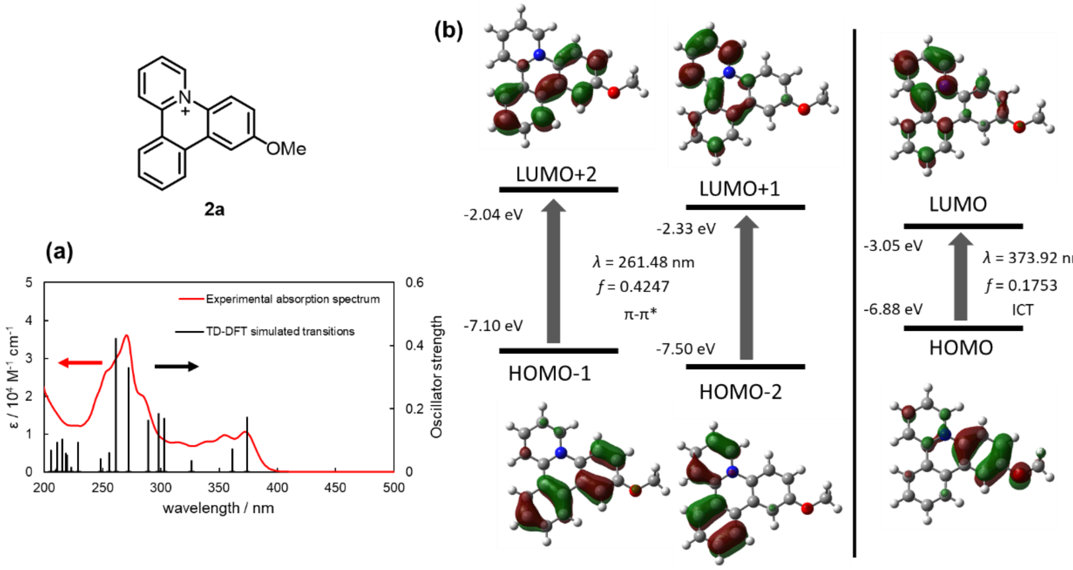

Figure S6. (a) Absorption spectrum of **2a** and oscillator strength calculated at B3LYP/6-311G++(2df, 2p) level basis set. (b) MOs of HOMO, HOMO−1, HOMO−2, LUMO, LUMO+1, LUMO+2 are described (iso value = 0.04).

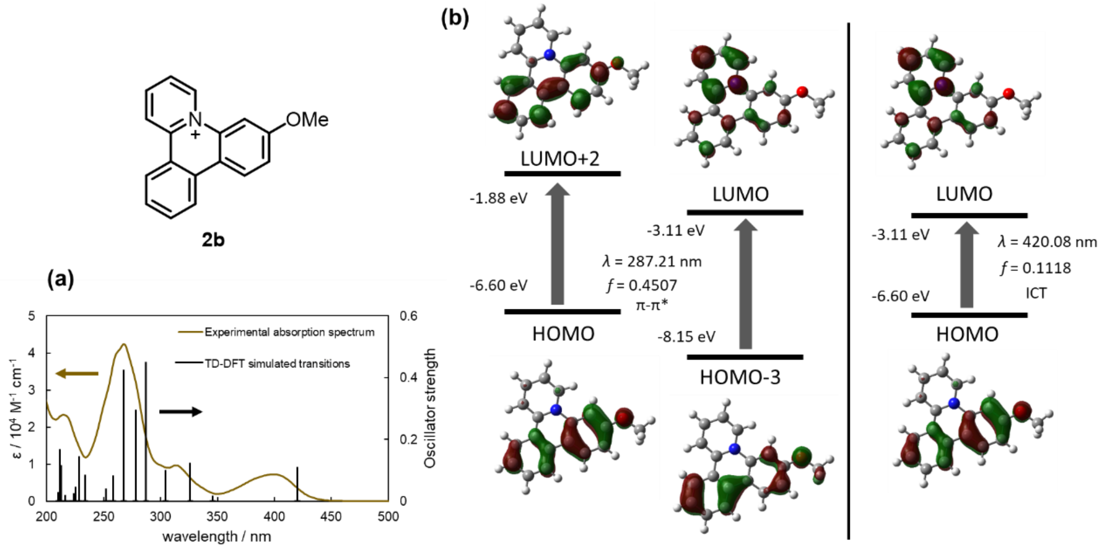

Figure S7. (a) Absorption spectrum of **2b** and oscillator strength calculated at B3LYP/6-311G++(2df, 2p) level basis set. (b) MOs of HOMO, HOMO−3, LUMO, LUMO+2 are described (iso value = 0.04).

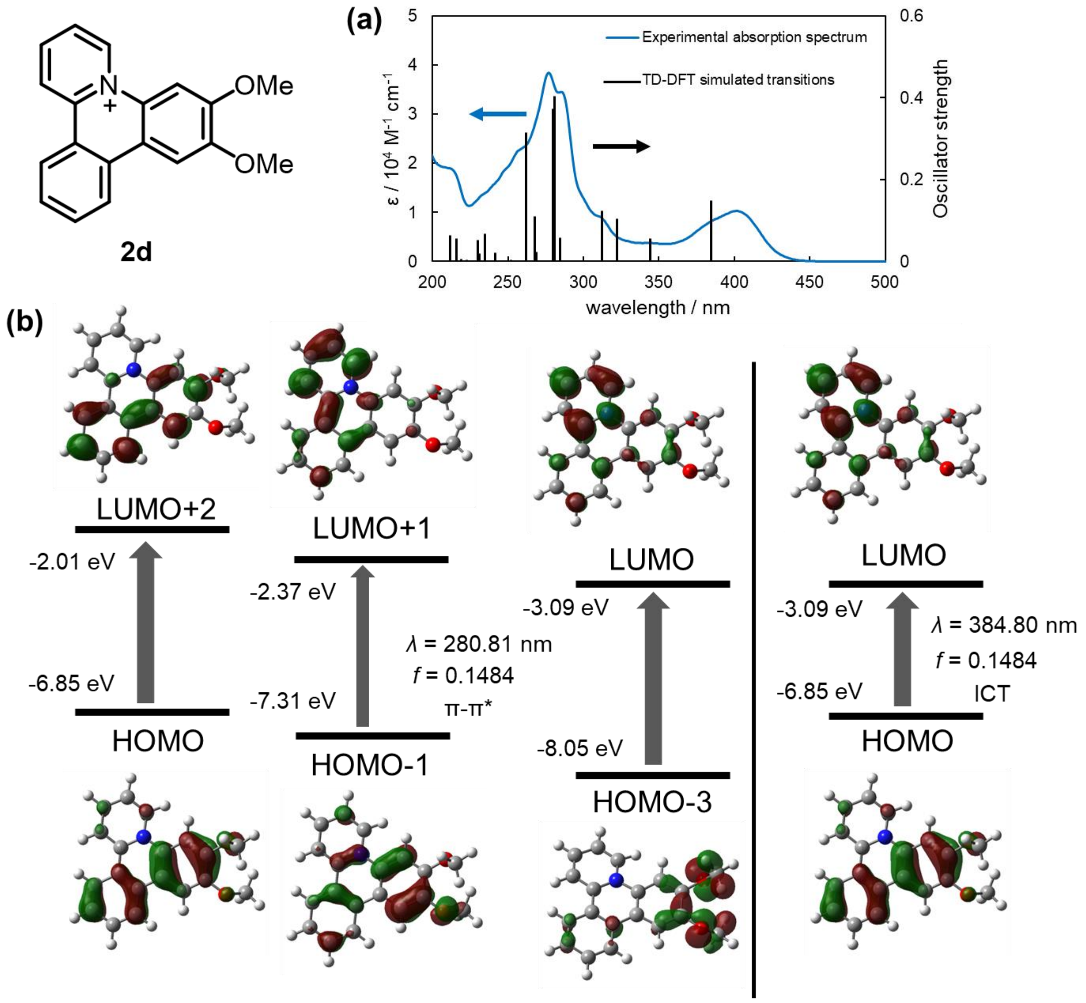

Figure S8. (a) Absorption spectrum of **2d** and oscillator strength calculated at B3LYP/6-311G++(2df, 2p) level basis set. (b) MOs of HOMO, HOMO−1, HOMO−3, LUMO, LUMO+1, LUMO+2 are described (iso value = 0.04).

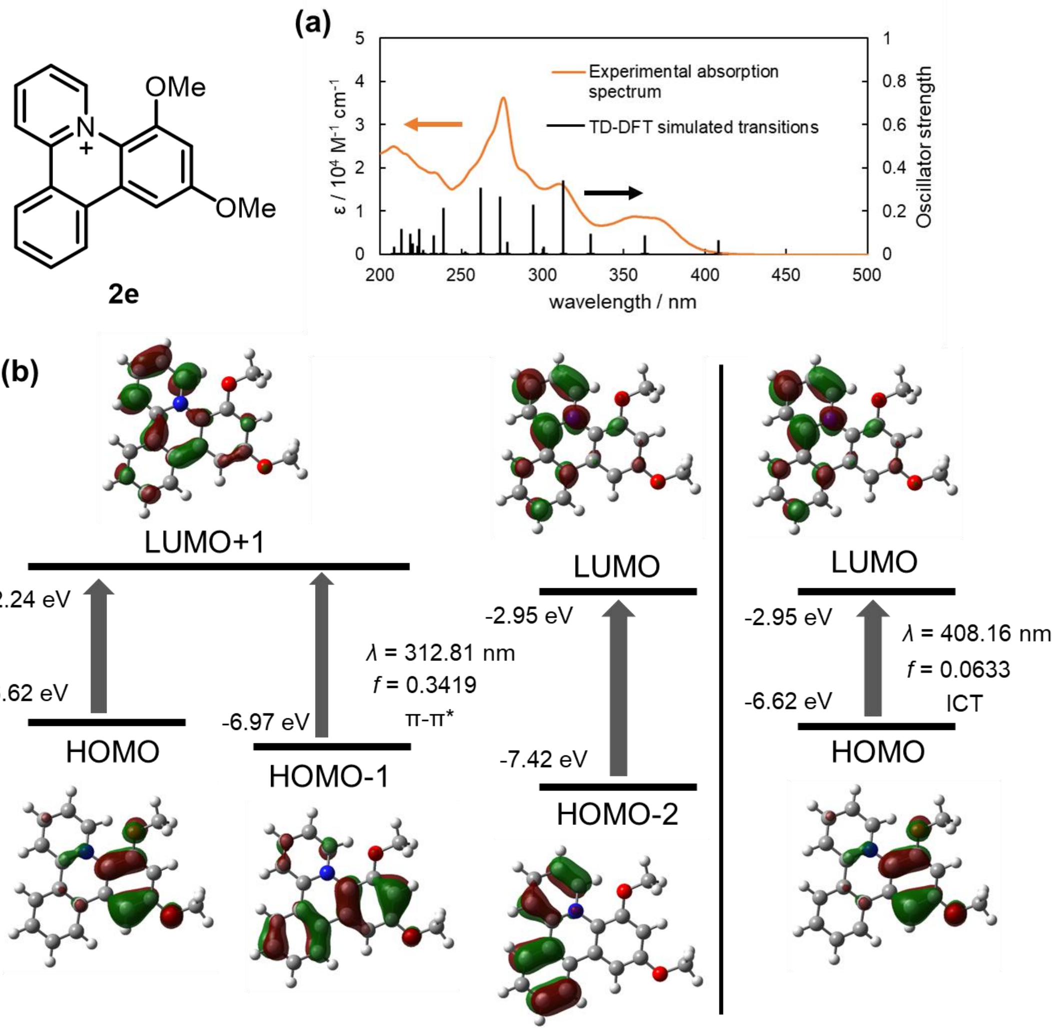

Figure S9. (a) Absorption spectrum of **2e** and oscillator strength calculated at B3LYP/6-311G++(2df, 2p) level basis set. (b) MOs of HOMO, HOMO−1, HOMO−2, LUMO, LUMO+1 are described (iso value = 0.04).

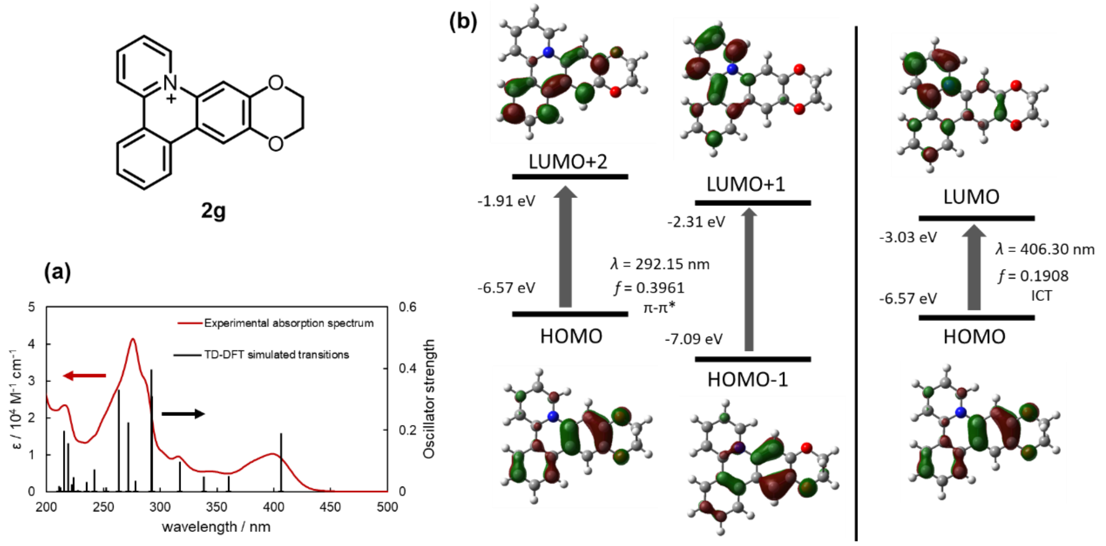

Figure S10. (a) Absorption spectrum of **2g** and oscillator strength calculated at B3LYP/6-311G++(2df, 2p) level basis set. (b) MOs of HOMO, HOMO−1, LUMO, LUMO+1, LUMO+2 are described (iso value = 0.04).

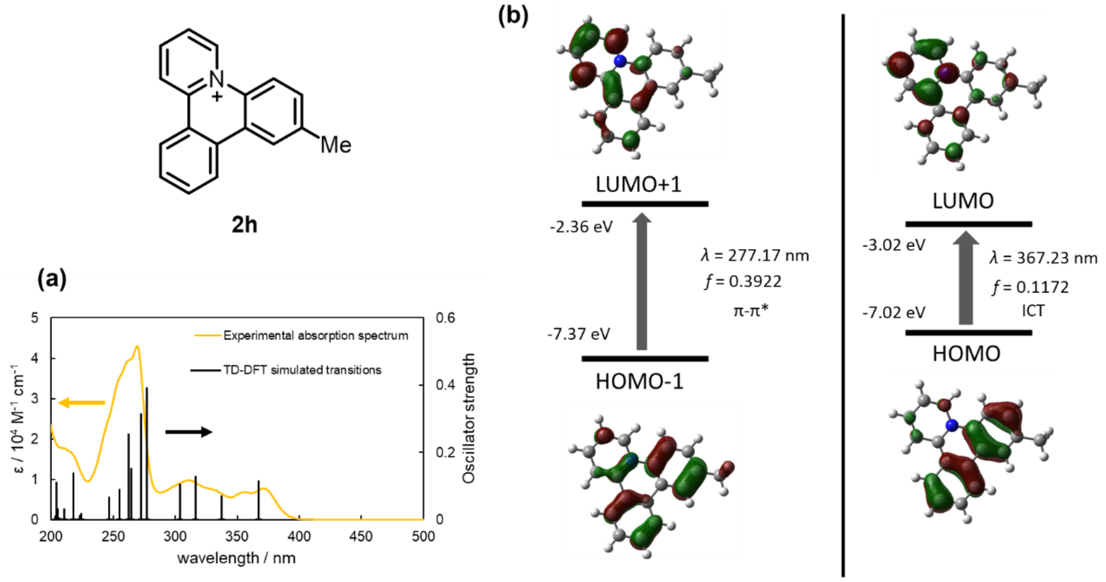

Figure S11. (a) Absorption spectrum of **2h** and oscillator strength calculated at B3LYP/6-311G++(2df, 2p) level basis set. (b) MOs of HOMO, HOMO−1, LUMO, LUMO+1 are described (iso value = 0.04).

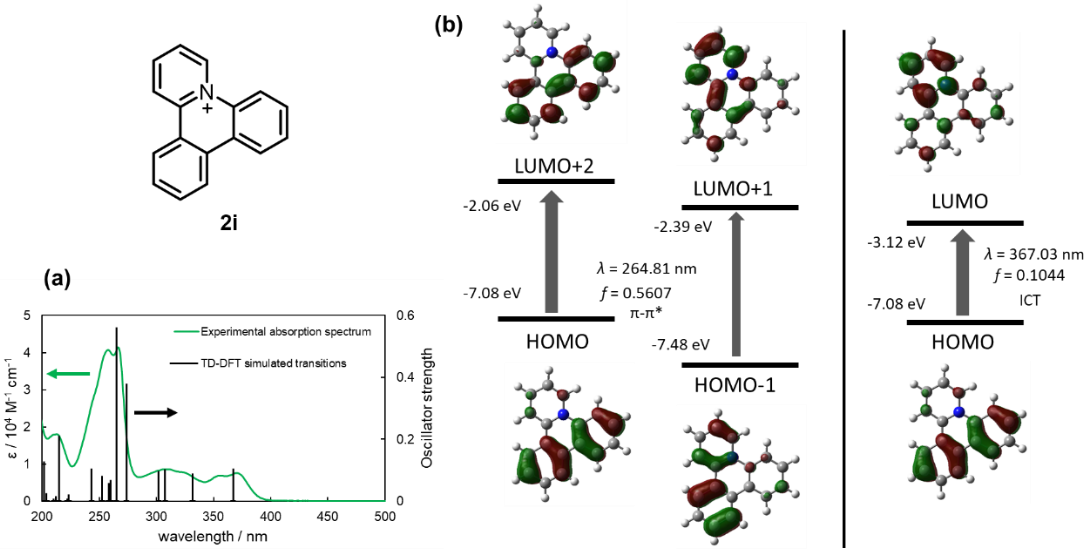

Figure S12. (a) Absorption spectrum of **2i** and oscillator strength calculated at B3LYP/6-311G++(2df, 2p) level basis set. (b) MOs of HOMO, HOMO−1, LUMO, LUMO+1, LUMO+2 are described (iso value = 0.04).

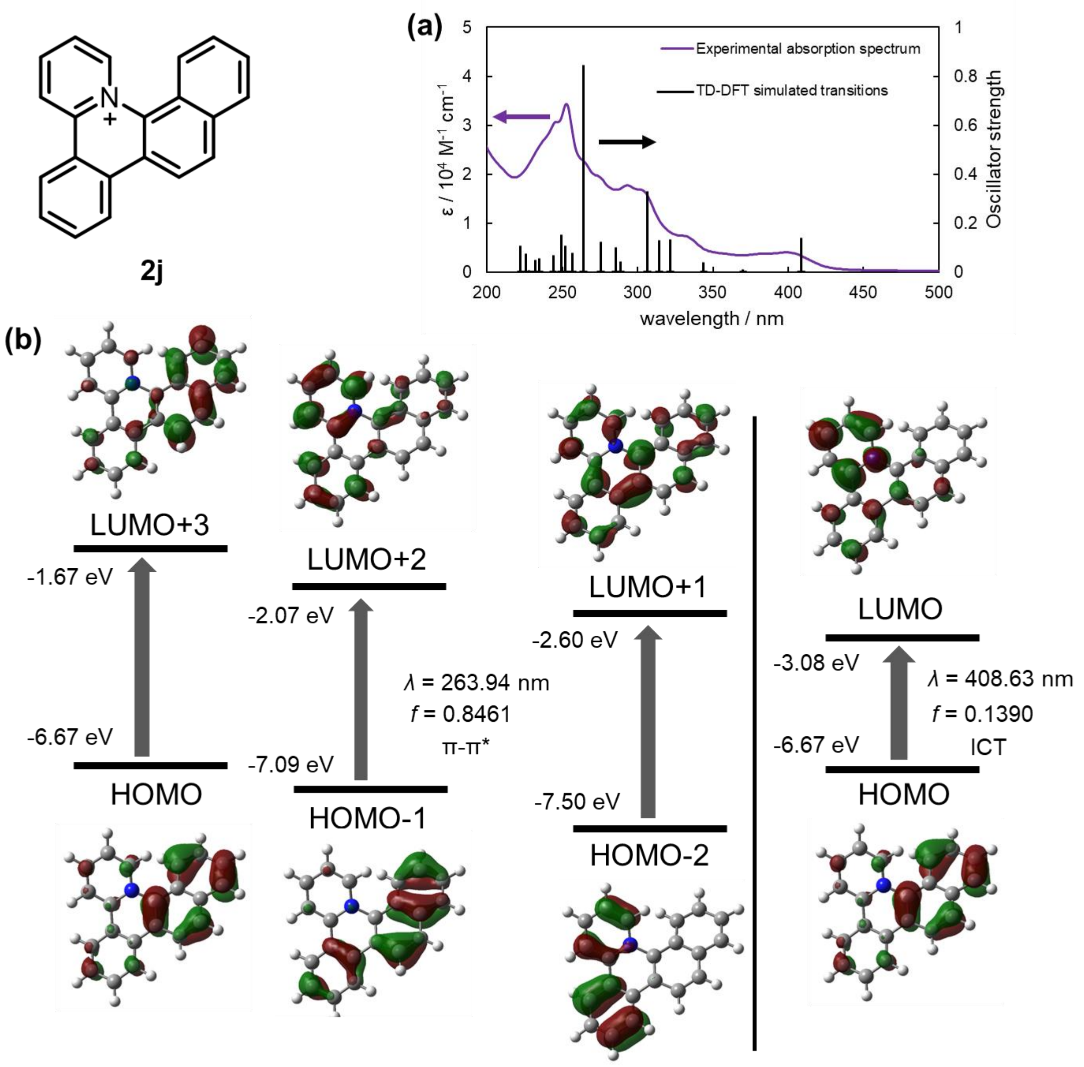

Figure S13. (a) Absorption spectrum of **2j** and oscillator strength calculated at B3LYP/6-311G++(2df, 2p) level basis set. (b) MOs of HOMO, HOMO−1, HOMO-2, LUMO, LUMO+1, LUMO+2, LUMO+3 are described (iso value = 0.04).

Table S2. Summary of simulated transitions of **2a–2j**.

Compound	ransition typ	λ [nm][a)]	$f^{b)}$	Contribution
**2a**	ICT	373.92	0.1753	HOMO→LUMO (0.67965)
	π-π*	261.48	0.4247	HOMO-2→LUMO+1 (0.49648)<br>HOMO-1→LUMO+2 (-0.43365)
**2b**	ICT	420.08	0.1118	HOMO→LUMO (0.69943)
	π-π*	287.21	0.4507	HOMO→LUMO+2 (0.56581)<br>HOMO-3→LUMO (0.33900)
**2d**	ICT	384.80	0.1484	HOMO→LUMO (0.68948)
	π-π*	280.81	0.4031	HOMO-3→LUMO (0.33315)<br>HOMO-1→LUMO+1 (0.47567)<br>HOMO→LUMO+2 (-0.33648)
**2e**	ICT	408.16	0.0633	HOMO→LUMO (0.69590)
	π-π*	312.81	0.3419	HOMO-2→LUMO (0.41263)<br>HOMO-1→LUMO+1 (-0.31171)<br>HOMO→LUMO+1 (-0.41564)
**2g**	ICT	406.30	0.1908	HOMO→LUMO (0.68831)
	π-π*	292.15	0.3961	HOMO-1→LUMO+1 (-0.46790)<br>HOMO→LUMO+2 (0.48489)
**2h**	ICT	367.23	0.1172	HOMO→LUMO (0.68724)
	π-π*	277.17	0.3922	HOMO-1→LUMO+1 (0.60438)
**2i**	ICT	367.03	0.1044	HOMO→LUMO (0.69054)
	π-π*	264.81	0.5607	HOMO-1→LUMO+1 (0.42535)<br>HOMO→LUMO+2 (-0.42395)
**2j**	ICT	408.63	0.1390	HOMO→LUMO (0.69492)
	π-π*	263.94	0.8461	HOMO-2→LUMO+1 (0.32157)<br>HOMO-1→LUMO+2 (0.41087)<br>HOMO→LUMO+3 (-0.34153)

[a)] Wavelength. [b)] Oscillator strength.

7. Comparison of physical properties between 2i and triphenylene

Because compound **2i** is an N^{+}-doped analogue of triphenylene, we comprehensively compared its physical properties with those of triphenylene. Given the low solubility of triphenylene in MeCN, dichloromethane (CH_2Cl_2) was used for each measurement. In the UV–vis absorption spectrum of triphenylene, sharp signals were observed in the wavelength region below 300 nm, which is assignable to the π–π* transition. No ICT absorption was observed in the longer-wavelength area, where donor–acceptor-type molecule **2i** exhibited ICT absorption. In the FL analysis, **2i** exhibited an emission peak at a longer wavelength compared with the wavelength of the emission peak of triphenylene. Compared with the oxidation and reduction potentials of triphenylene, those of **2i** were positively shifted because of its cationic nature. However, because of the great

contribution of the N^+-doping toward lowering the LUMO levels, the estimated HOMO–LUMO energy gap of **2i** was substantially smaller than that of triphenylene. The N^+-doping of PAHs drastically changed their physical properties; therefore, the present method is an easy and powerful approach to creating N^+-doped PAHs.

Table S3. Summary of optical and electrochemical properties of **2i** and triphenylene.

Compound	λ_{max}^{abs} (nm)		λ_{onset}^{abs} (nm)	λ_{max}^{em} (nm)	Φ_{FL}	$\Delta E_{HOMO\text{-}LUMO}$[a)] (eV)	E_{onset}^{red} (V vs SCE)	E_{onset}^{ox} (V vs SCE)
	$\pi-\pi*$	ICT						
**2i**	270	380	399	416	0.21	3.11	−0.79	–
**triphenylene**	260	–	299	354	0.076	4.15	–	1.77

a) Obtained by λ_{onset}^{abs}

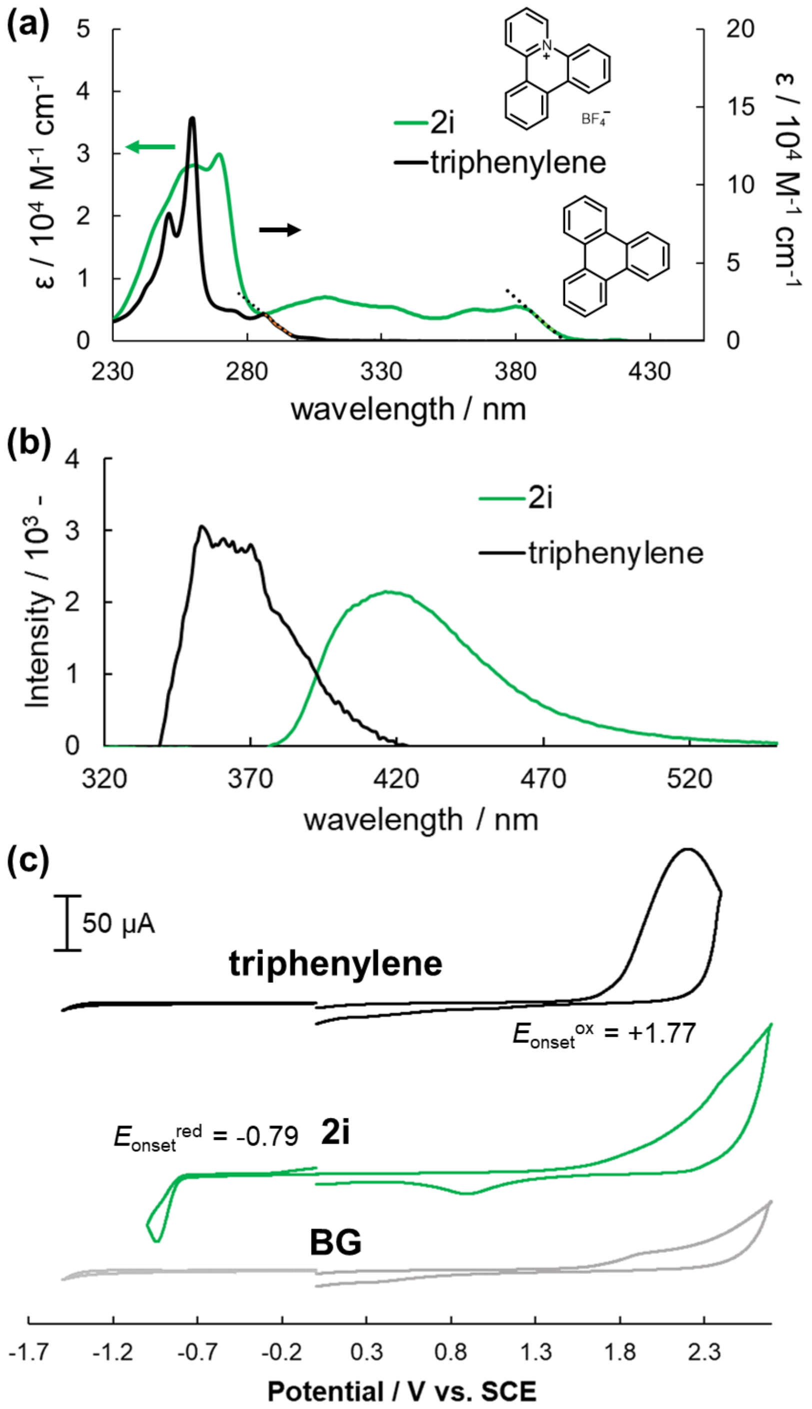

Figure S14. (a) Absorption spectra of **2i** and triphenylene in CH_2Cl_2. Because the solubility of **2i** in CH_2Cl_2 was low, the exact molar absorption coefficient was not determined. (b) Emission spectra of **2i** and triphenylene in CH_2Cl_2. (c) Cyclic voltammograms of 3 mM **2i** and triphenylene in 0.2 M Bu_4NPF_6/CH_2Cl_2 at a scan rate of 100 mV/s.

8. Single crystal X-ray diffraction

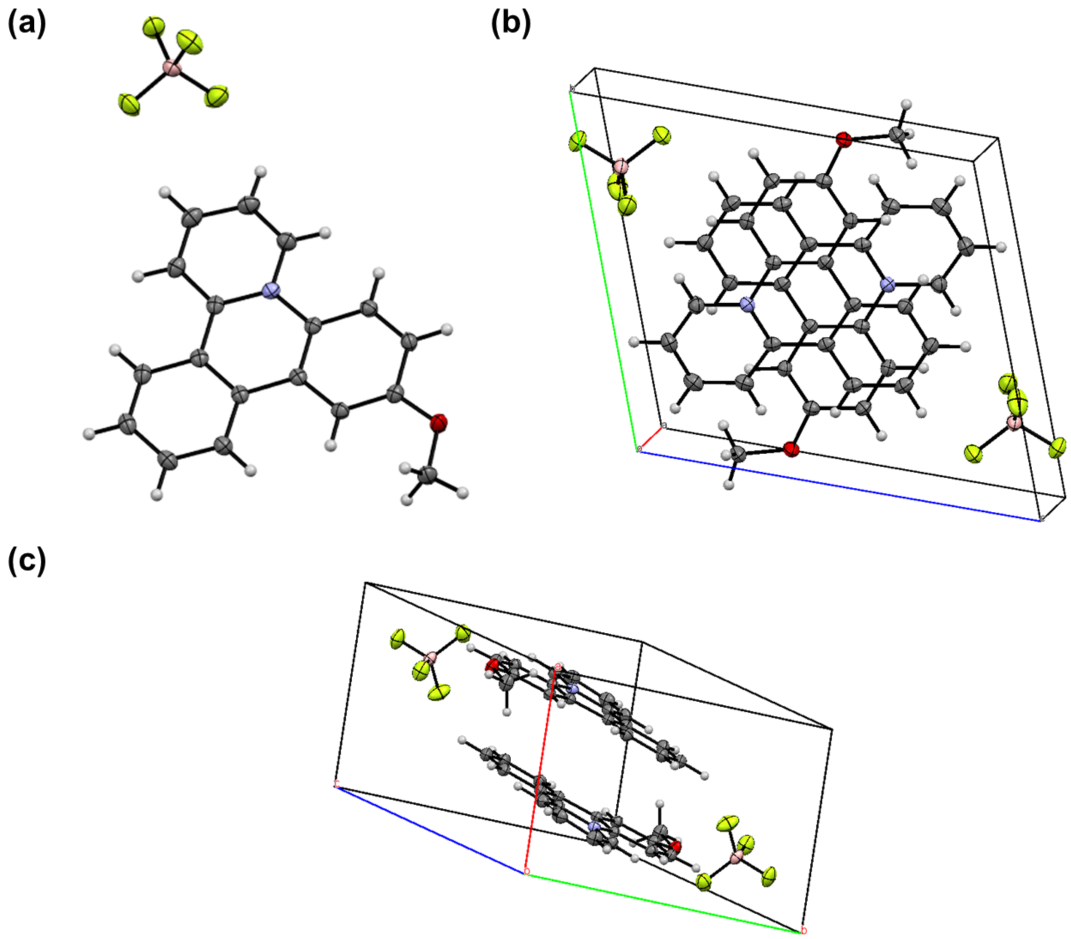

Figure S15. (a) The ORTEP drawing of **2a** with the ellipsoid contour probability level of 50%. (b, c) Packing inside the crystal of **2a**.

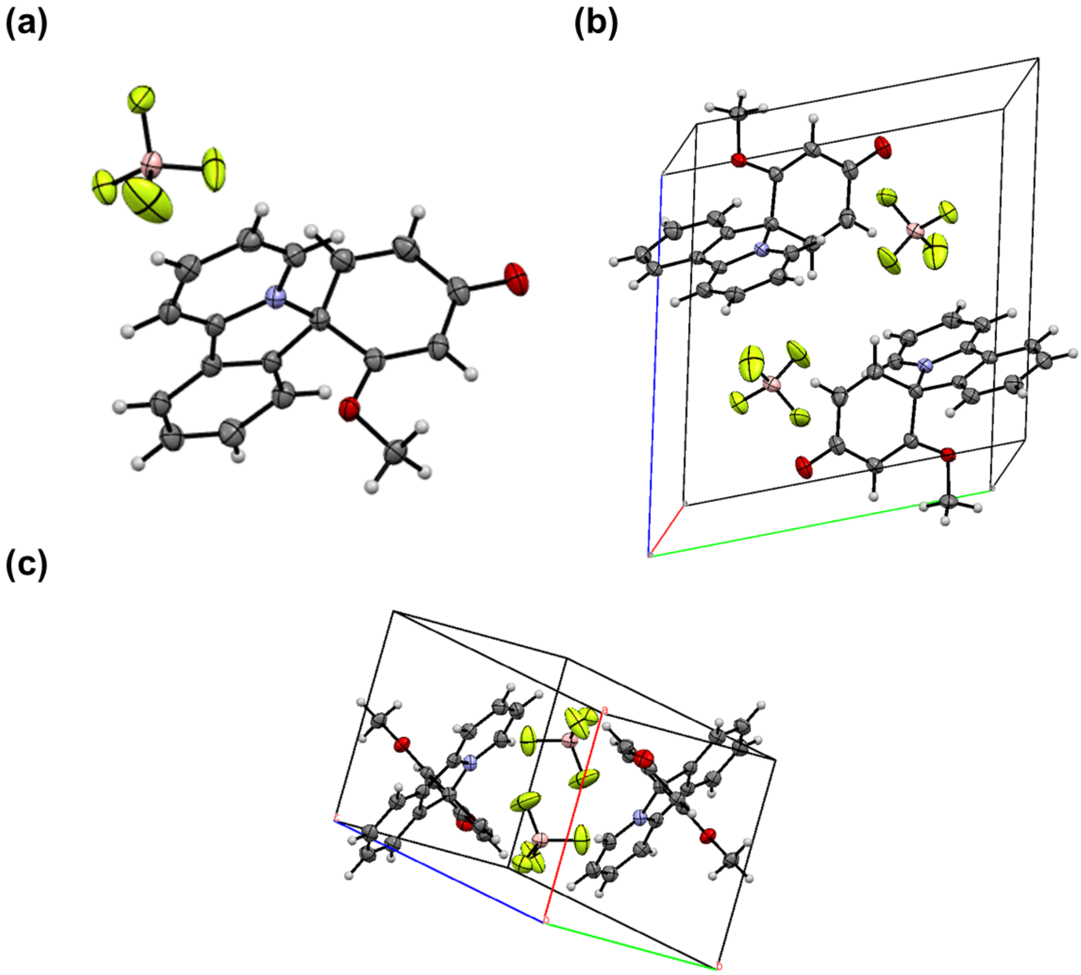

Figure S16. (a) The ORTEP drawing of **2f'** with the ellipsoid contour probability level of 50%. (b, c) Packing inside the crystal of **2f'**.

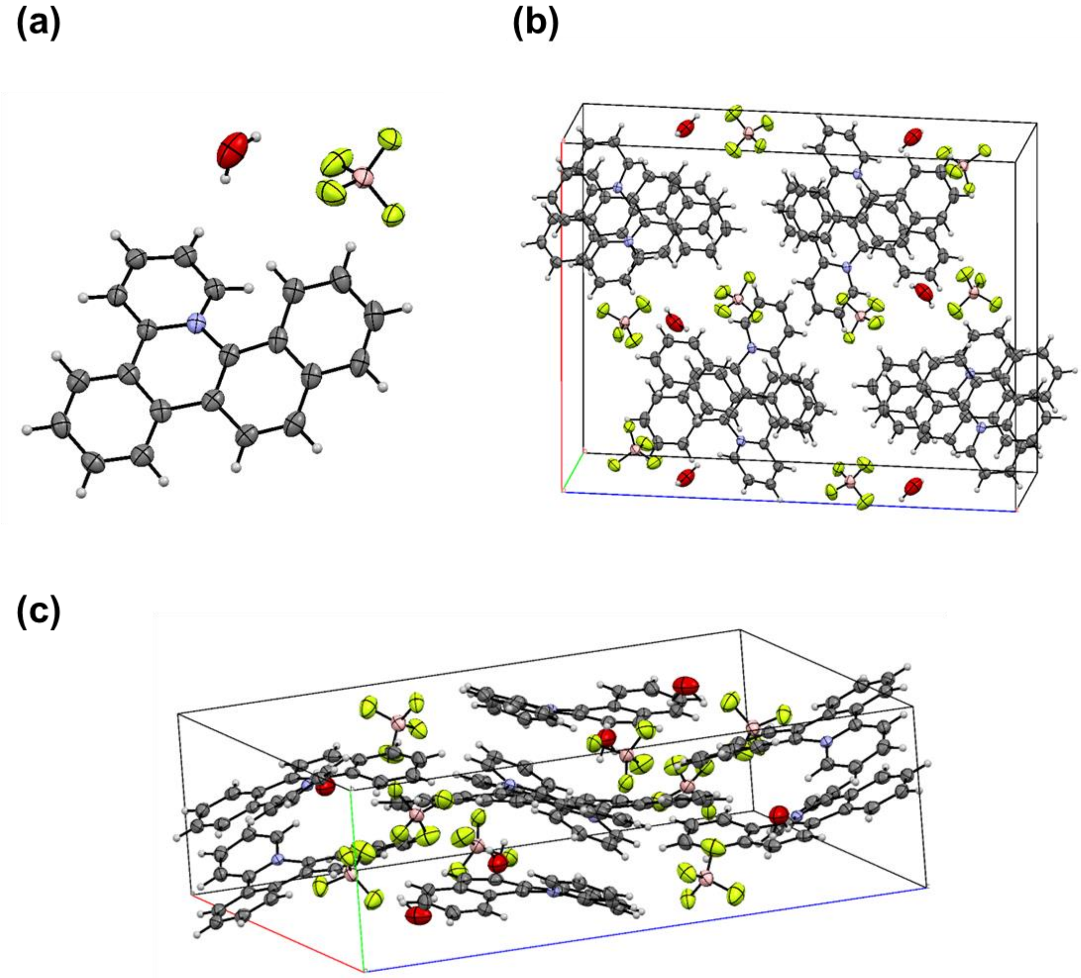

Figure S17. (a) The ORTEP drawing of **2j** with the ellipsoid contour probability level of 50%. (b, c) Packing inside the crystal of **2j**.

Table S4. Crystallographic data of **2a**, **2f'**, and **2j**.

Crystal data	**2a**	**2f'**	**2j**
CCDC	2255063	2255064	2255066
Empirical Formula	C18H14BF4NO	C18H14BF4NO2	C21H14BF4NO0.5
Formula Weight	347.11	363.11	375.14
h, k, lmax	5, 12, 13	6, 12, 13	23, 6, 29
Crystal System	triclinic	triclinic	orthorhombic
Space Group	P-1	P-1	Pbcn
a, Å	7.1179(2)	7.2479(4)	18.8691(6)
b, Å	10.0789(4)	10.2994(7)	7.3062(2)
c, Å	11.0840(3)	11.3118(7)	23.8143(9)
α, deg	108.685(3)	77.054(6)	90
β, deg	90.903(2)	76.424(5)	90
γ, deg	97.855(3)	82.373(5)	90
Volume, Å	744.68(4)	797.09(9)	3283.07(19)
Dcalcd, g cm^{-3}	1.548	1.513	1.518
Z	2	2	8
F(000)	356.0	372.0	1536.0
Data Collection	Data Collection	Data Collection	Data Collection
Temperature, K	93.15	93.15	93
2θ max, deg	71.11	74.39	76.86
Tmin/Tmax	0.563/1.000	0.629/1.000	0.737/1.000
Refinement	Refinement	Refinement	Refinement
No. of Observed Data	2765	3083	3410
No. of Parameters	227	236	253
R, wR2	0.0337, 0.0973	0.0531, 0.1385	0.0647, 0.1774
S	1.069	1.067	1.056

8. NMR charts

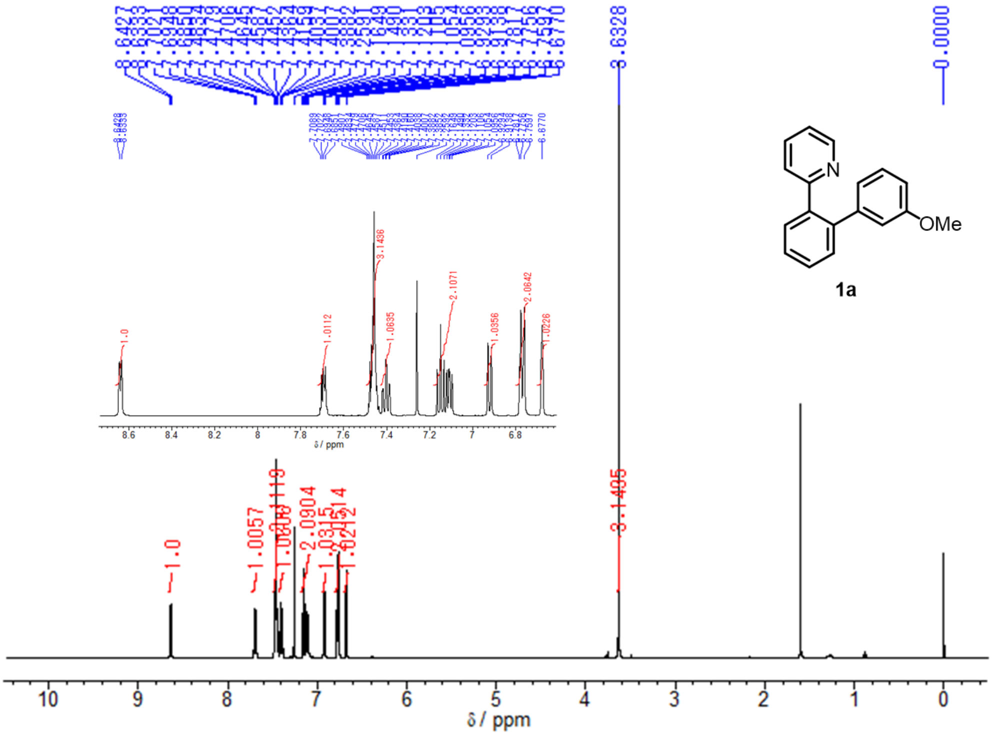

Figure S18. ^{1}H NMR spectrum (500.13 MHz, $CDCl_3$) of **1a**.

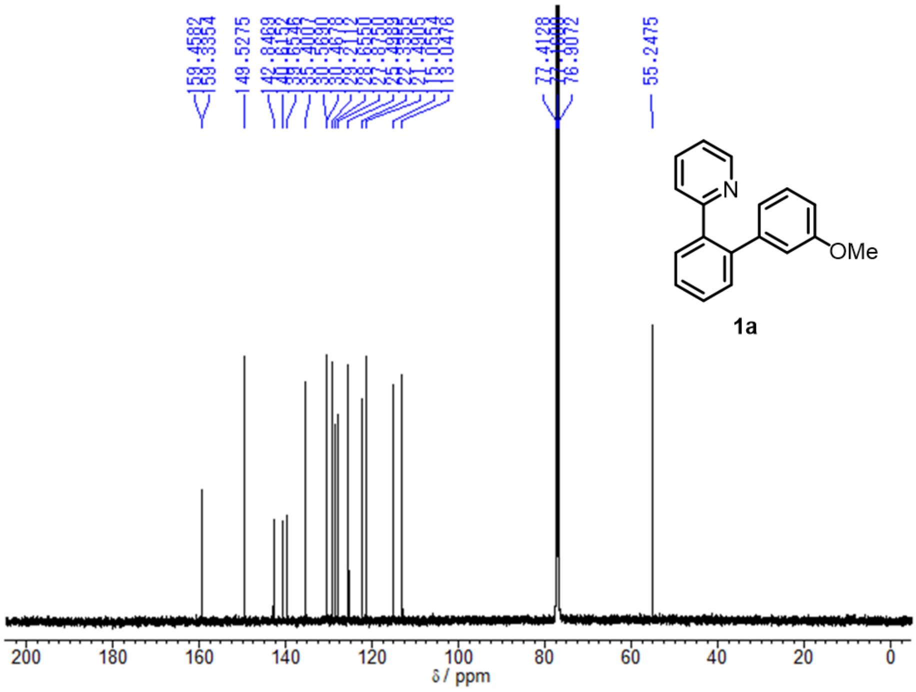

Figure S19. ^{13}C NMR spectrum (125.76 MHz, $CDCl_3$) of **1a**.

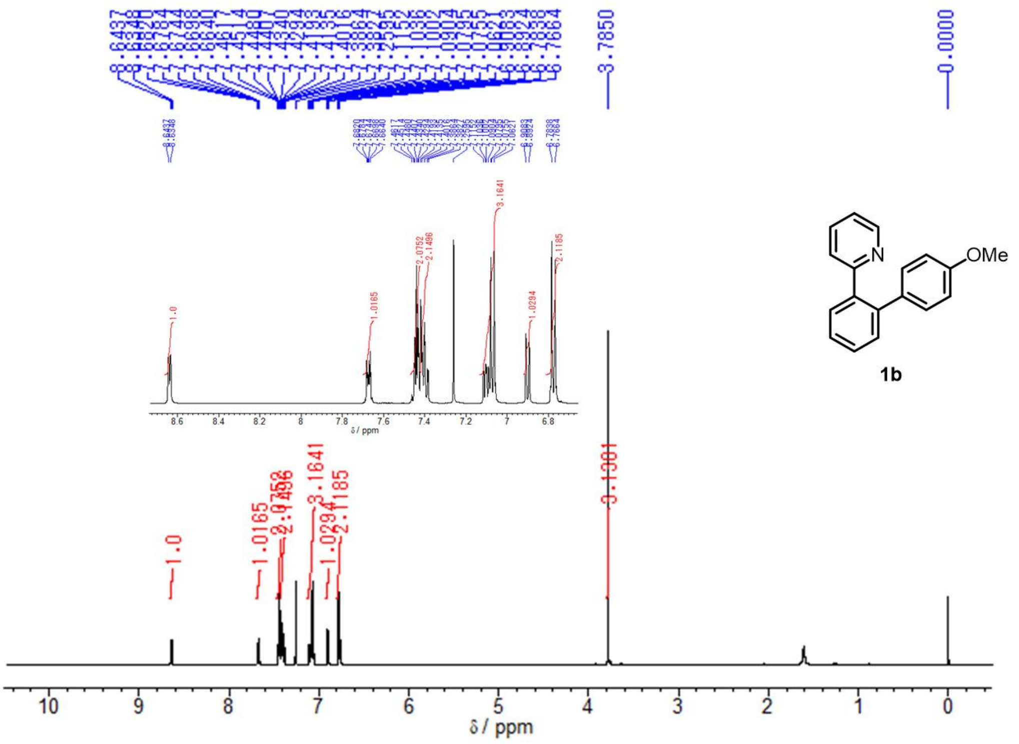

Figure S20. ^{1}H NMR spectrum (500.13 MHz, $CDCl_3$) of **1b**.

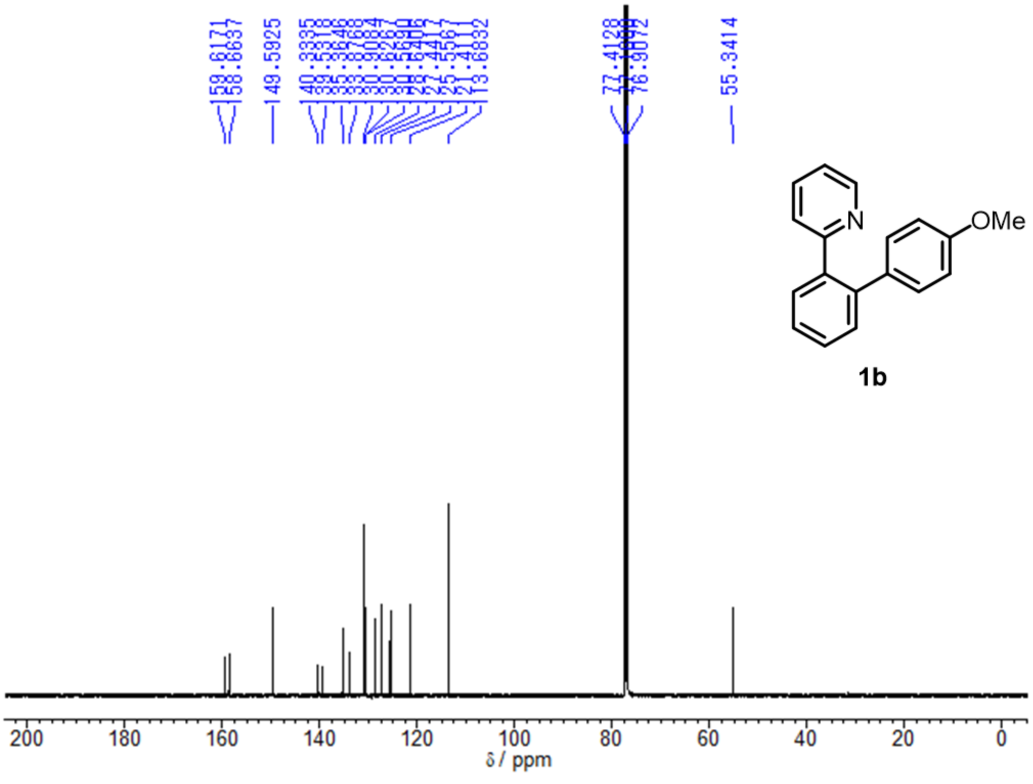

Figure S21. ^{13}C NMR spectrum (125.76 MHz, $CDCl_3$) of **1b**.

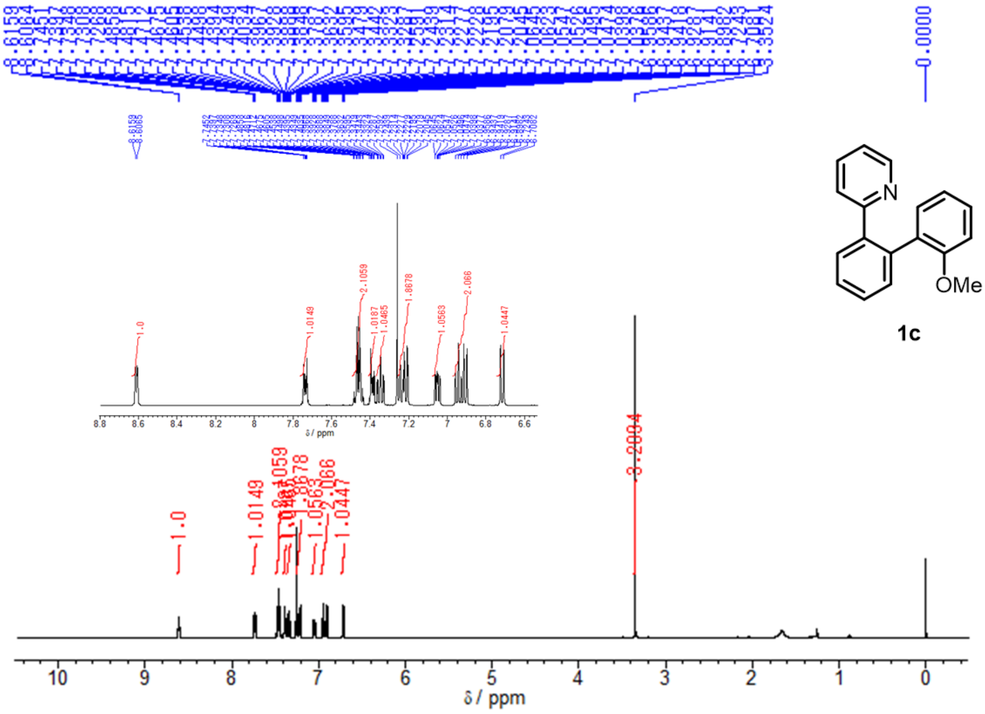

Figure S22. ^{1}H NMR spectrum (500.13 MHz, $CDCl_3$) of **1c**.

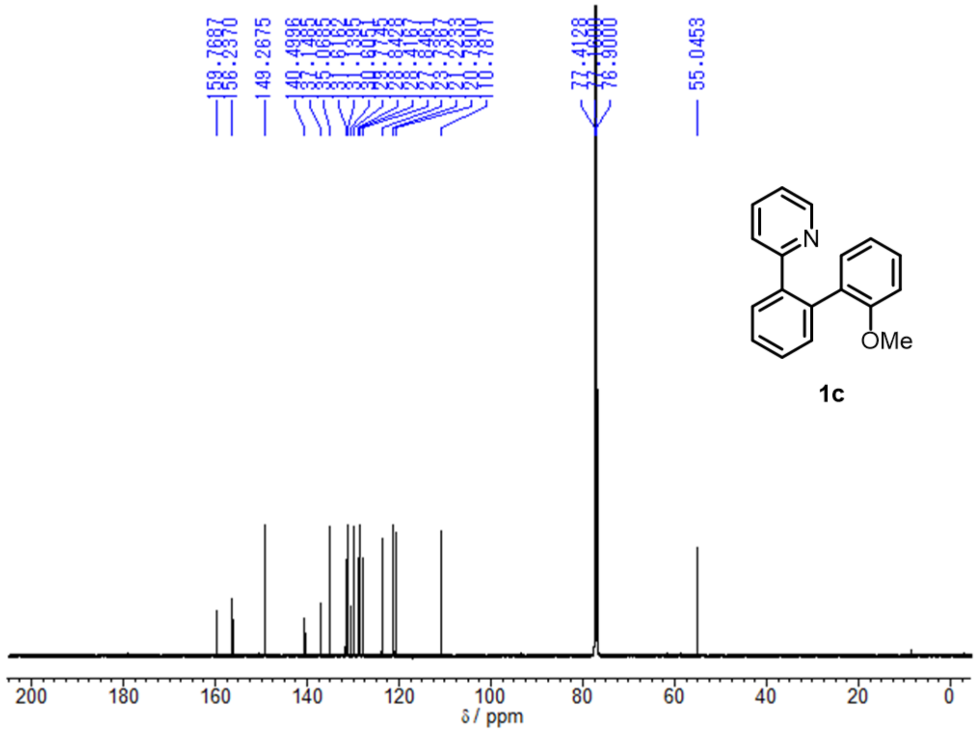

Figure S23. ^{13}C NMR spectrum (125.76 MHz, $CDCl_3$) of **1c**.

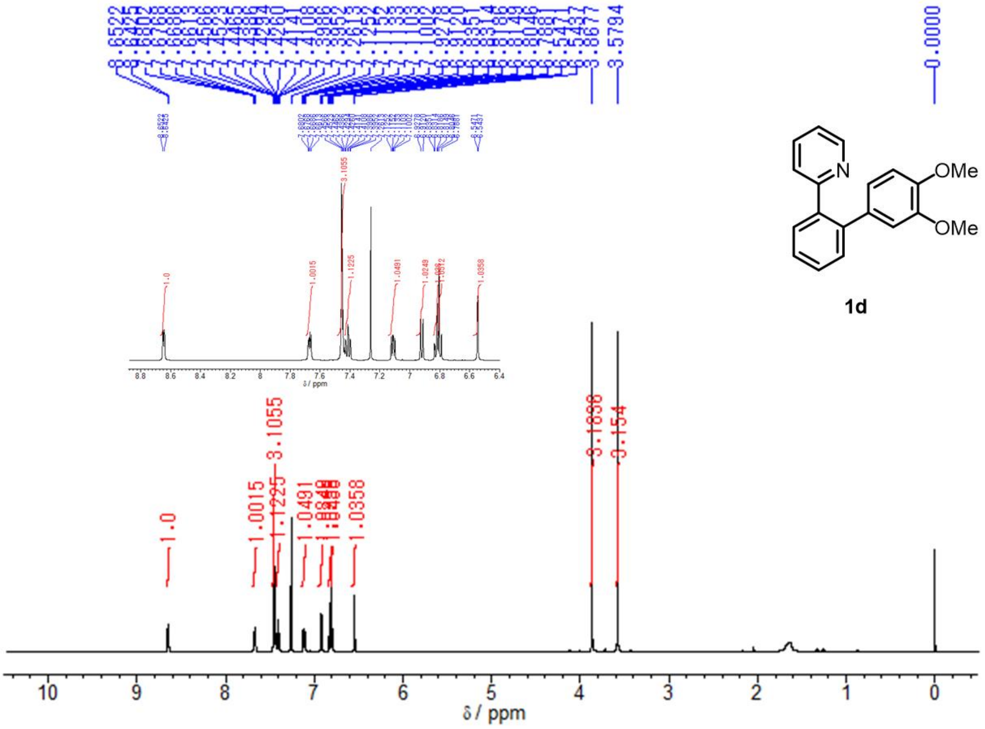

Figure S24. ^{1}H NMR spectrum (500.13 MHz, $CDCl_3$) of **1d**.

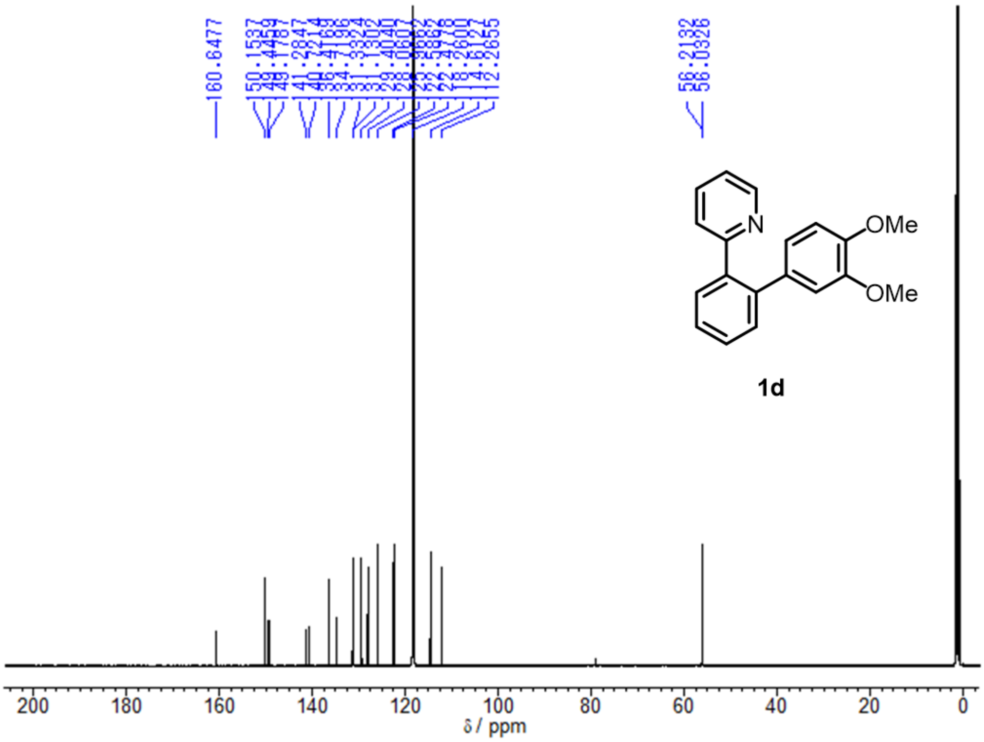

Figure S25. ^{13}C NMR spectrum (125.76 MHz, MeCN-d_3) of **1d**.

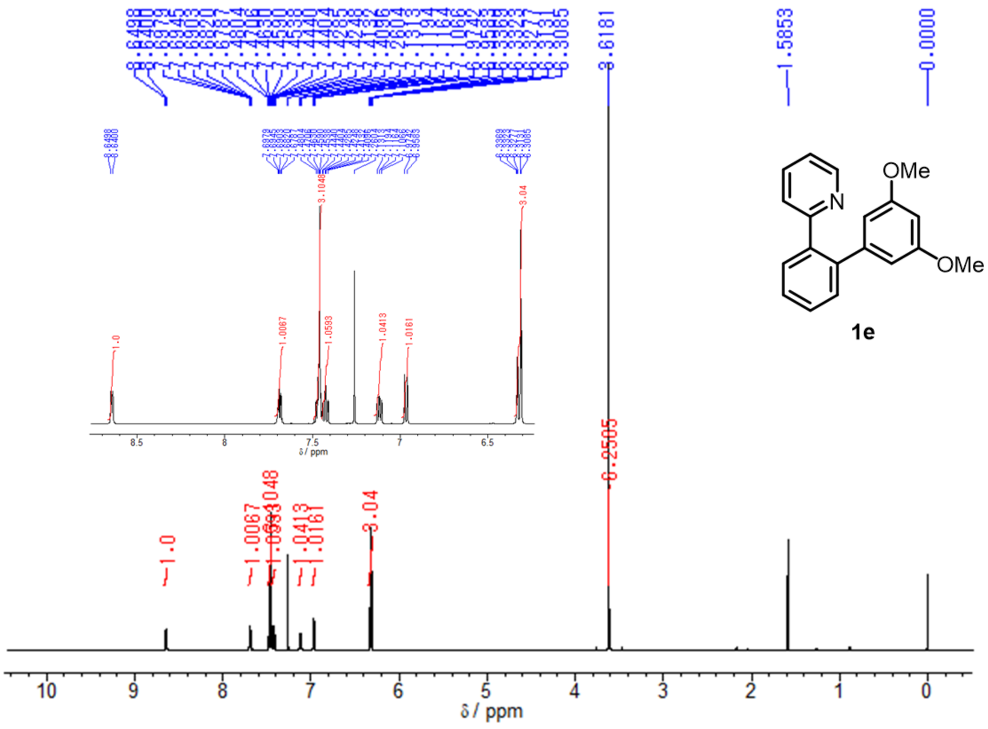

Figure S26. ^{1}H NMR spectrum (500.13 MHz, $CDCl_3$) of **1e**.

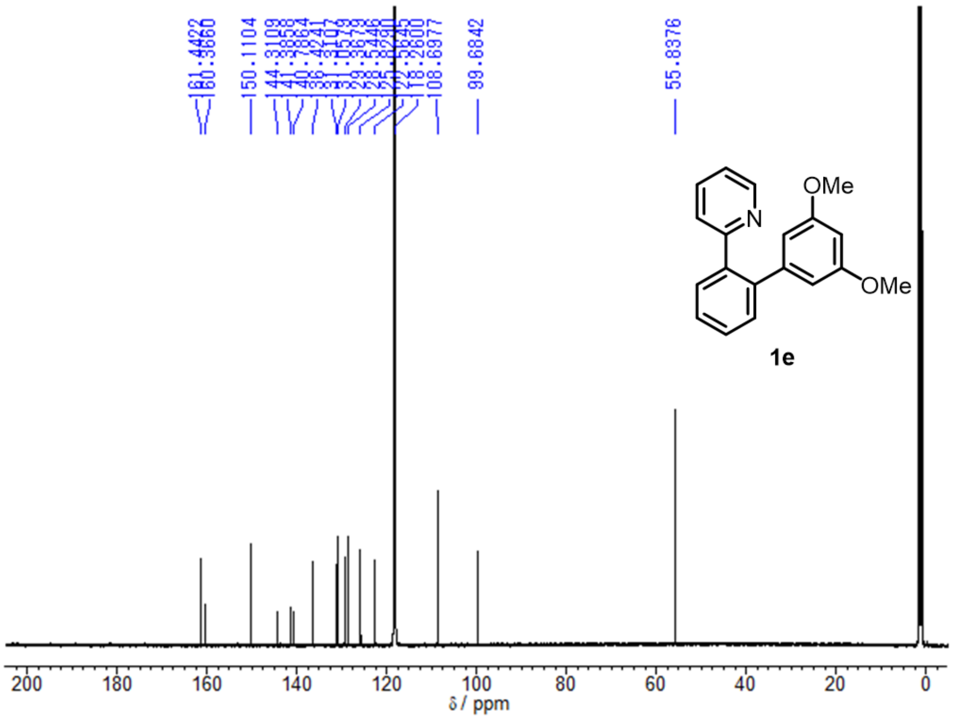

Figure S27. ^{13}C NMR spectrum (125.76 MHz, MeCN-d_3) of **1e**.

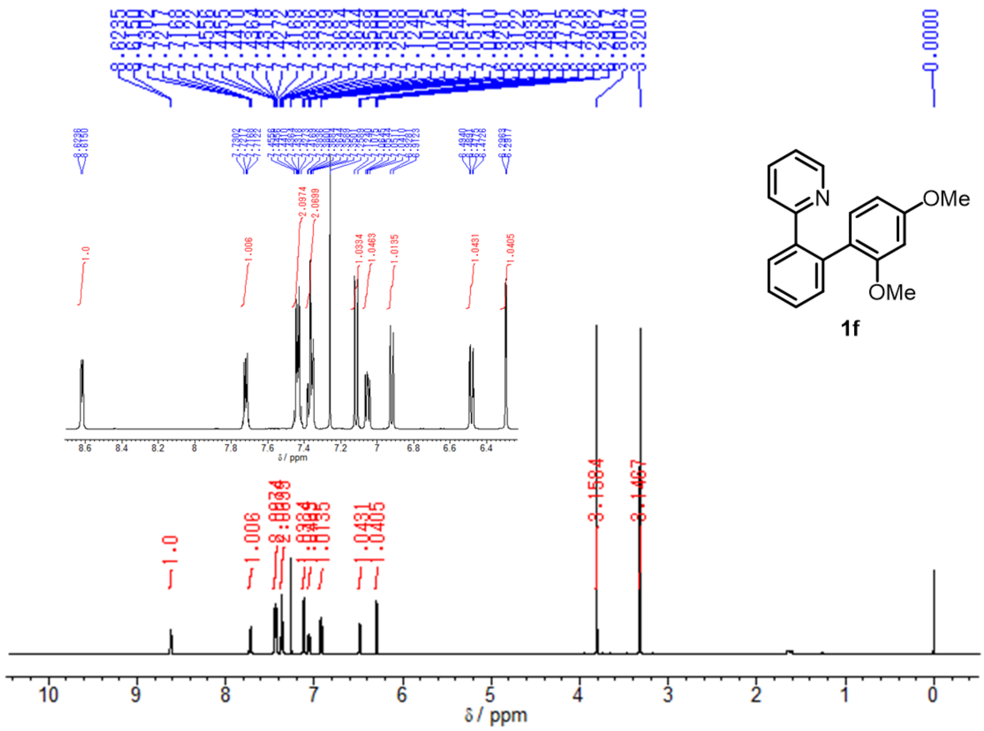

Figure S28. ^{1}H NMR spectrum (500.13 MHz, $CDCl_3$) of **1f**.

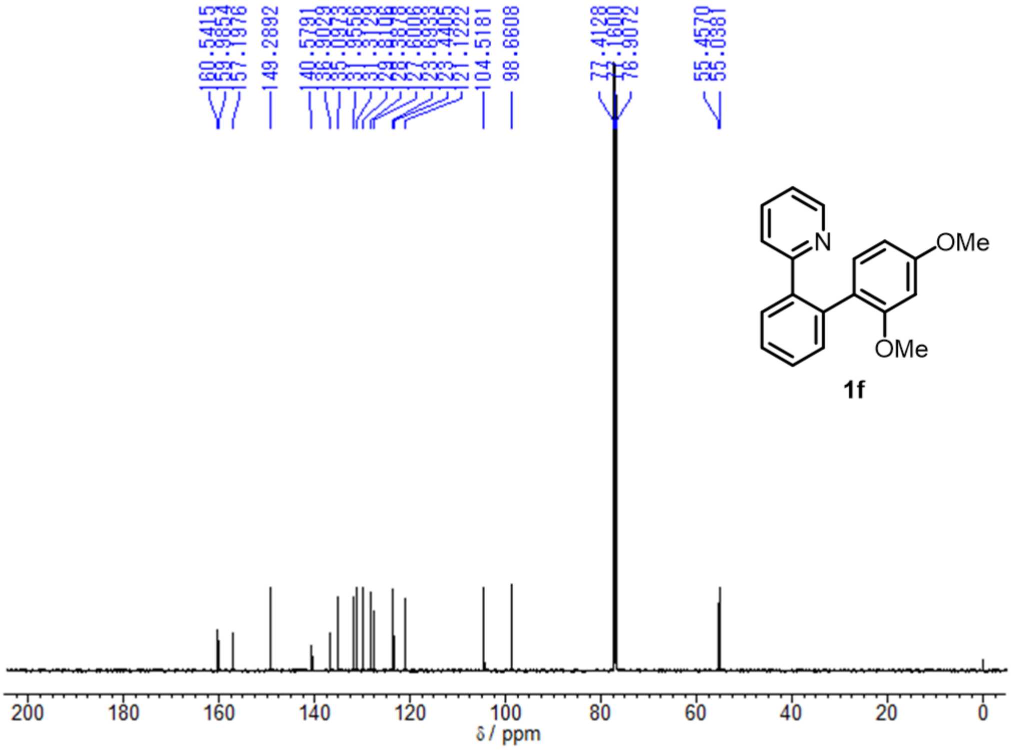

Figure S29. ^{13}C NMR spectrum (125.76 MHz, $CDCl_3$) of **1f**.

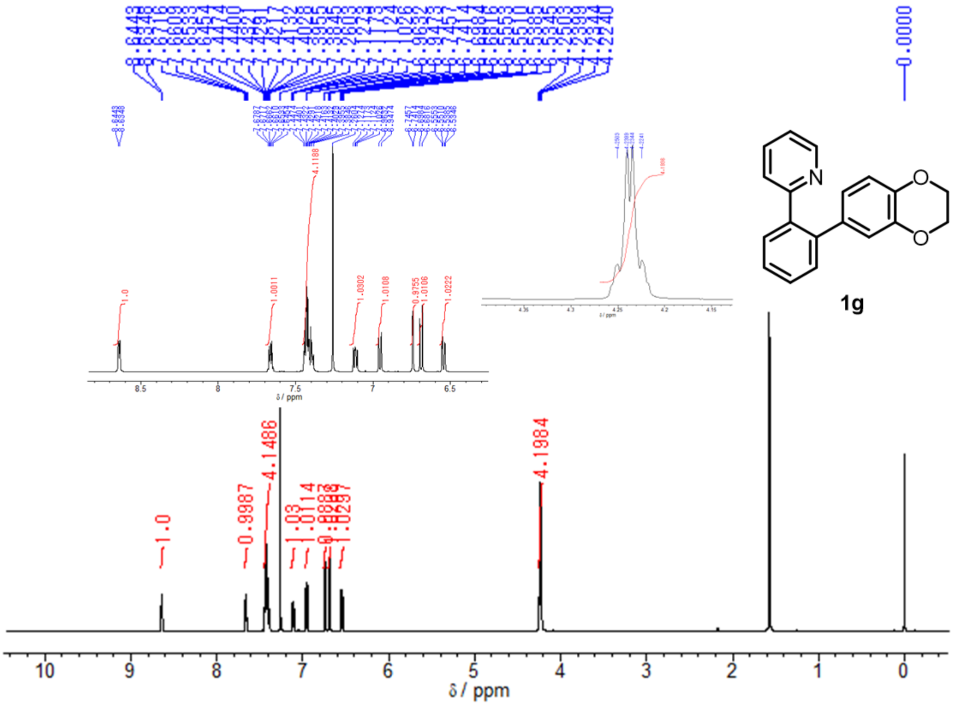

Figure S30. ^{1}H NMR spectrum (500.13 MHz, $CDCl_3$) of **1g**.

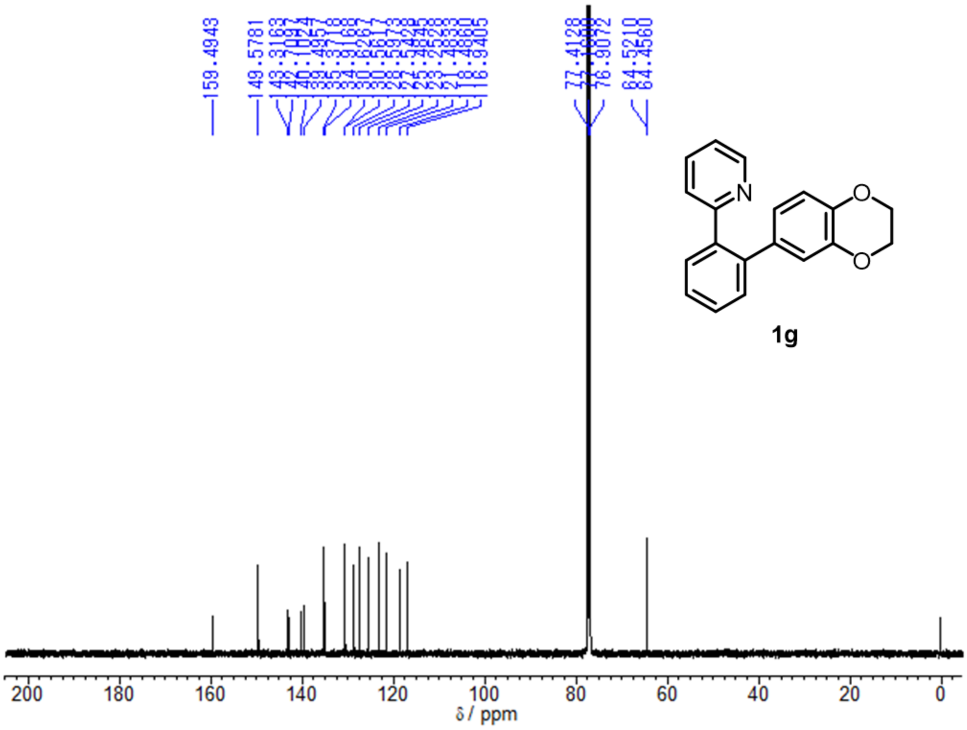

Figure S31. ^{13}C NMR spectrum (125.76 MHz, $CDCl_3$) of **1g**.

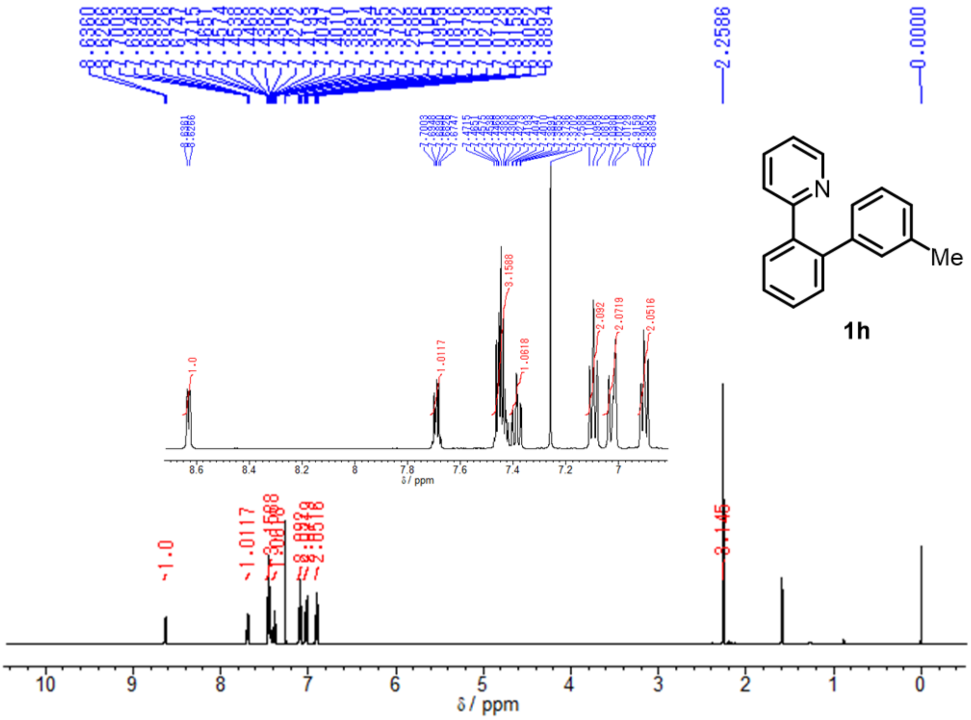

Figure S32. ${}^{1}H$ NMR spectrum (500.13 MHz, $CDCl_3$) of **1h**.

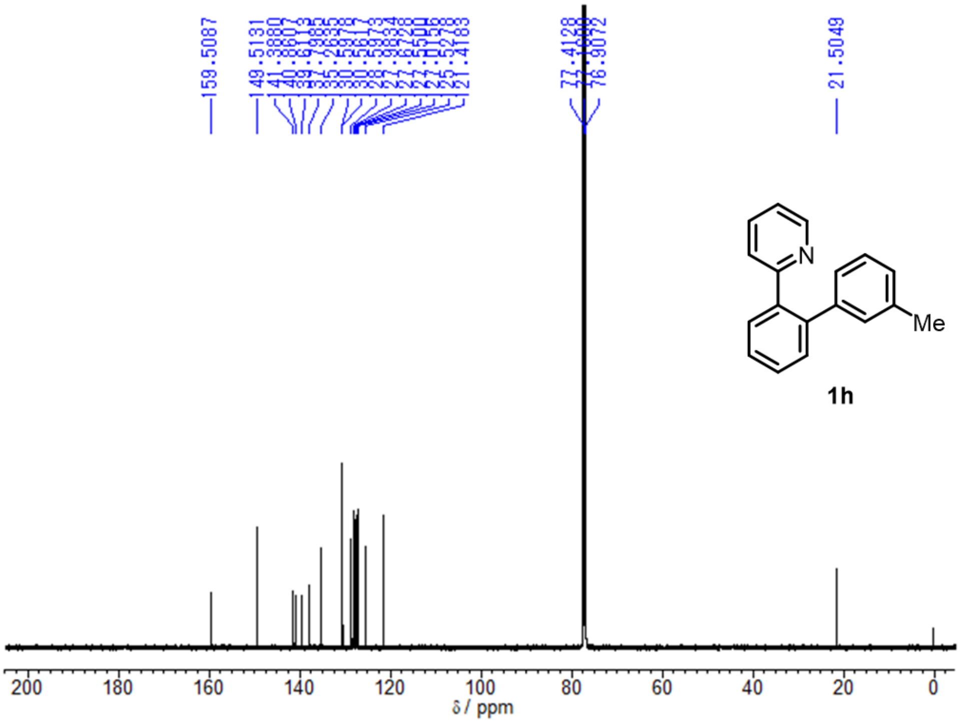

Figure S33. ${}^{13}C$ NMR spectrum (125.76 MHz, $CDCl_3$) of **1h**.

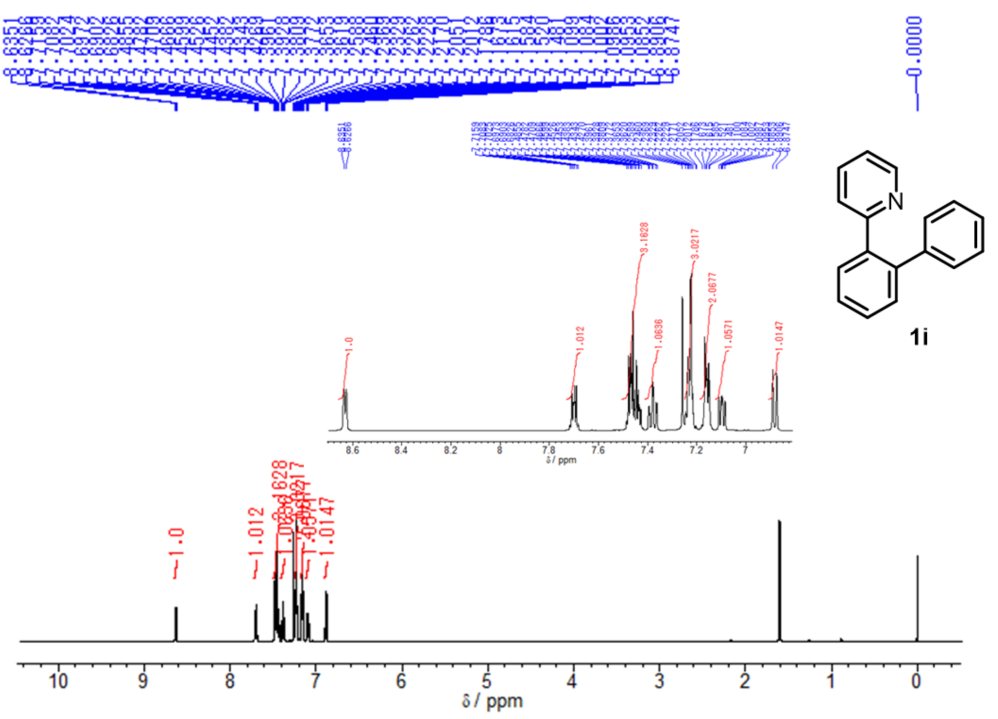

Figure S34. ^{1}H NMR spectrum (500.13 MHz, $CDCl_3$) of **1i**.

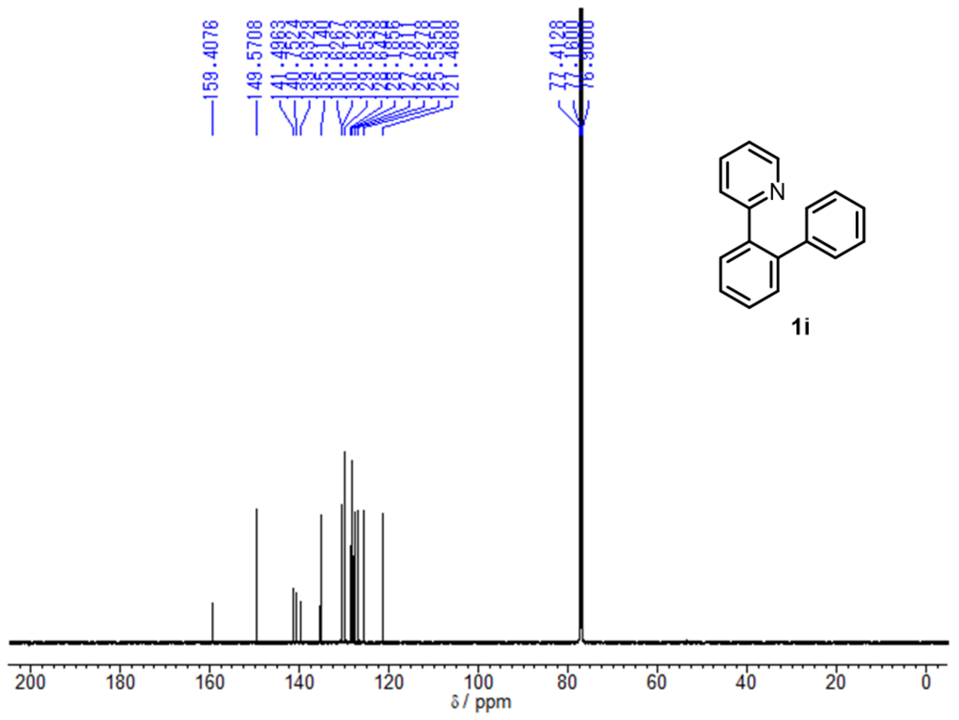

Figure S35. ^{13}C NMR spectrum (125.76 MHz, $CDCl_3$) of **1i**.

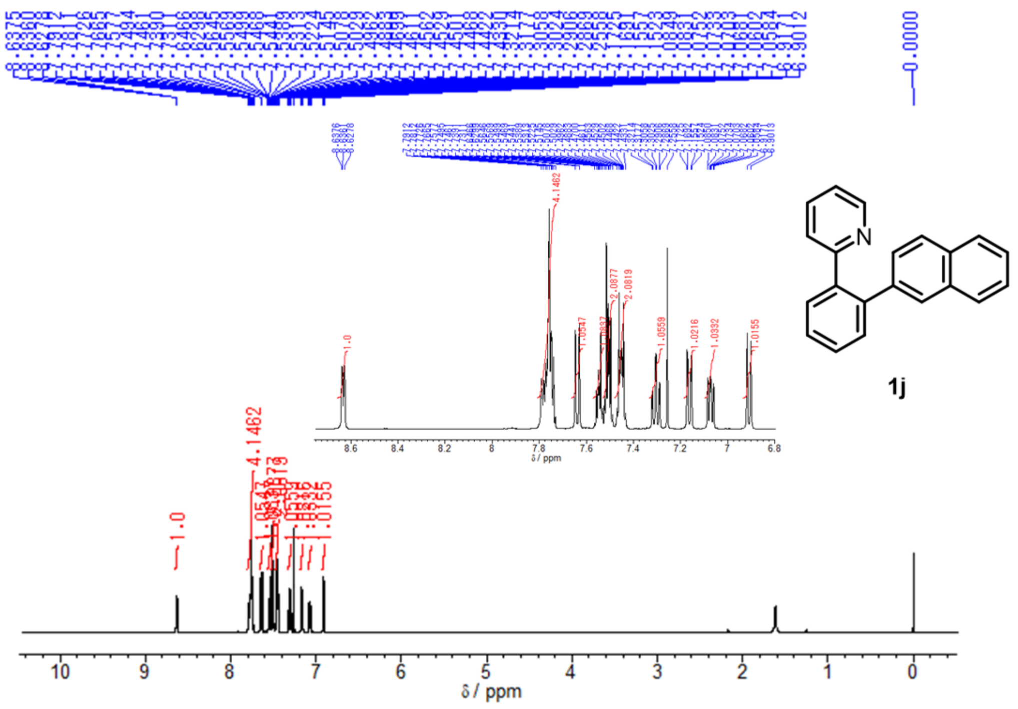

Figure S36. ^{1}H NMR spectrum (500.13 MHz, $CDCl_3$) of **1j**.

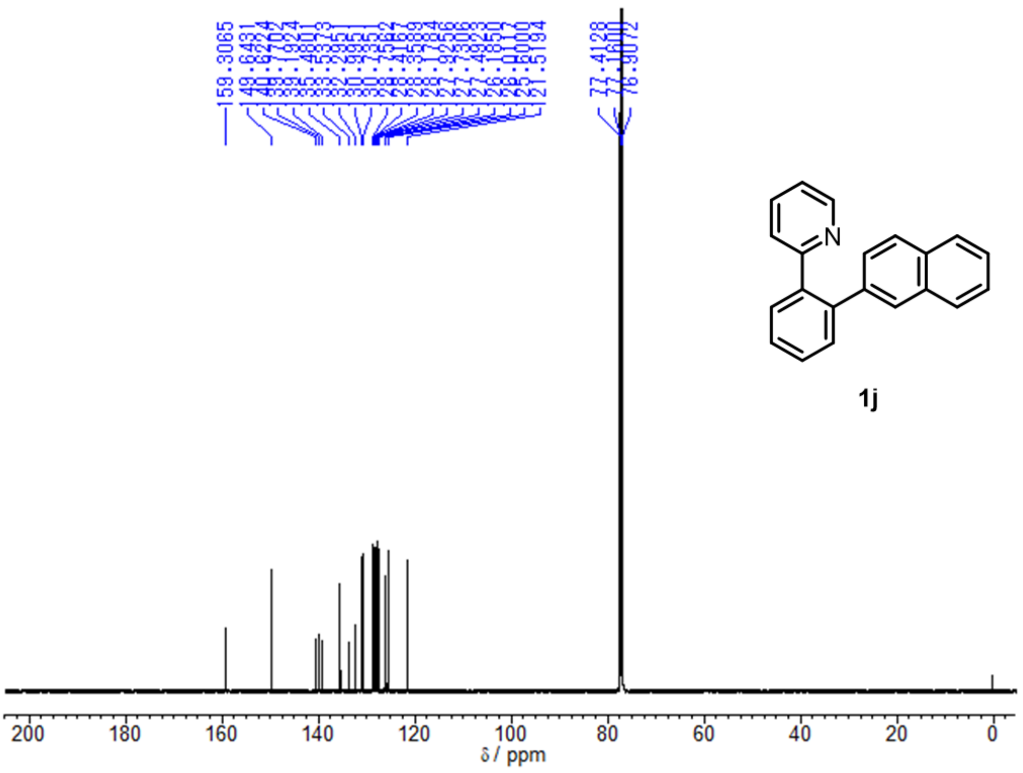

Figure S37. ^{13}C NMR spectrum (125.76 MHz, $CDCl_3$) of **1j**.

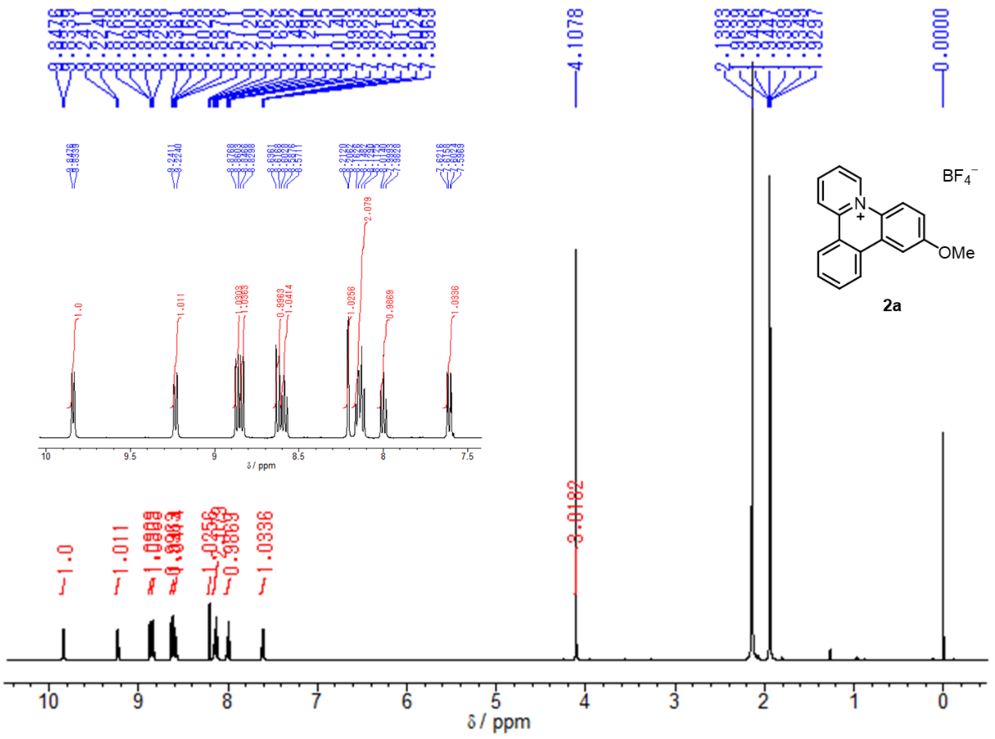

Figure S38. ^{1}H NMR spectrum (500.13 MHz, MeCN-d3) of **2a**.

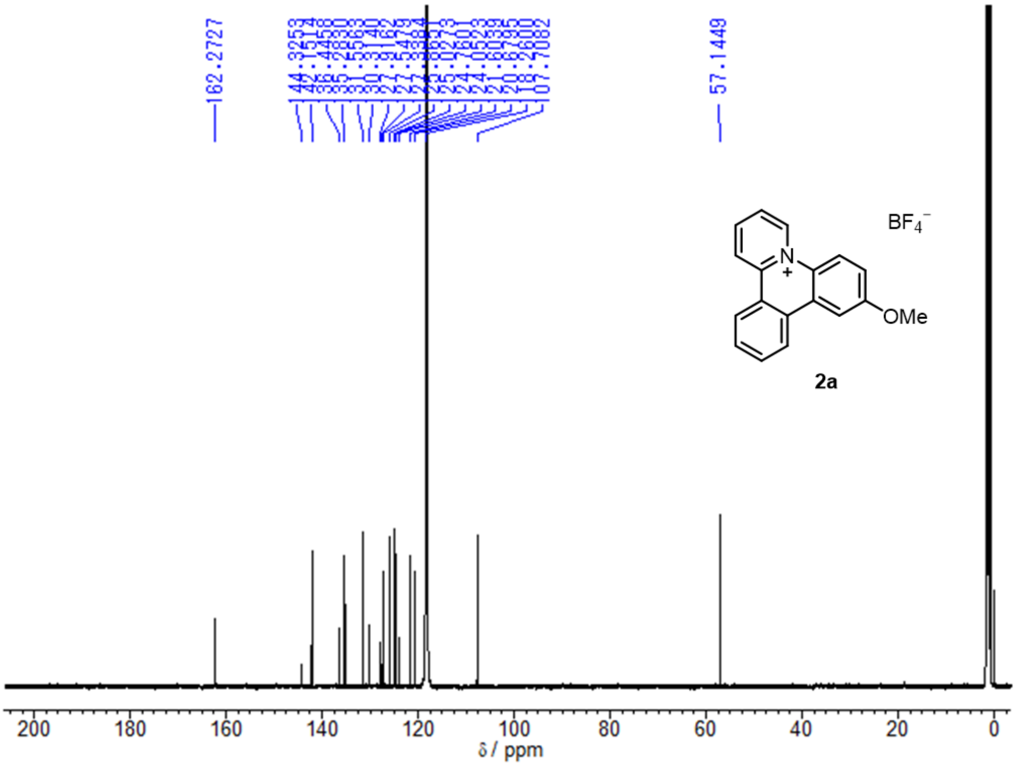

Figure S39. ^{13}C NMR spectrum (125.76 MHz, MeCN-d3) of **2a**.

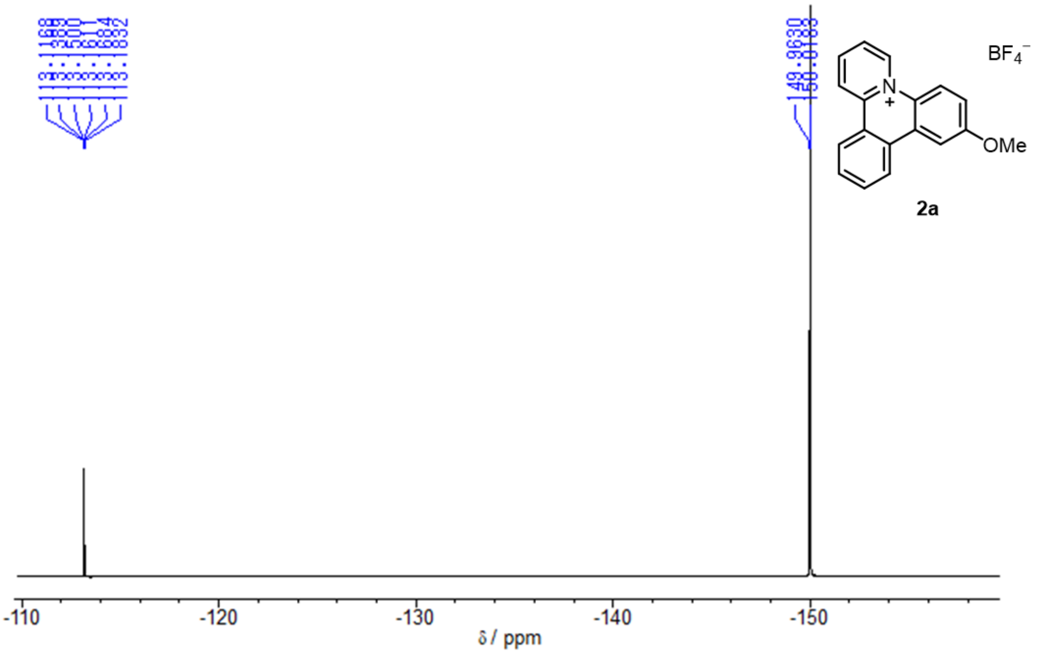

Figure S40. ^{19}F NMR spectrum (470.59 MHz, MeCN-d3) of **2a**.

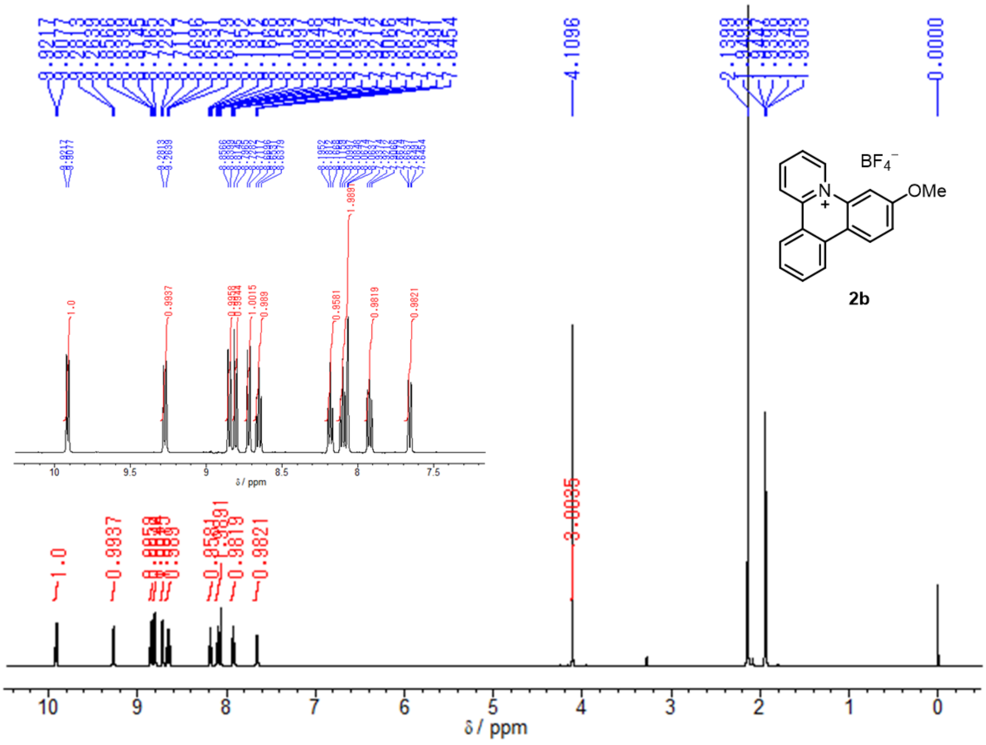

Figure S41. ^{1}H NMR spectrum (500.13 MHz, MeCN-d3) of **2b**.

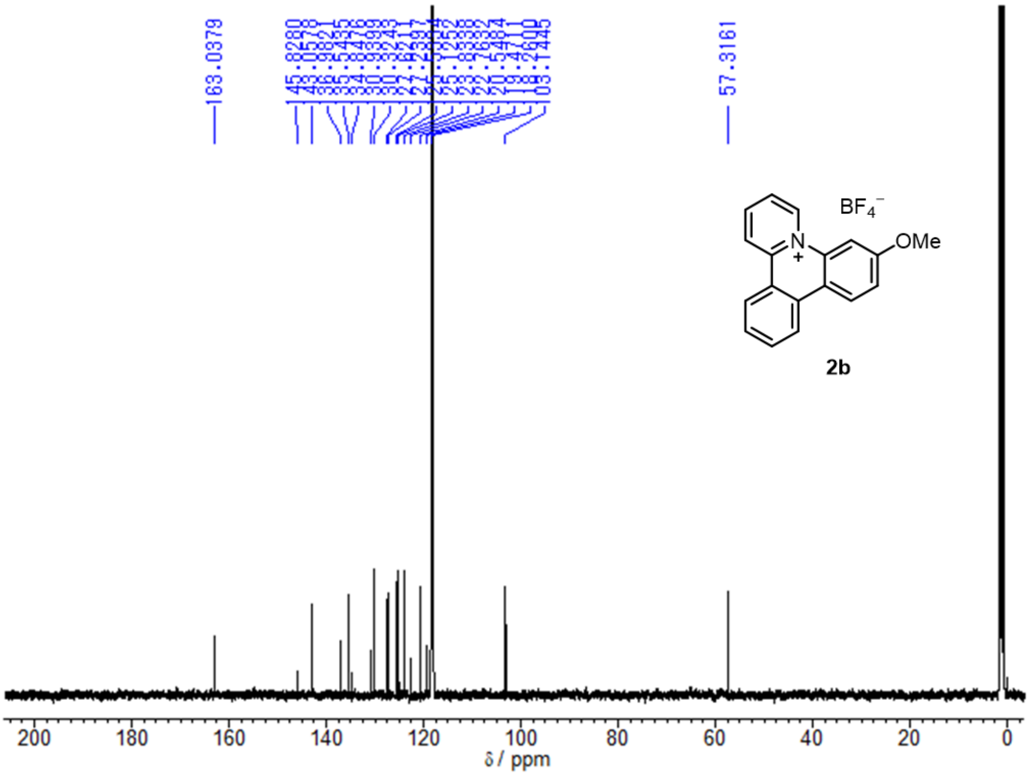

Figure S42. ^{13}C NMR spectrum (125.76 MHz, MeCN-d3) of **2b**.

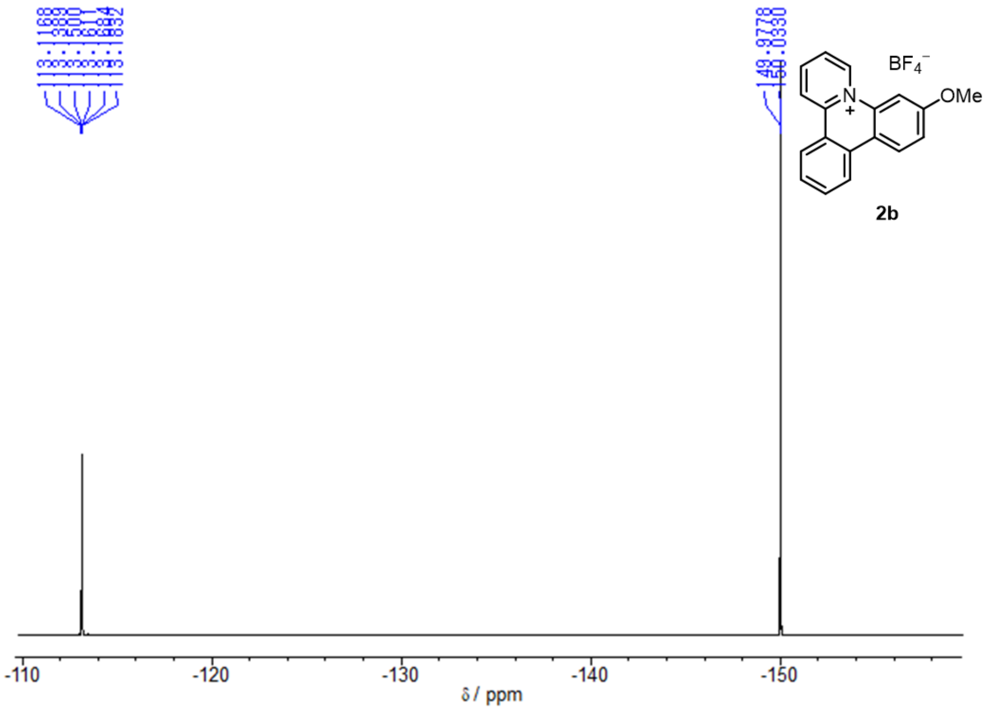

Figure S43. ^{19}F NMR spectrum (470.59 MHz, MeCN-d3) of **2b**.

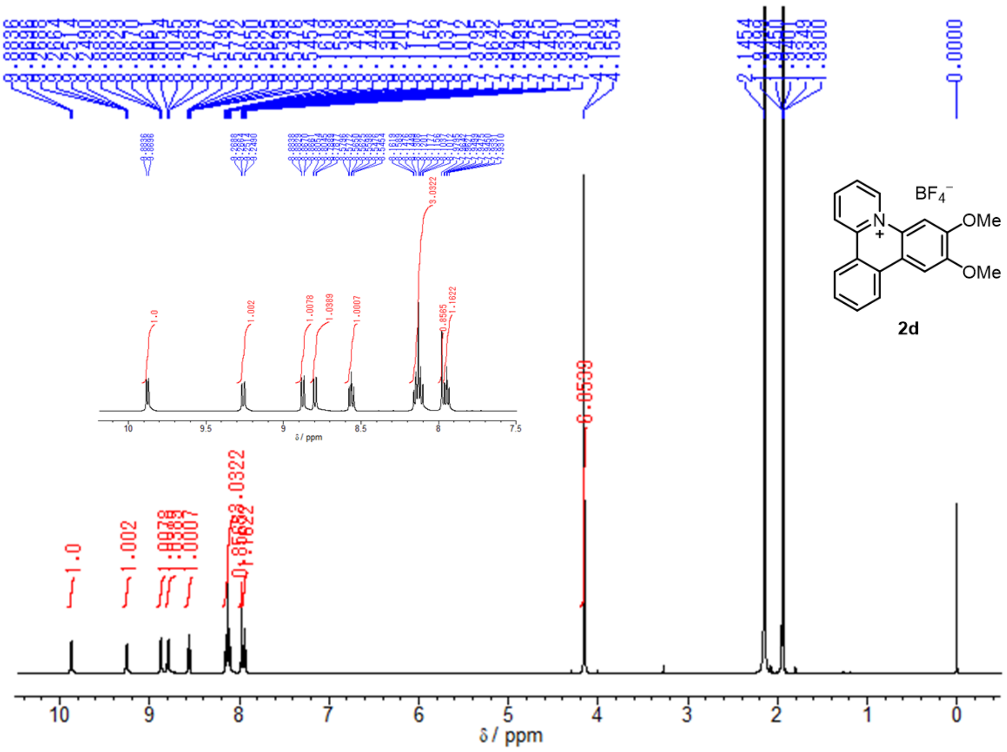

Figure S44. ^{1}H NMR spectrum (500.13 MHz, MeCN-d_3) of **2d**.

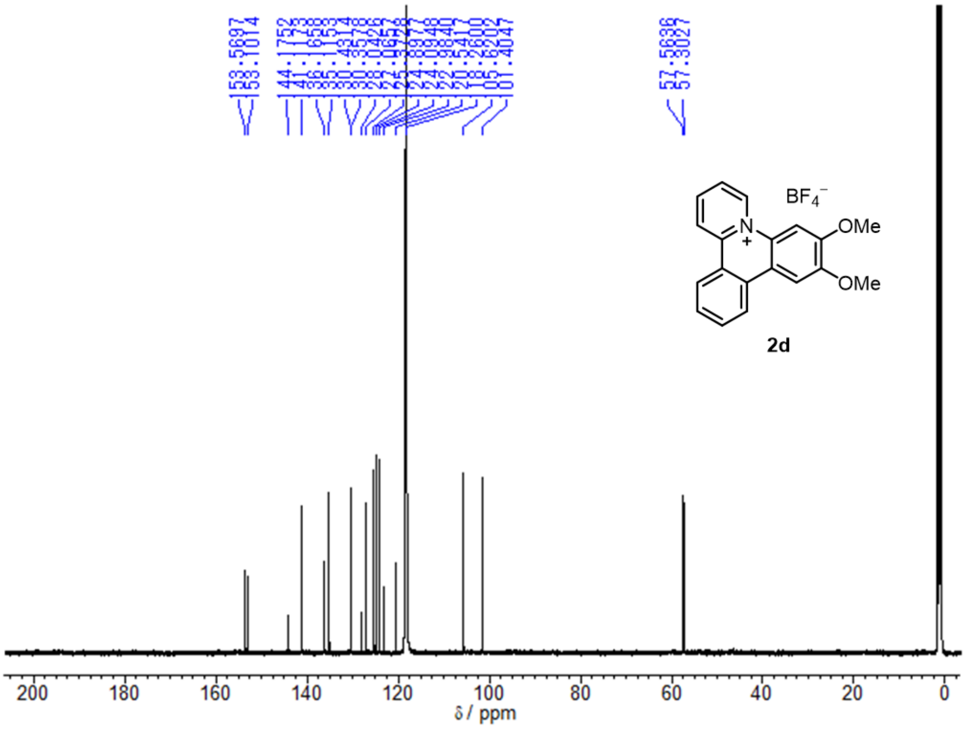

Figure S45. ^{13}C NMR spectrum (125.76 MHz, MeCN-d_3) of **2d**.

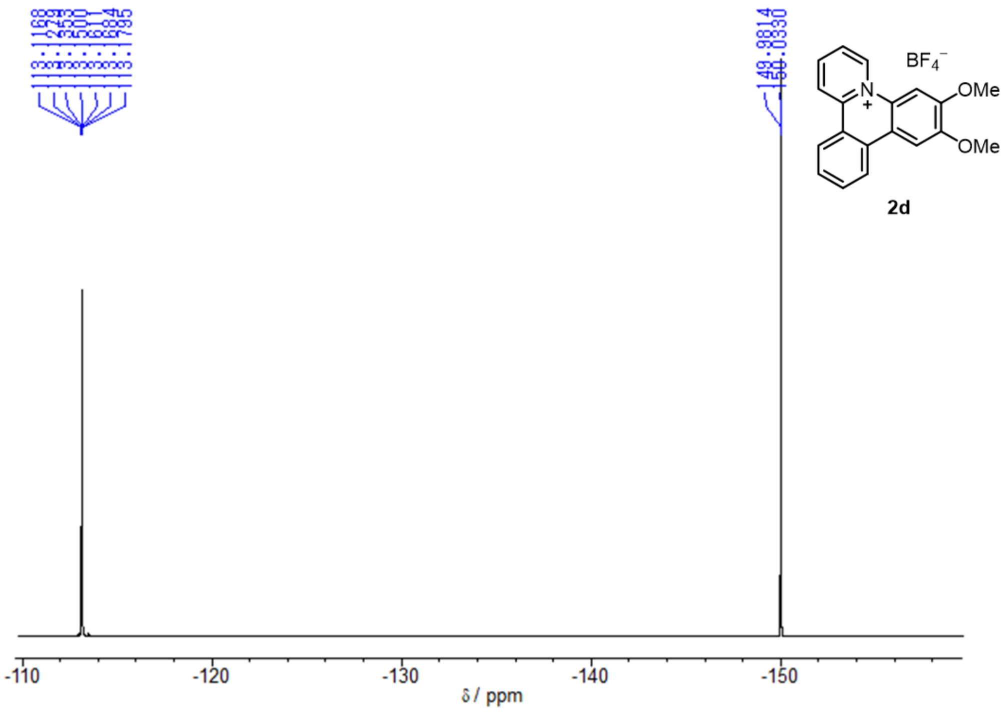

Figure S46. ^{19}F NMR spectrum (470.59 MHz, MeCN-d3) of **2d**.

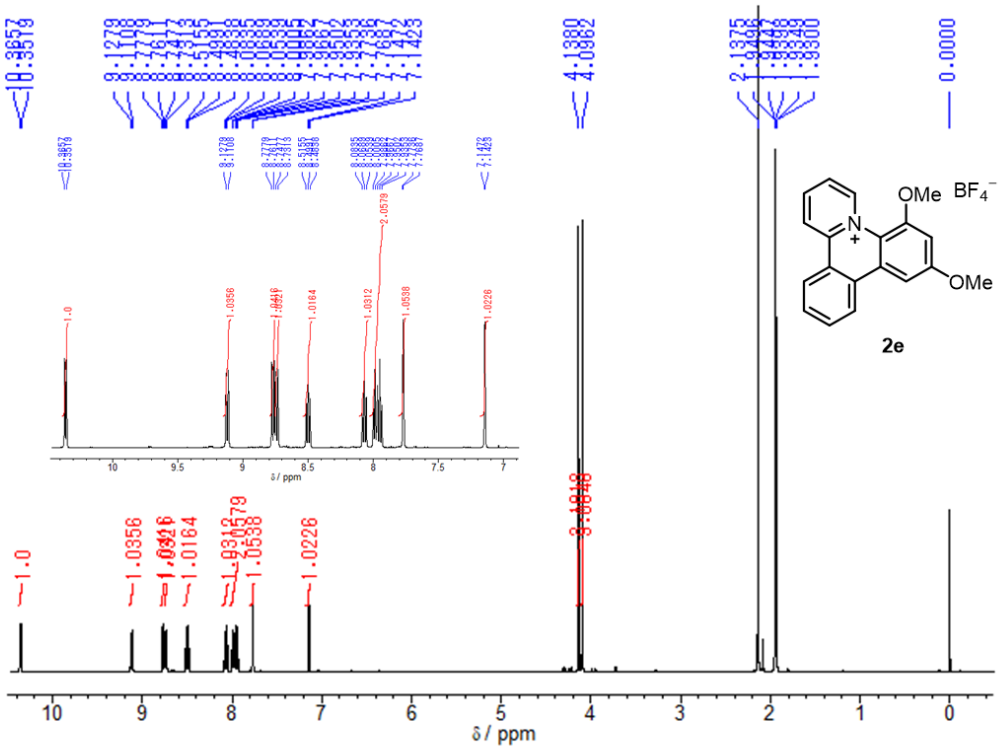

Figure S47. ^{1}H NMR spectrum (500.13 MHz, MeCN-d3) of **2e**.

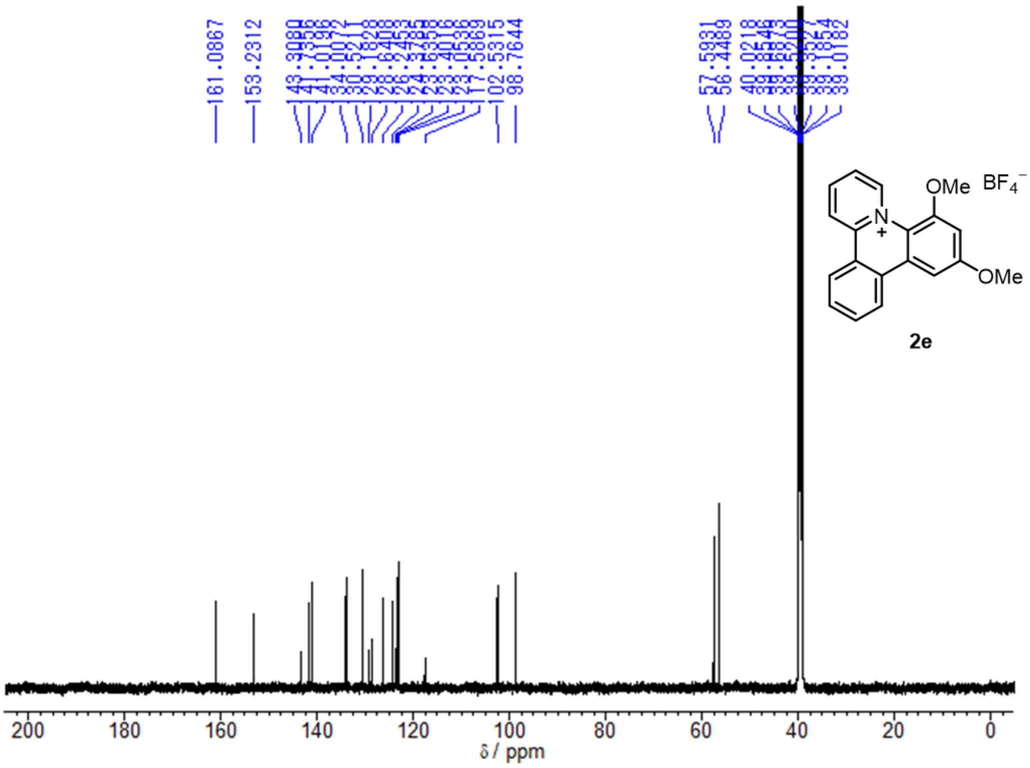

Figure S48. ^{13}C NMR spectrum (125.76 MHz, DMSO-d_6) of **2e**.

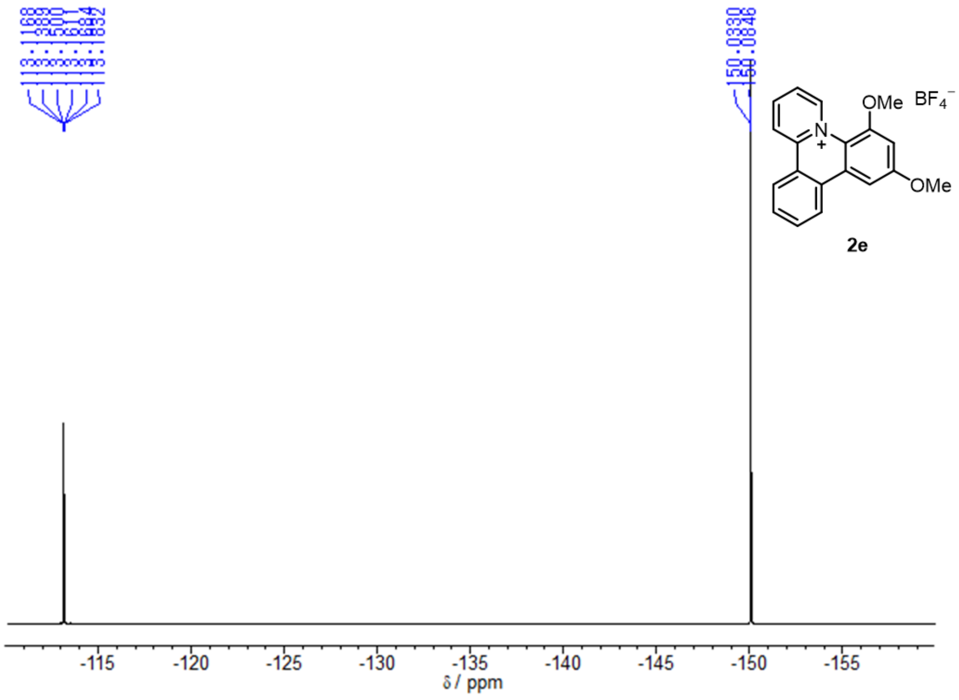

Figure S49. ^{19}F NMR spectrum (470.59 MHz, MeCN-d_3) of **2e**.

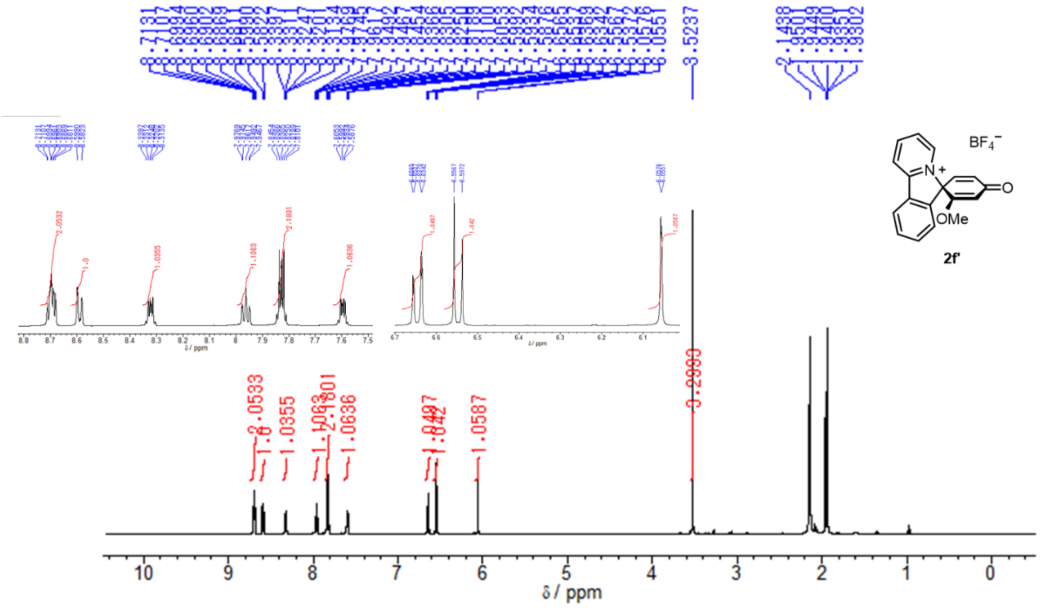

Figure S50. ^{1}H NMR spectrum (500.13 MHz, MeCN-d$_3$) of **2f'**.

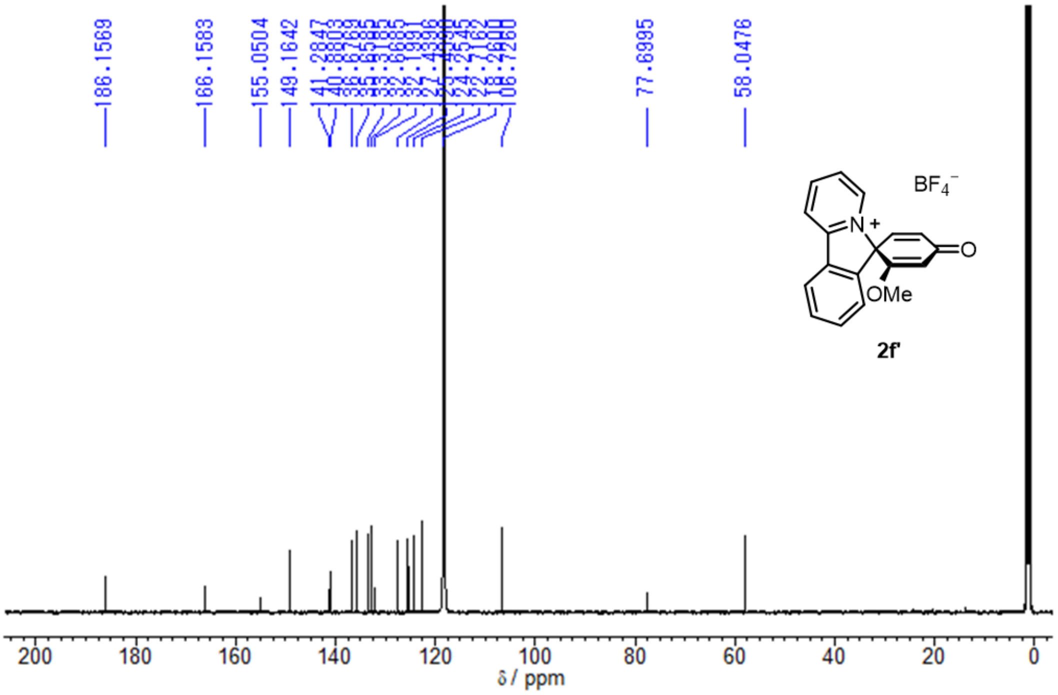

Figure S51. ^{13}C NMR spectrum (125.76 MHz, MeCN-d$_3$) of **2f'**.

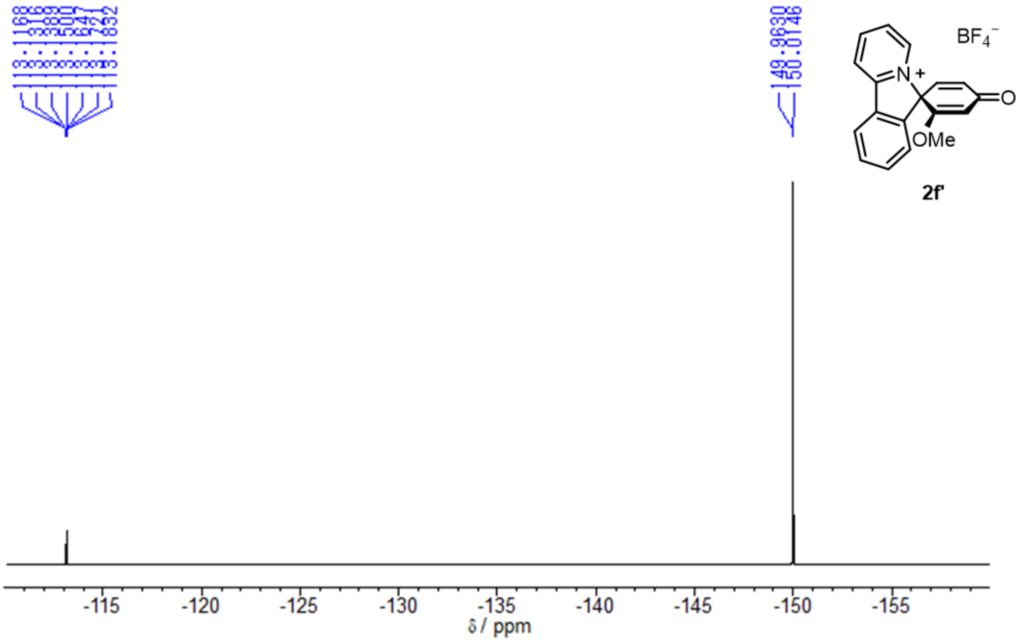

Figure S52. ^{19}F NMR spectrum (470.59 MHz, MeCN-d3) of **2f'**.

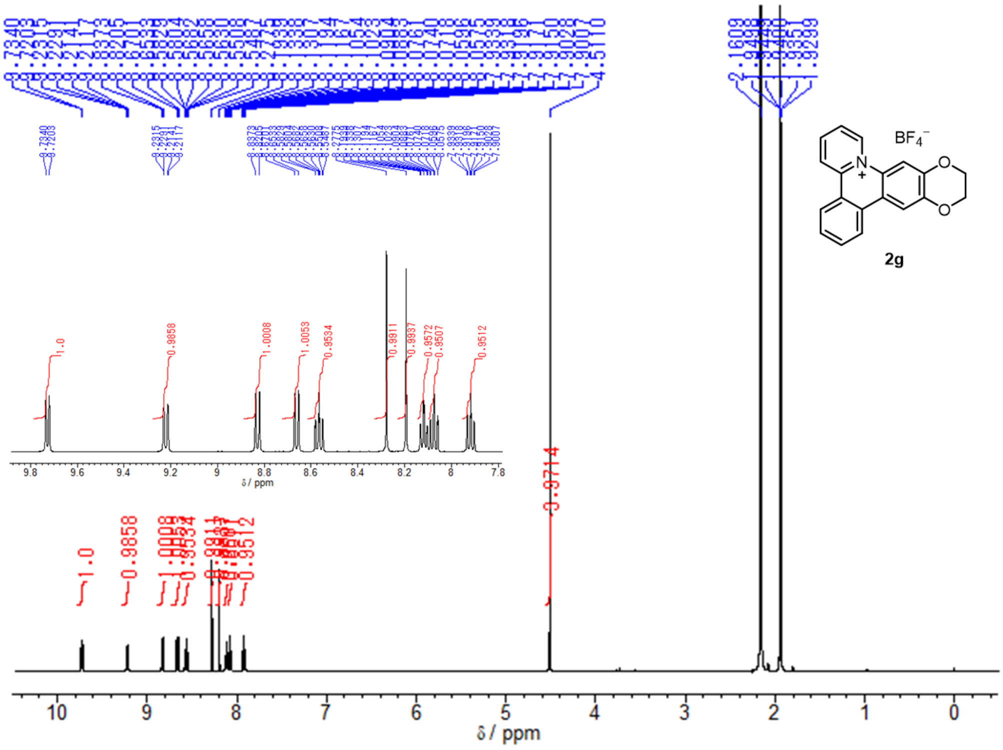

Figure S53. ^{1}H NMR spectrum (500.13 MHz, MeCN-d3) of **2g**.

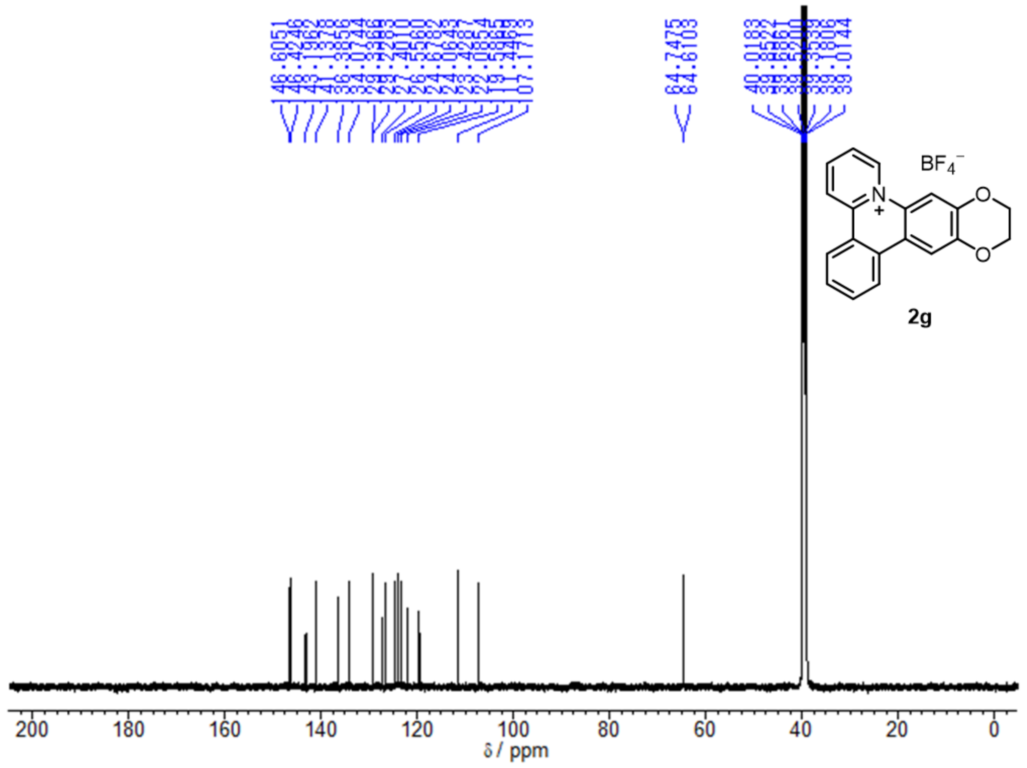

Figure S54. ^{13}C NMR spectrum (125.76 MHz, DMSO-d_6) of **2g**.

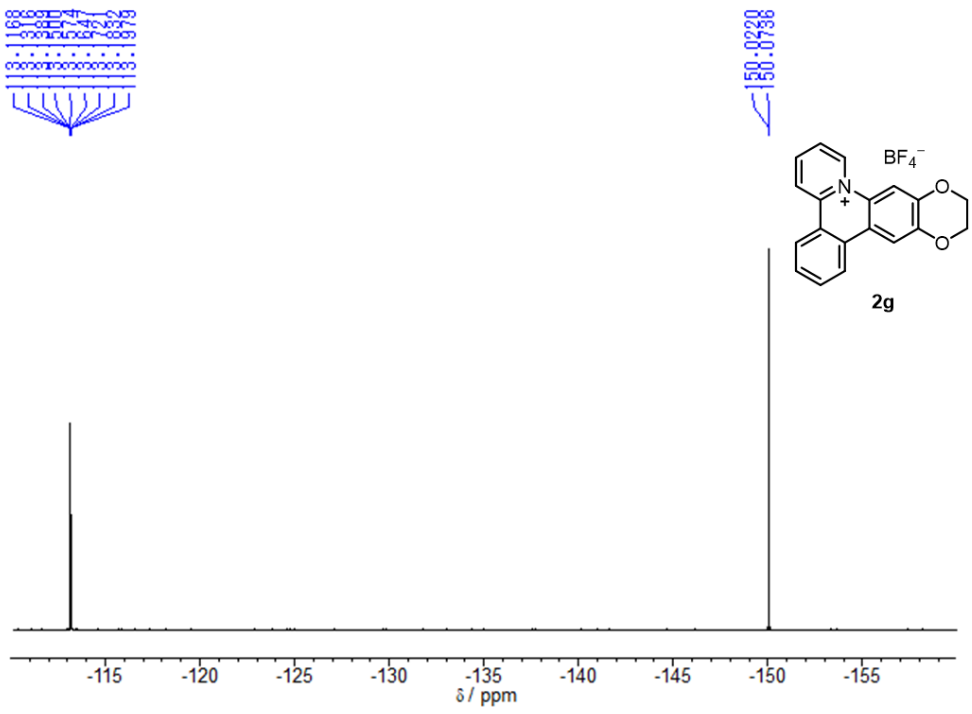

Figure S55. ^{19}F NMR spectrum (470.59 MHz, MeCN-d_3) of **2g**.

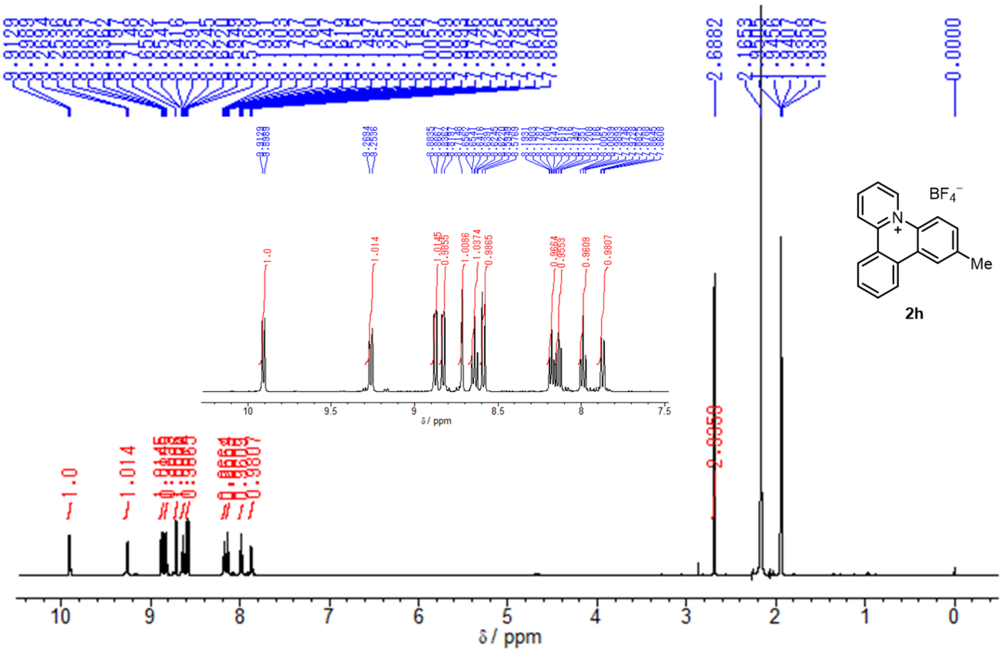

Figure S56. ^{1}H NMR spectrum (500.13 MHz, MeCN-d$_3$) of **2h**.

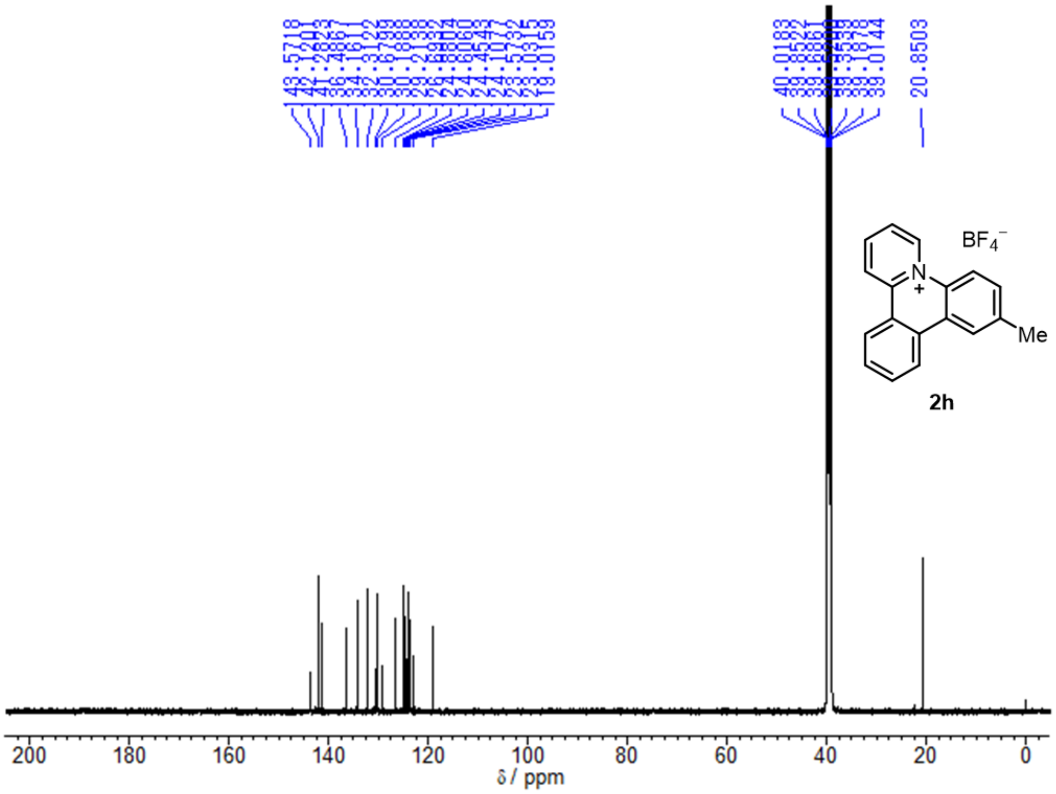

Figure S57. ^{13}C NMR spectrum (125.76 MHz, DMSO-d$_6$) of **2h**.

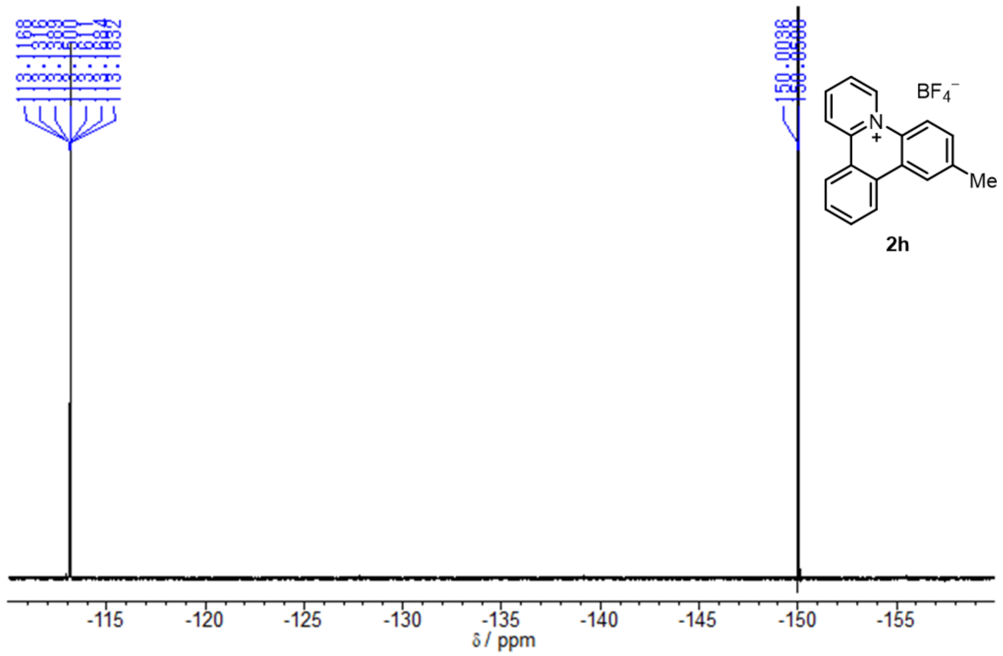

Figure S58. ^{19}F NMR spectrum (470.59 MHz, MeCN-d3) of **2h**.

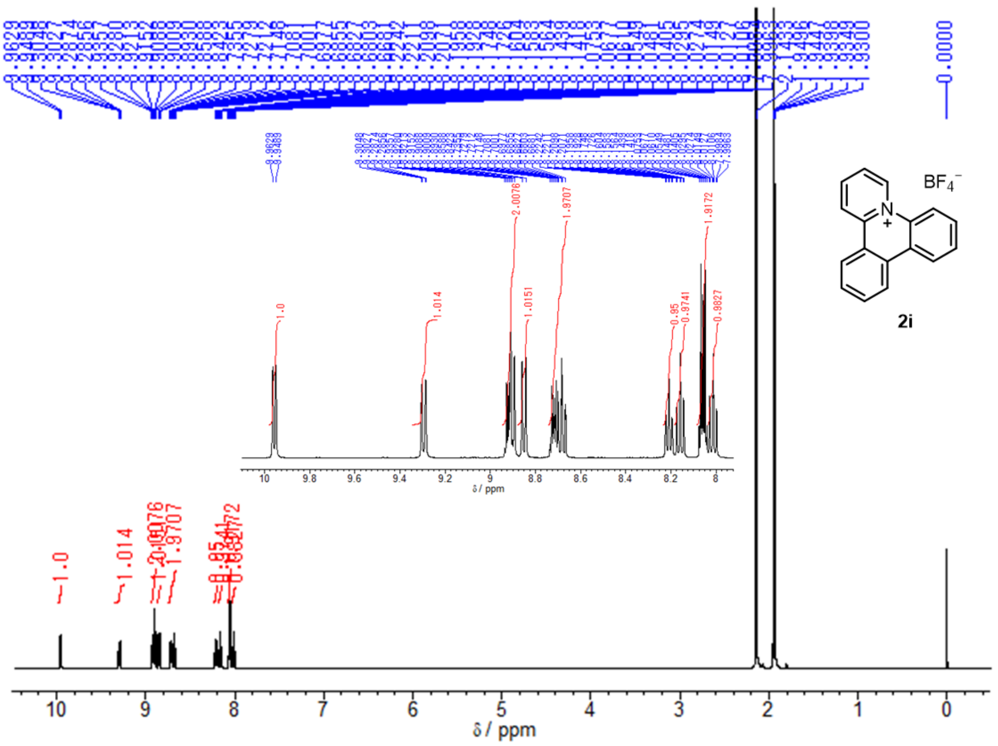

Figure S59. ^{1}H NMR spectrum (500.13 MHz, MeCN-d3) of **2i**.

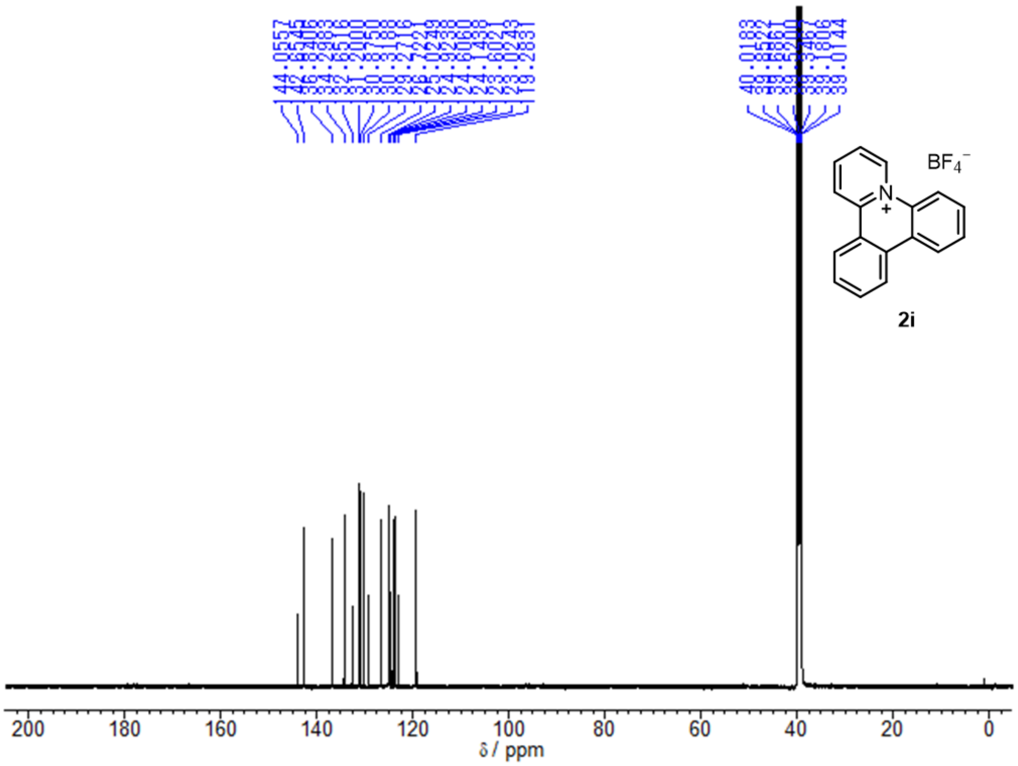

Figure S60. ^{13}C NMR spectrum (125.76 MHz, DMSO-d$_6$) of **2i**.

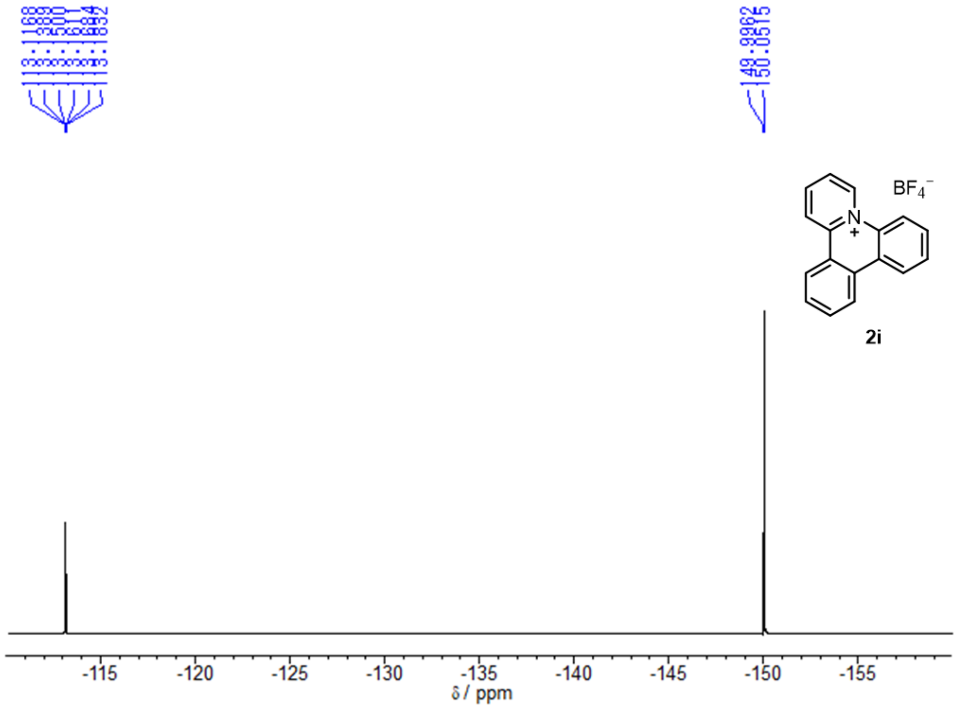

Figure S61. ^{19}F NMR spectrum (470.59 MHz, MeCN-d$_3$) of **2i**.

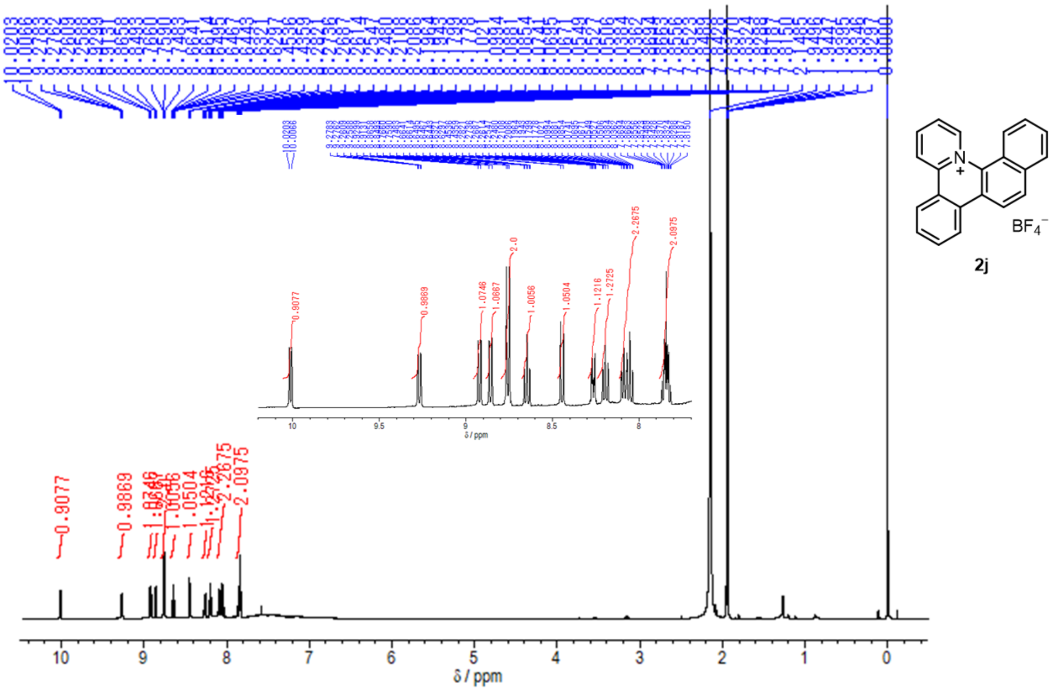

Figure S62. ^{1}H NMR spectrum (500.13 MHz, MeCN-d3) of **2j**.

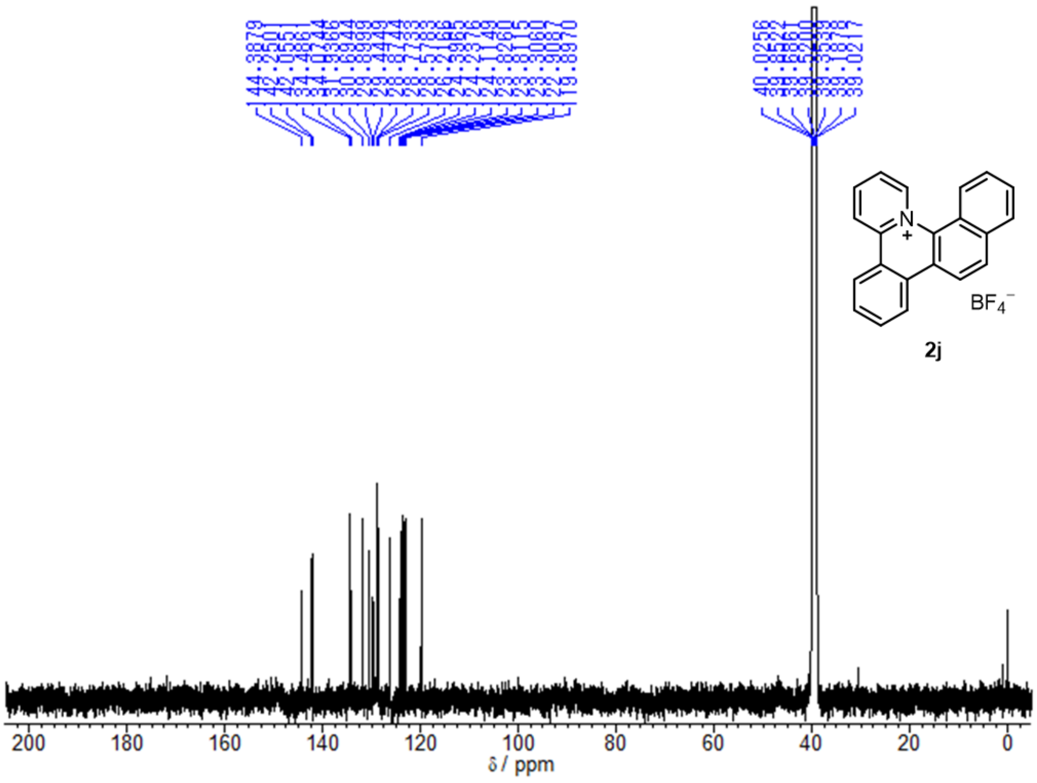

Figure S63. ^{13}C NMR spectrum (125.76 MHz, DMSO-d6) of **2j**.

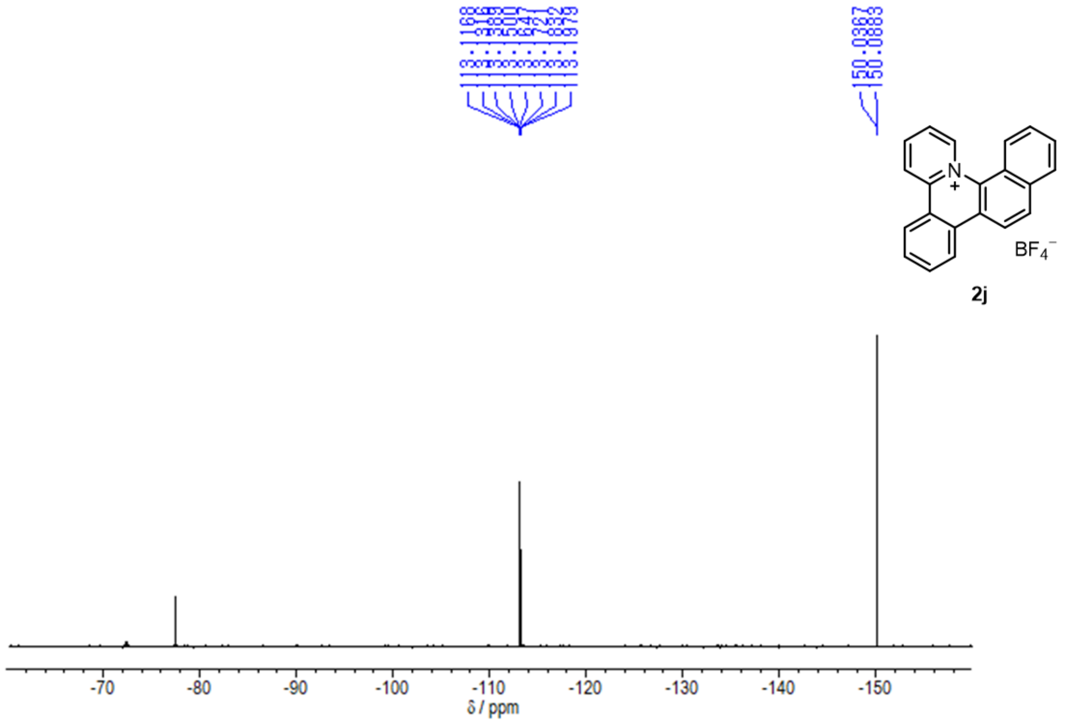

Figure S64. ^{19}F NMR spectrum (470.59 MHz, MeCN-d3) of **2j**.

9. Cartesian coordinates for simulated structures

Table S5. Cartesian coordinates of the optimized structure for **1a** in Figure S1.

Symbol	X	Y	Z
C	2.800431	-3.01049	-0.24055
C	1.558765	-3.607157	-0.030193
C	0.435103	-2.810322	0.164923
C	0.522037	-1.412671	0.146156
C	1.773728	-0.812675	-0.089846
C	2.900187	-1.623499	-0.267874
H	3.685374	-3.621063	-0.388603
H	1.466051	-4.688286	-0.006926
H	-0.528106	-3.273296	0.358589
H	3.860857	-1.150362	-0.446058
C	1.945155	0.66576	-0.200891
C	1.142537	1.42172	-1.062831
C	1.352489	2.79097	-1.153904
H	0.368127	0.937466	-1.647308
C	3.117332	2.537307	0.433587
C	2.359731	3.368387	-0.384581
H	0.742696	3.39702	-1.816013
H	3.917318	2.948074	1.044181
H	2.5595	4.433431	-0.41896
C	-0.705958	-0.613399	0.421093
C	-0.72963	0.330573	1.458779
C	-1.852919	-0.817346	-0.340912
C	-1.888349	1.053022	1.706796
H	0.156353	0.491873	2.064592
C	-3.018142	-0.083295	-0.088633
H	-1.855897	-1.536425	-1.154553
C	-3.040376	0.86066	0.939696
H	-1.907355	1.781409	2.511562
H	-3.928912	1.441469	1.155083
N	2.925797	1.216326	0.529547
O	-4.072226	-0.358615	-0.901321
C	-5.279672	0.360843	-0.702306
H	-5.127834	1.436097	-0.846816
H	-5.975967	-0.010675	-1.45292
H	-5.689666	0.177572	0.29685

Table S6. Cartesian coordinates of the optimized structure for the radical cation of **1a** in Figure S2.

Symbol	X	Y	Z
C	-2.797258	3.030057	-0.166252
C	-1.548388	3.620978	0.018459
C	-0.421536	2.820781	0.16328
C	-0.525149	1.424547	0.120719
C	-1.784042	0.826878	-0.086795
C	-2.909416	1.644667	-0.216251
H	-3.682635	3.647453	-0.275749
H	-1.453089	4.700548	0.062555
H	0.54581	3.278453	0.345771
H	-3.876881	1.180034	-0.375237
C	-1.950697	-0.648903	-0.213793
C	-1.140211	-1.397939	-1.07491
C	-1.331169	-2.77365	-1.153871
H	-0.387106	-0.908915	-1.682963
C	-3.099888	-2.527807	0.432498
C	-2.327222	-3.356168	-0.378946
H	-0.715743	-3.375175	-1.814268
H	-3.895606	-2.945484	1.043374
H	-2.512688	-4.423794	-0.403694
C	0.694629	0.613189	0.34244
C	0.675951	-0.46752	1.282951
C	1.870578	0.895395	-0.301879
C	1.81898	-1.254608	1.527217
H	-0.231509	-0.666863	1.842301
C	3.02999	0.104895	-0.051013
H	1.949136	1.692794	-1.031908
C	2.994675	-0.989322	0.876724
H	1.763097	-2.06624	2.242292
H	3.878545	-1.585309	1.063542
N	-2.925583	-1.2064	0.520824
O	4.087575	0.441457	-0.730986
C	5.333095	-0.282509	-0.618806
H	5.184519	-1.318408	-0.925571
H	6.008755	0.222833	-1.302544
H	5.706347	-0.216556	0.403576

Table S7. Cartesian coordinates of the optimized structure for the radical cation of **1b** in Figure S2.

Symbol	X	Y	Z
C	-3.702572	-2.072168	-0.289973
C	-2.739954	-3.074847	-0.14995
C	-1.4089	-2.731177	0.009505
C	-1.005447	-1.377845	0.012014
C	-1.996023	-0.363737	-0.080381
C	-3.329487	-0.731744	-0.247646
H	-4.747498	-2.335763	-0.413773
H	-3.028721	-4.119471	-0.178296
H	-0.660084	-3.513656	0.067715
H	-4.081909	0.046494	-0.314583
C	-1.674213	1.078386	0.095978
C	-0.896573	1.512619	1.174858
C	-0.629107	2.871177	1.304504
H	-0.523387	0.802239	1.904537
C	-1.933247	3.22482	-0.667339
C	-1.149864	3.748127	0.360099
H	-0.029119	3.235327	2.13147
H	-2.365926	3.881555	-1.416972
H	-0.966372	4.814862	0.416601
C	0.418454	-1.081065	0.026071
C	0.963831	-0.037408	-0.789609
C	1.317225	-1.864206	0.809154
C	2.303649	0.229222	-0.815434
H	0.303336	0.526112	-1.437678
C	2.653233	-1.592866	0.819125
H	0.928249	-2.651129	1.444656
C	3.17266	-0.53863	0.005989
H	2.689178	1.004737	-1.464054
H	3.345365	-2.151113	1.438299
N	-2.199709	1.92362	-0.803725
O	4.466087	-0.362995	0.082297
C	5.122311	0.666013	-0.683744
H	6.171881	0.5911	-0.413681
H	4.99078	0.474785	-1.749912
H	4.727652	1.644505	-0.405938

Table S8. Cartesian coordinates of the optimized structure for the radical cation of **1c** in Figure S2.

Symbol	X	Y	Z
C	1.544011	-0.837559	1.204636
C	1.902803	0.091787	0.223478
C	3.860645	-1.022427	-0.207215
C	3.577764	-2.007926	0.735253
C	2.395411	-1.90576	1.460725
H	0.623723	-0.71599	1.765756
H	4.779527	-1.062562	-0.785729
H	4.272519	-2.823961	0.897494
H	2.141636	-2.640647	2.217233
C	1.028309	1.259774	-0.080405
C	-0.356408	1.119774	-0.335222
C	1.582249	2.538374	-0.037502
C	-1.149436	2.272215	-0.496255
C	0.791258	3.668569	-0.224908
H	2.642793	2.64169	0.165702
C	-0.578761	3.534949	-0.449767
H	-2.207579	2.170236	-0.707716
H	1.242649	4.654168	-0.183974
H	-1.198744	4.412262	-0.597082
C	-0.961217	-0.196967	-0.554897
C	-2.211042	-0.563935	0.095287
C	-0.380877	-1.134381	-1.418367
C	-2.844287	-1.80138	-0.181361
C	-1.002602	-2.340453	-1.672278
H	0.545282	-0.884864	-1.92235
C	-2.239805	-2.670814	-1.046801
H	-3.780896	-2.059036	0.294459
H	-0.558897	-3.041014	-2.36999
H	-2.705187	-3.625577	-1.262272
N	3.051194	0.009689	-0.463594
O	-2.652833	0.298406	0.968345
C	-3.870584	0.067246	1.705795
H	-3.763602	-0.831799	2.314186
H	-3.983555	0.943851	2.33701
H	-4.709932	-0.018344	1.014482

Table S9. Cartesian coordinates of the optimized structure for the radical cation of **1d** in Figure S2.

Symbol	X	Y	Z
C	-1.335935	1.359934	-1.400393
C	-2.066685	1.020728	-0.257535
C	-2.271334	3.226875	0.34
C	-1.537675	3.65738	-0.761157
C	-1.065604	2.698776	-1.653248
H	-0.99339	0.585738	-2.078568
H	-2.664418	3.94574	1.053854
H	-1.350719	4.714063	-0.914611
H	-0.501054	2.988346	-2.533343
C	-2.394615	-0.40327	0.046285
C	-3.736221	-0.75233	0.219186
C	-1.413149	-1.413036	0.105738
C	-4.114133	-2.074964	0.427062
H	-4.488471	0.028277	0.171074
C	-1.807033	-2.745656	0.293315
C	-3.146315	-3.077239	0.457103
H	-5.162588	-2.323463	0.554006
H	-1.05248	-3.524595	0.349423
H	-3.430989	-4.111861	0.615336
C	0.034639	-1.115077	0.036466
C	0.602056	-0.114725	0.792671
C	0.877707	-1.90158	-0.818991
C	1.980407	0.156225	0.727197
H	0.010569	0.489258	1.470666
C	2.222483	-1.663733	-0.924516
H	0.430417	-2.688915	-1.41568
C	2.814887	-0.633426	-0.167841
H	2.827364	-2.2617	-1.592939
N	-2.537652	1.941045	0.59553
O	2.396948	1.126305	1.502408
O	4.084831	-0.317946	-0.208134
C	3.760178	1.56922	1.673806
H	4.144124	1.971096	0.73733
H	3.688691	2.348489	2.4282
H	4.380519	0.745813	2.025584
C	4.995497	-1.028956	-1.068555
H	4.690194	-0.906698	-2.108955
H	5.96161	-0.562332	-0.898331
H	5.025028	-2.08232	-0.785505

Table S10. Cartesian coordinates of the optimized structure for the radical cation of **1e** in Figure S2.

Symbol	X	Y	Z
C	-3.763631	-2.461876	-0.458682
C	-2.674333	-3.299197	-0.689986
C	-1.382906	-2.790655	-0.60291
C	-1.163598	-1.448046	-0.27652
C	-2.26127	-0.605315	-0.023302
C	-3.55364	-1.126588	-0.130291
H	-4.774991	-2.848263	-0.529572
H	-2.827684	-4.341399	-0.948902
H	-0.534576	-3.433724	-0.816966
H	-4.397712	-0.472469	0.062701
C	-2.09603	0.820364	0.386393
C	-1.284178	1.171489	1.470242
C	-1.164507	2.512331	1.816177
H	-0.765466	0.404853	2.035689
C	-2.660187	3.015966	0.023712
C	-1.861985	3.460094	1.07409
H	-0.542079	2.809359	2.653666
H	-3.228126	3.725025	-0.572588
H	-1.798682	4.517594	1.303616
C	0.233366	-0.93838	-0.249991
C	1.221858	-1.585459	0.471342
C	0.595262	0.200148	-0.973383
C	2.575894	-1.086494	0.484169
H	1.010096	-2.469124	1.063037
C	1.948167	0.699363	-0.946334
H	-0.122172	0.739298	-1.582183
C	2.944577	0.059648	-0.218542
H	3.958203	0.431268	-0.198498
O	2.115897	1.783589	-1.667762
O	3.388744	-1.805912	1.219746
C	3.412556	2.397382	-1.750744
H	3.279228	3.258952	-2.399187
H	3.737707	2.716529	-0.758118
H	4.12691	1.698132	-2.190787
C	4.770164	-1.428647	1.349721
H	4.842274	-0.437435	1.802092
H	5.208957	-2.176198	2.004676
H	5.254279	-1.449594	0.371274
N	-2.782395	1.729859	-0.32097

Table S11. Cartesian coordinates of the optimized structure for the radical cation of **1f** in Figure S2.

Symbol	X	Y	Z
C	-1.431772	1.343223	1.265086
C	-2.17938	0.83123	0.199846
C	-3.129907	2.879758	-0.200805
C	-2.409681	3.482873	0.827626
C	-1.547793	2.692102	1.579977
H	-0.785037	0.69255	1.843864
H	-3.820886	3.463217	-0.803128
H	-2.532466	4.540024	1.03323
H	-0.979491	3.114627	2.401729
C	-2.092947	-0.609063	-0.171152
C	-0.852198	-1.266636	-0.346419
C	-3.272101	-1.346633	-0.271952
C	-0.83476	-2.656594	-0.571255
C	-3.240799	-2.714931	-0.524034
H	-4.219983	-0.838803	-0.129245
C	-2.018565	-3.37184	-0.668266
H	0.112208	-3.163229	-0.715546
H	-4.169628	-3.270874	-0.595462
H	-1.988956	-4.438047	-0.863893
C	0.401217	-0.514154	-0.421556
C	1.591657	-0.946944	0.295005
C	0.505282	0.639113	-1.233003
C	2.801545	-0.277807	0.162871
C	1.691384	1.301046	-1.374049
H	-0.365319	0.971756	-1.785383
C	2.855963	0.841354	-0.669707
H	3.67438	-0.605427	0.707407
H	1.791739	2.164221	-2.020901
N	-3.027778	1.584952	-0.514655
O	1.406459	-1.977582	1.09357
O	3.934562	1.560964	-0.88061
C	2.496653	-2.495742	1.867812
H	2.859901	-1.732558	2.560029
H	2.080373	-3.334766	2.419082
H	3.297978	-2.837235	1.208698
C	5.176381	1.206335	-0.251295
H	5.483935	0.207579	-0.568556
H	5.892414	1.947426	-0.596159
H	5.074047	1.257684	0.834759

Table S12. Cartesian coordinates of the optimized structure for the radical cation of **1g** in Figure S2.

Symbol	X	Y	Z
C	2.323092	0.798165	-0.186304
C	1.504848	1.41548	-1.138487
C	1.540189	2.800406	-1.259137
C	2.394175	3.522079	-0.432864
C	3.188759	2.818796	0.470224
H	0.86549	0.819568	-1.780809
H	0.915455	3.303376	-1.989546
H	2.455232	4.602914	-0.487277
H	3.876501	3.34811	1.124113
C	2.319677	-0.682106	-0.019691
C	1.122646	-1.421157	0.099284
C	3.537372	-1.364263	-0.051506
C	1.179348	-2.82372	0.135108
C	3.581659	-2.753494	0.006728
H	4.454093	-0.79164	-0.145142
C	2.39827	-3.486423	0.092028
H	0.260877	-3.391538	0.243949
H	4.538308	-3.264207	-0.024721
H	2.425813	-4.569656	0.137418
C	-0.1892	-0.769282	0.250559
C	-0.341791	0.336148	1.167205
C	-1.294036	-1.234525	-0.447754
C	-1.551314	0.933047	1.372948
H	0.519624	0.667264	1.735176
C	-2.529501	-0.613781	-0.288439
H	-1.209425	-2.03501	-1.173238
C	-2.679014	0.456917	0.673838
H	-1.682967	1.737008	2.086552
O	-3.851824	0.98483	0.930834
O	-3.552448	-1.004398	-1.027893
C	-4.703627	-0.132237	-1.036795
H	-4.483482	0.705893	-1.702967
H	-5.524092	-0.721351	-1.440848
C	-5.010595	0.326295	0.362271
H	-5.265904	-0.505242	1.023275
H	-5.806707	1.067572	0.370315
N	3.164233	1.489343	0.597844

Table S13. Cartesian coordinates of the optimized structure for the radical cation of **1h** in Figure S2.

Symbol	X	Y	Z
C	3.520725	-1.621662	-0.415017
C	2.729249	-2.765134	-0.200279
C	1.383011	-2.630259	0.043691
C	0.770983	-1.346459	0.044381
C	1.606373	-0.1832	-0.085877
C	2.962	-0.350108	-0.352608
H	4.583503	-1.731284	-0.601376
H	3.176857	-3.750992	-0.246608
H	0.766915	-3.515605	0.147043
H	3.586243	0.529009	-0.462086
C	1.094237	1.184118	0.15657
C	0.233816	1.455674	1.228665
C	1.117937	3.386204	-0.475523
C	-0.19829	2.763964	1.424167
H	-0.071071	0.667146	1.906918
C	0.240894	3.748868	0.550322
H	1.489175	4.134877	-1.169506
H	-0.862159	3.003715	2.24729
H	-0.072113	4.780845	0.658017
C	-0.667595	-1.264457	0.069209
C	-1.427098	-2.202341	0.828939
C	-1.362574	-0.275462	-0.691292
C	-2.798254	-2.099202	0.868863
H	-0.921534	-2.950628	1.427776
C	-2.740404	-0.192691	-0.682334
H	-0.796089	0.390565	-1.331956
C	-3.451943	-1.107372	0.120301
H	-3.378225	-2.780467	1.480357
H	-4.536028	-1.04993	0.151446
N	1.5485	2.141368	-0.670038
C	-3.476912	0.832627	-1.498857
H	-4.140722	0.346966	-2.219999
H	-4.096347	1.461169	-0.852316
H	-2.784839	1.47639	-2.044657

Table S14. Cartesian coordinates of the optimized structure for the radical cation of **1i** in Figure S2.

Symbol	X	Y	Z
C	1.167804	-1.025944	1.042021
C	1.511846	-0.044985	0.099895
C	3.354727	-1.230912	-0.567833
C	3.07705	-2.272891	0.324665
C	1.969634	-2.158984	1.151564
H	0.312412	-0.892169	1.693639
H	4.221226	-1.285978	-1.220505
H	3.728627	-3.137751	0.367932
H	1.730098	-2.931109	1.873956
C	0.717904	1.190945	-0.042086
C	1.387345	2.407066	-0.170133
C	-0.719928	1.187776	0.047914
C	0.688273	3.604307	-0.117691
H	2.467553	2.40368	-0.25483
C	-1.39906	2.43045	0.165572
C	-0.709895	3.614609	0.066988
H	1.225849	4.543457	-0.18926
H	-2.480028	2.44298	0.240213
H	-1.24217	4.557826	0.107143
C	-1.494637	-0.021632	-0.08425
C	-1.085521	-1.063493	-0.971489
C	-2.716609	-0.177015	0.62872
C	-1.8507	-2.203896	-1.11441
H	-0.20065	-0.930956	-1.582009
C	-3.450215	-1.335185	0.50539
H	-3.037475	0.592775	1.321041
C	-3.023912	-2.351297	-0.369153
H	-1.544522	-2.977632	-1.808456
H	-4.356976	-1.466973	1.084226
H	-3.618509	-3.252583	-0.472237
N	2.607133	-0.137011	-0.676424

Table S15. Cartesian coordinates of the optimized structure for the radical cation of **1j** in Figure S2.

Symbol	X	Y	Z
C	-2.131942	-2.547734	-0.203866
C	-3.498166	-2.634291	-0.431305
C	-4.265101	-1.470621	-0.495632
C	-3.663858	-0.229025	-0.317958
C	-2.292665	-0.124207	-0.078018
C	-1.509526	-1.299302	-0.043892
H	-1.529555	-3.450415	-0.195817
H	-3.961804	-3.604775	-0.570487
H	-5.333284	-1.531519	-0.675056
H	-4.260872	0.676446	-0.342887
C	-1.710741	1.219206	0.195501
C	-0.905488	1.440453	1.319198
C	-1.495175	3.405511	-0.465042
C	-0.369483	2.70742	1.523992
H	-0.716919	0.639266	2.025417
C	-0.660861	3.712017	0.608799
H	-1.756386	4.169334	-1.192322
H	0.259066	2.90411	2.385854
H	-0.263988	4.714171	0.722805
C	-0.044975	-1.251058	0.098017
C	0.638045	-2.152531	0.926838
C	0.70994	-0.305089	-0.650186
C	2.015469	-2.092179	1.050175
H	0.079976	-2.878041	1.507279
C	2.103602	-0.231317	-0.542794
H	0.198127	0.35566	-1.34236
C	2.782031	-1.131923	0.330435
H	2.526231	-2.777223	1.719328
C	2.848847	0.722728	-1.27762
C	4.17244	-1.050827	0.44783
C	4.232477	0.786703	-1.141855
H	2.330175	1.405654	-1.942619
C	4.889711	-0.093981	-0.284473
H	4.698935	-1.732638	1.107688
H	4.797384	1.520771	-1.704145
H	5.96763	-0.043568	-0.180663
N	-2.014598	2.193294	-0.67584

Table S16. Cartesian coordinates of the optimized structure for **2a** in Figure S6.

Symbol	X	Y	Z
C	-3.494104	-2.437895	0.016788
C	-2.386072	-3.282064	-0.142564
C	-1.131623	-2.733609	-0.170416
C	-1.988662	-0.519342	0.059357
C	-3.281145	-1.077879	0.098771
H	-4.500635	-2.838648	0.050268
H	-2.490859	-4.353683	-0.257759
H	-0.263906	-3.349736	-0.326389
H	-4.130707	-0.417624	0.183515
C	-2.848746	1.821944	0.14766
C	-1.761847	0.919109	0.063461
C	-0.447364	1.431843	-0.065883
C	-0.266732	2.828097	-0.147845
C	-1.345248	3.695117	-0.078637
C	-2.645116	3.189012	0.080905
H	-3.861922	1.462027	0.263389
H	0.725727	3.243599	-0.263799
H	-1.18048	4.765771	-0.142835
H	-3.492203	3.863433	0.146834
C	0.436832	-0.894275	0.008369
C	1.520561	-1.77848	0.134909
C	2.827966	-1.318898	0.126726
C	3.079885	0.05919	-0.002169
C	2.007092	0.94465	-0.07884
C	0.676634	0.498765	-0.064438
H	1.379717	-2.840756	0.27172
H	3.632903	-2.034096	0.234761
H	2.237805	2.00007	-0.133147
N	-0.91189	-1.383027	-0.030313
O	4.3163	0.612035	-0.033717
C	5.464799	-0.244798	0.053186
H	6.326769	0.417998	-0.010209
H	5.482345	-0.780047	1.007849
H	5.485606	-0.956323	-0.778312

Table S17. Cartesian coordinates of the optimized structure for **2b** in Figure S7.

Symbol	X	Y	Z
C	-2.705029	1.948911	-0.133456
C	-1.708433	0.954027	-0.064309
C	-0.105785	2.704824	0.203814
C	-1.07129	3.673252	0.153399
C	-2.406916	3.291359	-0.043397
H	-3.734658	1.646208	-0.248073
H	0.923166	2.961382	0.382985
H	-0.781245	4.709261	0.276571
H	-3.195987	4.032477	-0.100157
C	-3.678922	-2.252089	-0.11853
C	-2.654606	-3.198746	0.054429
C	-1.335442	-2.788492	0.14661
C	-0.989901	-1.421029	0.073595
C	-2.026115	-0.463759	-0.072202
C	-3.367677	-0.906258	-0.177566
H	-4.712569	-2.570869	-0.200017
H	-2.894018	-4.255734	0.111952
H	-0.566655	-3.539673	0.273853
H	-4.176977	-0.199848	-0.302279
C	0.390229	-0.961943	0.099296
C	1.482504	-1.854667	0.127889
C	2.79745	-1.429347	0.075713
C	3.067678	-0.052838	-0.033036
C	2.013387	0.861025	-0.056017
C	0.689289	0.41885	0.037233
H	1.299493	-2.919728	0.179465
H	3.59511	-2.160648	0.097618
H	2.285071	1.898891	-0.178186
N	-0.39234	1.367012	0.056332
O	4.308183	0.48129	-0.133068
C	5.445238	-0.395721	-0.139264
H	6.312408	0.25554	-0.239554
H	5.512018	-0.955518	0.798824
H	5.401799	-1.085302	-0.98801

Table S18. Cartesian coordinates of the optimized structure for **2d** in Figure S8.

Symbol	X	Y	Z
C	3.640954	2.775209	0.268157
C	2.453173	3.515214	0.144599
C	1.237667	2.863058	0.020831
C	1.165192	1.453851	0.010572
C	2.36618	0.706387	0.108239
C	3.596767	1.392856	0.247518
H	4.594013	3.282123	0.375103
H	2.483548	4.599963	0.151274
H	0.336456	3.456283	-0.063817
H	4.528158	0.850567	0.337757
C	2.328935	-0.744324	0.017535
C	3.497337	-1.531901	0.030455
C	1.086498	-2.749079	-0.360543
C	3.45968	-2.899547	-0.138795
H	4.451147	-1.044989	0.16473
C	2.219912	-3.516502	-0.363372
H	0.123117	-3.183481	-0.562054
H	4.375707	-3.478865	-0.123288
H	2.13217	-4.579371	-0.550706
C	-0.1027	0.738942	-0.048456
C	-0.123161	-0.671552	-0.058446
C	-1.342635	1.410829	-0.059029
C	-1.34472	-1.360067	0.029383
C	-2.552588	0.738678	-0.042359
H	-1.385102	2.49165	-0.084039
C	-2.551167	-0.676426	0.05172
H	-1.410193	-2.433748	0.130699
N	1.114865	-1.392599	-0.129888
O	-3.704436	-1.40658	0.121365
O	-3.709817	1.467064	-0.032514
C	-4.499032	-1.233573	1.318823
H	-5.368879	-1.877306	1.189928
H	-3.925234	-1.554325	2.194095
H	-4.81431	-0.194118	1.434956
C	-4.573891	1.303004	-1.181179
H	-5.436496	1.942512	-0.994858
H	-4.055019	1.63336	-2.086699
H	-4.893502	0.263709	-1.288576

Table S19. Cartesian coordinates of the optimized structure for **2e** in Figure S9.

Symbol	X	Y	Z
C	-3.666636	-2.646913	0.377482
C	-2.584884	-3.4822	0.056858
C	-1.329817	-2.93838	-0.168357
C	-1.114586	-1.546805	-0.093511
C	-2.219669	-0.701109	0.165714
C	-3.486366	-1.2752	0.423995
H	-4.645247	-3.069431	0.579103
H	-2.724236	-4.556706	-0.003544
H	-0.509181	-3.60607	-0.395913
H	-4.338878	-0.652842	0.661394
C	-2.060306	0.742355	0.061759
C	-3.156881	1.621435	0.083402
C	-0.686209	2.592406	-0.549002
C	-3.021036	2.96764	-0.193159
H	-4.138774	1.221948	0.287871
C	-1.758101	3.448825	-0.557135
H	0.297715	2.917824	-0.832986
H	-3.88486	3.622121	-0.169845
H	-1.599913	4.476888	-0.858798
C	0.223994	-0.958086	-0.177517
C	0.368697	0.449565	-0.092759
C	1.364062	-1.768493	-0.226758
C	1.671064	0.993457	0.117144
C	2.638091	-1.213405	-0.143207
H	1.287166	-2.844463	-0.29324
C	2.79075	0.164721	0.063351
H	3.771812	0.590915	0.205644
N	-0.801932	1.273104	-0.172139
O	3.674779	-2.081479	-0.227345
O	1.778278	2.314281	0.403814
C	3.060123	2.879106	0.725782
H	3.49075	2.386173	1.602097
H	3.741906	2.808881	-0.12665
H	2.859053	3.925425	0.950765
C	5.017616	-1.585782	-0.124969
H	5.192846	-1.128127	0.853935
H	5.658552	-2.459008	-0.239239
H	5.230911	-0.866481	-0.92205

Table S20. Cartesian coordinates of the optimized structure for **2g** in Figure S10.

Symbol	X	Y	Z
C	-3.47514	1.530305	0.085189
C	-2.305788	0.744309	0.04871
C	-1.06203	2.765772	-0.216383
C	-2.195755	3.53204	-0.191109
C	-3.43816	2.904432	-0.014946
H	-4.429713	1.035973	0.181788
H	-0.098092	3.211962	-0.385591
H	-2.106139	4.603284	-0.321458
H	-4.355114	3.481559	0.017162
C	-2.343983	-0.708121	0.076159
C	-3.575516	-1.399459	0.186695
C	-1.143036	-1.451106	-0.052424
C	-3.621734	-2.780889	0.147769
H	-4.505938	-0.860202	0.302615
C	-1.218316	-2.860184	-0.102916
C	-2.434002	-3.515817	-0.007931
H	-4.575164	-3.291149	0.233523
H	-0.318027	-3.450657	-0.213005
H	-2.46516	-4.599877	-0.048137
C	0.125589	-0.737171	-0.081088
C	0.147339	0.675195	-0.022613
C	1.360744	-1.413619	-0.120543
C	1.36811	1.35998	0.087434
C	2.56781	-0.73987	-0.073394
H	1.401495	-2.49269	-0.187179
C	2.568939	0.668699	0.069506
H	1.439495	2.428262	0.23158
O	3.72366	1.3767	0.192742
O	3.72457	-1.454111	-0.124862
C	4.909145	-0.663422	-0.364406
H	5.755455	-1.303744	-0.113755
H	4.951101	-0.400392	-1.427211
C	4.887879	0.578595	0.501257
H	5.750682	1.214287	0.300211
H	4.865449	0.316341	1.564745
N	-1.089823	1.398331	-0.058305

Table S21. Cartesian coordinates of the optimized structure for **2h** in Figure S11.

Symbol	X	Y	Z
C	-3.045717	-0.805364	0.09367
C	-1.703466	-0.378241	0.053751
C	-1.071273	-2.669373	-0.158273
C	-2.373982	-3.089216	-0.131815
C	-3.392833	-2.137187	0.01781
H	-3.825215	-0.063429	0.175063
H	-0.270021	-3.372502	-0.299938
H	-2.585083	-4.14606	-0.237
H	-4.434601	-2.434839	0.051613
C	-1.336491	1.029127	0.056615
C	-2.329622	2.035929	0.12744
C	0.024999	1.406807	-0.057614
C	-1.989666	3.375202	0.064091
H	-3.375259	1.779141	0.228929
C	0.344293	2.77927	-0.137662
C	-0.643345	3.748635	-0.079764
H	-2.765652	4.13121	0.119303
H	1.373694	3.094921	-0.245054
H	-0.372244	4.797585	-0.142166
C	1.047324	0.366177	-0.049389
C	0.674469	-0.993279	0.007249
C	2.425888	0.675751	-0.049632
C	1.666991	-1.985602	0.119683
C	3.414988	-0.295294	0.020919
H	2.732629	1.712971	-0.091423
C	3.006472	-1.640034	0.121967
H	1.423683	-3.031467	0.235928
H	3.749563	-2.42598	0.216416
C	4.878695	0.064881	0.010888
H	5.389309	-0.348827	0.887119
H	5.373211	-0.348448	-0.875492
H	5.024244	1.147687	0.007759
N	-0.717802	-1.345873	-0.030096

Table S22. Cartesian coordinates of the optimized structure for **2i** in Figure S12.

Symbol	X	Y	Z
C	0.365949	2.80163	0.000001
C	0.211438	1.400518	0
C	-2.147459	1.75119	-0.000001
C	-2.002995	3.112169	0
C	-0.712377	3.659233	0
H	1.362564	3.214755	0.000001
H	-3.127067	1.310633	-0.000001
H	-2.891162	3.731553	-0.000001
H	-0.560118	4.732389	0.000001
C	1.353803	0.5006	0
C	2.676103	1.007697	0
C	1.151623	-0.901503	0
C	3.764971	0.154995	0
H	2.86452	2.07222	0
C	2.27912	-1.750571	0
C	3.564356	-1.234805	0
H	4.76944	0.564468	0
H	2.154337	-2.825071	-0.000001
H	4.414849	-1.908754	0
C	-0.212262	-1.417835	0
C	-1.314272	-0.532641	0
C	-0.479175	-2.806025	0
C	-2.626907	-1.042153	0
C	-1.770411	-3.301181	0
H	0.343875	-3.507819	0
C	-2.851001	-2.409843	0
H	-3.496506	-0.403436	0
H	-1.941116	-4.372409	0
H	-3.870974	-2.778546	0
N	-1.075095	0.888739	0

Table S23. Cartesian coordinates of the optimized structure for **2j** in Figure S13.

Symbol	X	Y	Z
N	0.18475	1.17699	-0.205383
C	1.631302	-1.236724	-0.128638
C	-1.965591	-0.076042	0.074105
C	-0.458042	2.310301	-0.653909
H	-1.45963	2.166861	-1.024467
C	0.184906	-1.242726	-0.308213
C	2.271278	-0.018173	0.219439
C	-0.540894	-2.447906	-0.55085
H	-0.00305	-3.366878	-0.741649
C	-2.653429	-1.3056	-0.191913
C	-0.539227	-0.055891	-0.150082
C	1.539591	1.225458	0.077029
C	3.635037	-0.027589	0.591864
H	4.128832	0.884467	0.901484
C	0.151618	3.536281	-0.6653
H	-0.401106	4.393164	-1.03008
C	2.403962	-2.41732	-0.170153
H	1.949212	-3.359226	-0.447802
C	3.747935	-2.401417	0.167565
H	4.320495	-3.32269	0.135261
C	4.363229	-1.204286	0.571513
H	5.408327	-1.1973	0.862132
C	-2.721139	0.98946	0.641933
H	-2.231691	1.896874	0.973189
C	2.164226	2.487293	0.099926
H	3.215763	2.541844	0.341298
C	1.483172	3.64015	-0.232187
H	1.987751	4.599358	-0.211834
C	-4.059641	-1.378587	-0.01814
H	-4.561669	-2.312386	-0.253373
C	-1.907473	-2.462569	-0.557112
H	-2.44115	-3.382113	-0.776931
C	-4.083143	0.873534	0.840947
H	-4.625208	1.6973	1.294312
C	-4.770319	-0.305001	0.475605
H	-5.844264	-0.374075	0.615566

10. Supporting references

[1] Rao, Y. L.; Amarne, H.; Zhao, S. B.; McCormick, T. M.; Martić, S.; Sun, Y.; Wang, R. Y.; Wang, S. *J. Am. Chem. Soc.* **2008**, *130*, 12898–12900.

[2] Li, B.; Wu, Z. H.; Gu, Y. F.; Sun, C. L.; Wang, B. Q.; Shi, Z. J. *Angew. Chem. Int. Ed.* **2011**, *50*, 1109–1113.

[3] Lyons, T. W.; Hull, K. L.; Sanford, M. S. *J. Am. Chem. Soc.* **2011**, *133*, 4455–4464.

[4] Zhang, J.; Yang, Q.; Zhu, Z.; Yuan, M. L.; Fu, H. Y.; Zheng, X. L.; Chen, H.; Li, R. X. *Eur. J. Org. Chem.* **2012**, 6702–6706.

[5] Zhao, T. T.; Xu, W. H.; Zheng, Z. J.; Xu, P. F.; Wei, H. *J. Am. Chem. Soc.* **2018**, *140*, 586–589.

[6] Ji, J.; Jiang, L.; Wang, Z.; Bin, Z.; You, J.; Yang, Y. *Org. Lett.* **2022**, *24*, 6256–6260.
